# Genome sequence of the corn leaf aphid (*Rhopalosiphum maidis* Fitch)

**DOI:** 10.1093/gigascience/giz033

**Published:** 2019-04-06

**Authors:** Wenbo Chen, Sara Shakir, Mahdiyeh Bigham, Annett Richter, Zhangjun Fei, Georg Jander

**Affiliations:** 1 Boyce Thompson Institute, 533 Tower Rd, Ithaca, NY 14853, USA; 2 US Department of Agriculture–Agricultural Research Service, Robert W. Holley Center for Agriculture and Health, 538 Tower Rd, Ithaca, NY 14853, USA

**Keywords:** corn leaf aphid, genome, annotation, *Rhopalosiphum maidis*

## Abstract

**Background:**

The corn leaf aphid (*Rhopalosiphum maidis* Fitch) is the most economically damaging aphid pest on maize (*Zea mays*), one of the world's most important grain crops. In addition to causing direct damage by removing photoassimilates, *R. maidis* transmits several destructive maize viruses, including maize yellow dwarf virus, barley yellow dwarf virus, sugarcane mosaic virus, and cucumber mosaic virus.

**Findings:**

The genome of a parthenogenetically reproducing *R. maidis* clone was assembled with a combination of Pacific Biosciences (207-fold coverage) and Illumina (83-fold coverage) sequencing. The 689 assembled contigs, which have an N50 size of 9.0 megabases (Mb) and a low level of heterozygosity, were clustered using Phase Genomics Hi-C interaction maps. Consistent with the commonly observed 2n = 8 karyotype of *R. maidis*, most of the contigs (473 spanning 321 Mb) were successfully oriented into 4 scaffolds. The genome assembly captured the full length of 95.8% of the core eukaryotic genes, indicating that it is highly complete. Repetitive sequences accounted for 21.2% of the assembly, and a total of 17,629 protein-coding genes were predicted with integrated evidence from *ab initio* and homology-based gene predictions and transcriptome sequences generated with both Pacific Biosciences and Illumina. An analysis of likely horizontally transferred genes identified 2 from bacteria, 7 from fungi, 2 from protozoa, and 9 from algae. Repeat elements, transposons, and genes encoding likely detoxification enzymes (cytochrome P450s, glutathione S-transferases, carboxylesterases, uridine diphosphate–glucosyltransferases, and ABC transporters) were identified in the genome sequence. Other than *Buchnera aphidicola* (642,929 base pairs, 602 genes), no endosymbiont bacteria were found in *R. maidis*.

**Conclusions:**

A high-quality *R. maidis* genome was assembled at the chromosome level. This genome sequence will enable further research related to ecological interactions, virus transmission, pesticide resistance, and other aspects of *R. maidis* biology. It also serves as a valuable resource for comparative investigation of other aphid species.

## Data Description

### Introduction

Maize (*Zea mays*), the world's most productive grain crop, is susceptible to >90 species of herbivorous insects [1–3]. Among aphids that feed on maize, the corn leaf aphid (*Rhopalosiphum maidis* Fitch) is the most commonly encountered, particularly in tropical and warmer temperate areas [4]. Relative to other maize-feeding aphids (*Rhopalosiphum padi, Schizaphis graminum, Sitobion avenae*, and *Metopolophium dirhodum*), *R. maidis* exhibits a greater tolerance of benzoxazinoids, the most abundant class of maize defensive metabolites [5]. However, the mechanism of aphid resistance to these plant toxins is not known, and natural variation in benzoxazinoid content among maize inbred lines nevertheless influences the growth and reproduction of *R. maidis* [6, 7].

Damage caused to maize by *R. maidis* takes several forms, with the resulting yield losses being quite variable from year to year. Growth and yield are reduced through the removal of photosynthates by large numbers of aphids [8]. On flowering-stage maize, aphids tend to congregate on the tassels, where large amounts of honeydew can prevent the release of pollen from the anthers, thereby reducing seed set by up to 90% [9, 10]. Additional damage comes from the transmission of several important maize viruses, including maize yellow dwarf virus, barley yellow dwarf virus, sugarcane mosaic virus, and cucumber mosaic virus [11–15], by *R. maidis*.

In addition to feeding on maize, *R. maidis* infests a variety of other monocot species, including barley, oat, rice, rye, sorghum, sugarcane, and wheat [4]. In 1 study, barley was reported as the most suitable grain crop host [16]. However, as in the case of maize, there is also considerable within-species variation for *R. maidis* resistance in barley [17].

The origin of *R. maidis* is likely in Asia, and it has been subsequently introduced in most grain-growing areas of the world [4]. In almost all parts of its range, *R. maidis* is anholocyclic, i.e., reproduction occurs entirely by parthenogenesis. However, sexual reproduction has been reported in Pakistan and Korea, with *Prunus* ssp. as the primary host [18, 19]. In populations in Japan and Kenya, males but not sexually reproducing females have been found [20, 21]. Consistent with the sometimes permanently parthenogenetic life cycle of *R. maidis*, there is within-species variation in the chromosome numbers. Karyotypes of 2n = 8, 9, and 10 have been reported. There also is evidence of host specificity among the karyotypes. Whereas *R. maidis* strains on maize tend to have 2n = 8, those on barley generally have 2n = 10 [22, 23].

Here we report the *R. maidis* isolate BTI-1 genome sequence, assembled using long-read Pacific Biosciences (PacBio) sequencing, Illumina sequencing, and Phase Genomics Hi-C scaffolding. Contigs were assembled using PacBio, which provides >10,000 base pair (bp) read lengths but has a 10% error rate [24]. These errors were corrected by Illumina sequencing reads, with 151-bp paired-end read lengths and a 0.1% error rate. Contigs assembled from PacBio sequencing were linked into chromosome-scale scaffolds using Hi-C, which identifies long-range contact information for DNA sequences [25, 26]. To assist in annotation of the *R. maidis* genome, we sequenced complementary DNA libraries using both PacBio sequencing to get full-length transcripts (Iso-Seq) and Illumina RNA sequencing (RNA-Seq) to get a higher sequence accuracy. Comparisons to 6 previously published aphid genomes [27–33] showed an improved assembly, with most of the sequences assembled into 4 scaffolds, consistent with the 2n = 8 karyotype of *R. maidis* on maize. Analysis of the assembled *R. maidis* genome identified horizontally transferred genes, repetitive elements, and likely xenobiotic detoxification enzymes.

### Sampling and genome sequencing

#### Insect colony

BTI-1, a corn leaf aphid (*R. maidis*; NCBI:txid43146) isolate, which was originally collected from maize (*Z. mays*) in New York State, was obtained from Stewart Gray (US Department of Agriculture Plant Soil and Nutrition Laboratory, Ithaca, NY, USA). An isogenic colony was started from a single parthenogenetic female *R. maidis* and was maintained on barley (*Hordeum vulgare*) prior to the collection of insects for genome and transcriptome sequencing.

#### Genome sequencing

Genomic DNA was prepared from 100–200 mg of fresh mixed-instar *R. maidis* tissue using a previously described protocol [34]. Briefly, mixed-instar whole aphids collected from barley were ground in liquid nitrogen with a mortar and pestle and then incubated at 65°C in 400 µl microprep buffer made up of DNA extraction buffer (0.35 M sorbitol, 0.1 M Tris-base, pH 7.5; 5 mM ethylenediaminetetraacetic acid [EDTA]), nuclei lysis buffer (0.2 M Tris-base, pH 7.5; 0.05 M EDTA; 2 M NaCl; 2% cetyltrimethylammonium bromide), 5% sarkosyl, and 0.5% sodium bisulfite (added right before use) for 30 min. The cooled-down solution was then treated with 400 µl chloroform: isoamyl alcohol (24:1), vortexed vigorously, and centrifuged for 10 min at 14,000*g*. To the upper aqueous phase 3 µl of Rnase A was added and the samples were incubated for 15 min at 37°C followed by adding 400 µl chloroform: isoamyl alcohol (24:1), vortexing vigorously, and repeating the centrifugation step for 10 min at 14,000*g*. To precipitate the DNA, 200 µl of ice-cold 2-propanol was added and gently inverted. The DNA was pelleted by centrifugation at 4°C at 14,000*g* for 10 min. The DNA pellet was washed with 70% ethanol and, after air drying, was dissolved in 50 μl of nuclease-free water. The quantity and quality of aphid genomic DNA was assessed using a Qubit 3 fluorometer (Thermo Fisher, Waltham, MA, USA) and a Bioanalyzer DNA12000 kit (Agilent, Santa Clara, CA, USA), respectively. Approximately 20 μg of DNA was used for PacBio library construction and sequencing according to the manufacturer's (PacBio, Menlo Park, CA, USA) instructions for single-molecule real-time sequencing (SMRT) 20 kb DNA template preparation, using the PacBio Sequel 2.0 sequencing enzyme and chemistry, respectively. Briefly, aphid DNA was first re-purified using a 0.5 × AMPure XP (Beckman Coulter, Indianapolis, IN, USA) purification step (0.5 × AMPure beads added, by volume, to the DNA sample dissolved in 150 μl Elution Buffer, vortexed for 10 min at 2,000*g*, followed by 2 washes with 70% alcohol and finally diluted in Elution Buffer), to remove small fragments and/or biological contaminants. The DNA was then sheared to 25–30 kb using a Covaris G-tube (Covaris, Wobern, MA, USA) and an Eppendorf 5424 centrifuge (Eppendorf, Hamburg, Germany) at 3,000*g*. The DNA was further purified with 0.5 × AMPure XP and the average fragment size was assessed with the Agilent Bioanalyzer DNA12000 kit. The purified DNA sample was taken through DNA damage and end-repair steps. Briefly, the DNA fragments, after processing with 0.18 U/μl of P6 polymerase, were repaired using DNA damage repair solution (1 × DNA damage repeat buffer, 1 × nicotinamide adenine dinucleotide, 1 mM adenosine triphosphate [ATP] high, 0.1 mM dNTP, and 1 × DNA damage repeat mix) in a volume of 52 μl and incubated at 37°C for 20 min. DNA ends were repaired by adding 1 × end repair mix to the solution, which was incubated at 25°C for 10 min, followed by a second 0.45 × Ampure XP purification step.

**Figure 1:**
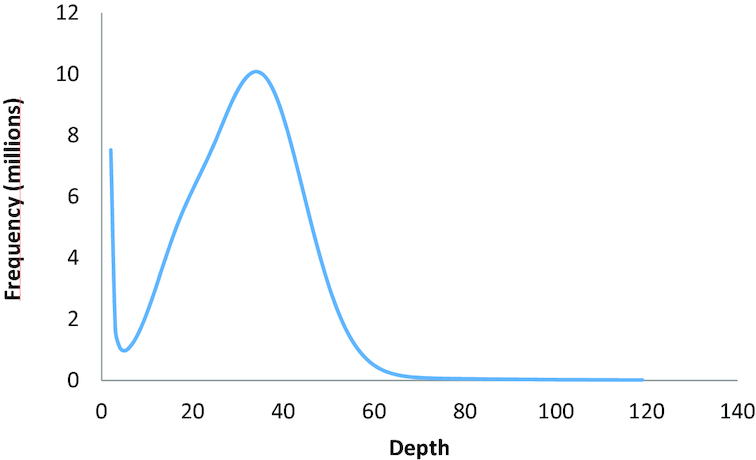
K-mer (K = 31) distribution of Illumina genome sequencing reads of *R. maidis*. The total count of k-mers was 11,495,021,417, and the peak of k-mer depth was 34. The genome size of *R. maidis* was calculated by dividing the total k-mer count by the peak depth, which was ∼338 Mb. The single peak of the k-mer distribution profile indicates that the *R. maidis* genome has a low level of heterozygosity.

SMRTbell library preparation (PacBio) was performed as follows: 0.75 μM of blunt adapter was added to the DNA, followed by 1 × template preparation buffer, 0.05 mM ATP low, and 0.75 U/μl T4 ligase to ligate the SMRTbell adapters to the DNA fragments in the final volume of 40 μl. This solution was incubated at 25°C overnight, followed by a 10-min ligase denaturation step at 65°C. After ligation, the library was treated with an exonuclease cocktail to remove unligated DNA fragments using a solution of 1.81 U/μl Exonuclease III and 0.18 U/μl Exonuclease VII and then incubated at 37°C for 1 h. Two additional 0.45 × Ampure XP purification steps were performed to remove <1,000-bp molecular-weight DNA and organic contaminants. Further size selection of 17–50 kb was performed on Sage BluePippin (Sage Science, Beverly, MA, USA). The 0.75% agarose gel was run for 4.5 h using the manufacturer's protocol. An additional DNA damage repair step was performed after the size selection by incubating the template at 37°C for 30 min, followed by 0.8 × AMP bead purification. The size of the resulting fragments was confirmed using the Agilent bioanalyzer DNA12000 kit, and the mass was quantified using an Invitrogen Qubit 3 fluorometer (Thermo Fisher) before proceeding with primer annealing, polymerase and DNA sequencing on a Sequel system (PacBio). Polymerase 2.0 binding to the template was performed by incubating for 4 h at 30°C. The binding complex was then diluted and diffusion-loaded at 6 pM on the plate on a Sequel 5.0 system and sequenced on a Sequel machine with 2.0 chemistry recording 10-h movies. Sequencing was conducted at the Icahn Institute and Department of Genetics and Genomic Sciences, Icahn School of Medicine at Mount Sinai (New York, NY, USA). The raw data analysis was performed on SMRTLink 5.0. A total of 16 SMRT cells were run on the PacBio Sequel platform, yielding 70 gigabases (Gb) raw sequence data (Supplementary Data S1) for the *R. maidis* genome, which was initially estimated to be 338 megabases (Mb) using the k-mer approach [35] (Fig. 1).

For short-read sequencing, 1 paired-end library was constructed using the Illumina TruSeq DNA sample preparation kit (Illumina, San Diego, CA, USA), following the manufacturer's instructions. The quantity of DNA was measured using an Invitrogen Qubit 3 fluorometer, and ∼2 μg of DNA was normalized in resuspension buffer in a volume of 55 μl and vortexed at 1,800*g* for 2 min followed by centrifugation at 280*g* for 1 min. For fragmentation, 52.5 µl of the DNA samples were transferred to a Covaris microTUBE and centrifuged at 280*g* for 5 s. The DNA was purified using sample purification beads, which were washed twice with 80% ethanol and dissolved in 50 μl of resuspension buffer. Purified DNA was end-repaired by adding 40 μl of end repair mixture and incubating at 30°C for 30 min. The library with an insert size of ∼550 bp was purified using sample purification beads (SPBs) and all the remaining large and small DNA fragments were removed according to the manufacturer's protocol. Briefly, to remove large DNA fragments, 92 μl of SPBs were diluted in 92 μl of polymerase chain reaction–grade water, vortexed, and 160 μl of this solution was added to the purified DNA, vortexed at 1,800*g* for 2 min, incubated at 23°C for 5 min, and centrifuged again at 280*g* for 1 min. Two hundred fifty μl of supernatant were transferred to a cleanup end repair plate. To remove small DNA fragments, 30 μl of SPBs were added to the supernatant, vortexed at 1,800*g* for 2 min, incubated at 23°C for 5 min, and centrifuged at 280*g* for 1 min. The supernatant was discarded, SPBs were washed twice with 80% ethanol, and DNA was dissolved in 15 μl of resuspension buffer. Purified DNA was adenylated at the 3′ ends by adding 2.5 μl of A-Tailing control and 12.5 μl of A-Tailing mixture (New England Biolabs, Ipswich, MA, USA) to the sample with a final volume of 30 μl, and vortexed at 1,800*g* for 2 min followed by a first incubation at 37°C for 30 min, a second incubation at 70°C for 5 min, and a final incubation on ice for 5 min.

A TruSeq polymerase chain reaction–free library (Illumina) was prepared as follows: 2.5 μl of adapters were added to the DNA, followed by adding 2.5 μl of ligation mixture, 2.5 μl of ligation control, and incubation at 30°C for 10 min. The ligation step was stopped by adding 5 μl of ligation stop buffer and ligated fragments were purified using SPBs, which were washed twice with 80% ethanol. The library was quantified with the KAPA Library Quantification Kit (Roche, Basel, Switzerland), and the fragment size of the library was verified using an Agilent Technology 2100 bioanalyzer. Sequencing was performed on an Illumina HiSeq 2500 system, which yielded ∼75 Gb of raw sequence data (Supplementary Data S1). Raw Illumina reads were processed to remove duplicated read pairs, which were defined as having identical bases in the first 100 bp of both left and right reads, and only 1 read pair from each duplicated sequence was kept. Illumina adapters and low-quality sequences were removed from the reads using Trimmomatic (Trimmomatic, RRID:SCR_011848) [36], resulting in 28 Gb of usable sequencing reads. The k-mer depth distribution of the cleaned high-quality sequences displayed a single peak (Fig. 1), indicating that the *R. maidis* genome has a low level of heterozygosity.

### Transcriptome sequencing

Transcriptome sequencing (Illumina strand-specific RNA-Seq and PacBio Iso-Seq) was conducted to aid gene prediction. Mixed-instar aphids feeding on barley were collected for total RNA extraction using the SV Total RNA isolation kit (Promega, Madison, WI, USA). Briefly, cells were lysed by grinding 100–120 mg of insect tissue in liquid nitrogen using a mortar and pestle, followed by incubation at 70°C in RNA lysis buffer (4 M guanidine thiocyanate; 0.01 M Tris, pH 7.5; 0.97% β-mercaptoethanol) for 3 min. This solution was then centrifuged for 10 min at 14,000*g* and the supernatant was passed through a spin column provided with the kit, followed by DNase treatment. RNA was washed with RNA wash solution (60 mM potassium acetate; 10 mM Tris-HCl, pH 7.5; 60% ethanol) and dissolved in 50 μl of nuclease-free water. Strand-specific RNA-Seq libraries were constructed using a previously described protocol [37] and sequenced at the Biotechnology Resource Center of Cornell University (Ithaca, NY, USA) on an Illumina HiSeq 2500 sequencing system. More than 188 million paired-end reads with lengths of 151 bp were obtained (Supplementary Data S1). Raw reads were processed by trimming adapter and low-quality sequences using Trimmomatic [36]. The cleaned reads were aligned to the assembled *R. maidis* genome using HISAT2 (HISAT2, RRID:SCR_015530) [38], followed by reference-guided assembly using StringTie (StringTie, RRID:SCR_016323) [39]. The assembled transcripts were used to improve protein-coding gene predictions in the *R. maidis* genome.

For Iso-Seq, 20 μg RNA, isolated from 100–120 mg of fresh *R. maidis* tissue using the SV Total RNA isolation kit (Promega) with the method described above, was shipped to Duke Center for Genomic and Computational Biology (Durham, NC, USA) for PacBio large-insert library construction and sequencing using standard SMRTbell template preparation kits. The library insert size ranged from 500 to 4,500 bp. One SMRT cell was run on the PacBio Sequel platform, yielding ∼10 Gb raw sequence data (Supplementary Data S1). The PacBio raw reads were processed using IsoSeq3 [40]. Briefly, 1 representative circular consensus sequence was generated for each zero-mode waveguide. Only zero-mode waveguides with ≥1 full pass, meaning that each primer has been seen at least once, were used for the subsequent analysis. The circular consensus sequences were processed to remove the 5′ and 3′ primers, trim off polyA tails, and remove artificial concatemers to create full-length, non-concatemer reads. These reads were then clustered together. The final polishing step created a consensus sequence for each clustered transcript. A total of 21,114 high-quality transcripts were generated and used to support protein-coding gene predictions in the *R. maidis* genome.

### Hi-C library construction and sequencing

For Hi-C sequencing, 200 mg of *R. maidis* tissue was used for chromatin isolation and library preparation using the animal Hi-C kit from Phase Genomics (Seattle, WA, USA). Hi-C libraries were sequenced at the Biotechnology Resource Center at Cornell University (Ithaca, NY, USA) using the NextSeq500 platform (Illumina) to obtain 76 bp paired-end reads. Raw reads were processed by trimming adapter and low-quality sequences using Trimmomatic [36]. The cleaned Hi-C reads were aligned to the assembled contigs using BWA-aln [25], and the optimal placement of each read pair was determined by BWA-sampe [25]. Reads that did not map within 500 bp of a restriction enzyme site were removed using the PreprocessSAMs.pl script in LACHESIS [26]. Finally, only reads with mapping quality >30 were used for scaffolding by LACHESIS [26].

### Genome assembly

The PacBio long reads were corrected and assembled with the Canu assembler (Canu, RRID:SCR_015880) [41] (version 1.6). The resulting contigs were polished by aligning the raw PacBio reads to the assembly and correcting the sequencing errors using Arrow [42]. To further improve the assembly, another round of polishing was performed by aligning the Illumina short reads to the assembly and correcting the sequencing errors using Pilon (Pilon, RRID:SCR_014731) [43].

The assembled contigs were then compared against the NCBI non-redundant nucleotide database using BLASTN (BLASTN, RRID:SCR_001598) with the parameters "–dust yes –max_target_seqs 10 –evalue 1e–5 –outfmt '6 qseqid sseqid pident length mismatch gapopen qstart qend sstart send evalue bitscore qlen slen sstrand staxids sscinames sskingdoms stitle'" to identify contamination on the basis of taxonomy. This analysis identified sequences with homology to known sequences from archaea (*Methanobacterium formicicum*), bacteria (*Cronobacter dublinensis, Escherichia coli, Methylobacterium zatmanii, Planktothrix agardhii, Salmonella enterica*, and *Weissella cibaria*), eukaryotes (*Plasmodium falciparum, Brugia pahangi, Emplectanthus cordatus, Gossypium arboretum, Zea mays, Theobroma cacao, Frankliniella intonsa*, and *Oryzias latipes*), and plasmids. If >90% of an individual contig had likely non-aphid DNA, it was considered to be contamination. Altogether, we excluded 57 contigs, totaling 930,609 bp, from the assembly. Contigs were clustered by Phase Genomics Hi-C using LACHESIS [26] with default parameters. The resulting scaffolds were manually polished using Juicebox [44]. Because Hi-C data do not provide the exact number of bp between the oriented contigs, Phase Genomics LACHESIS arbitrarily adds 100 Ns between contigs.

**Figure 2:**
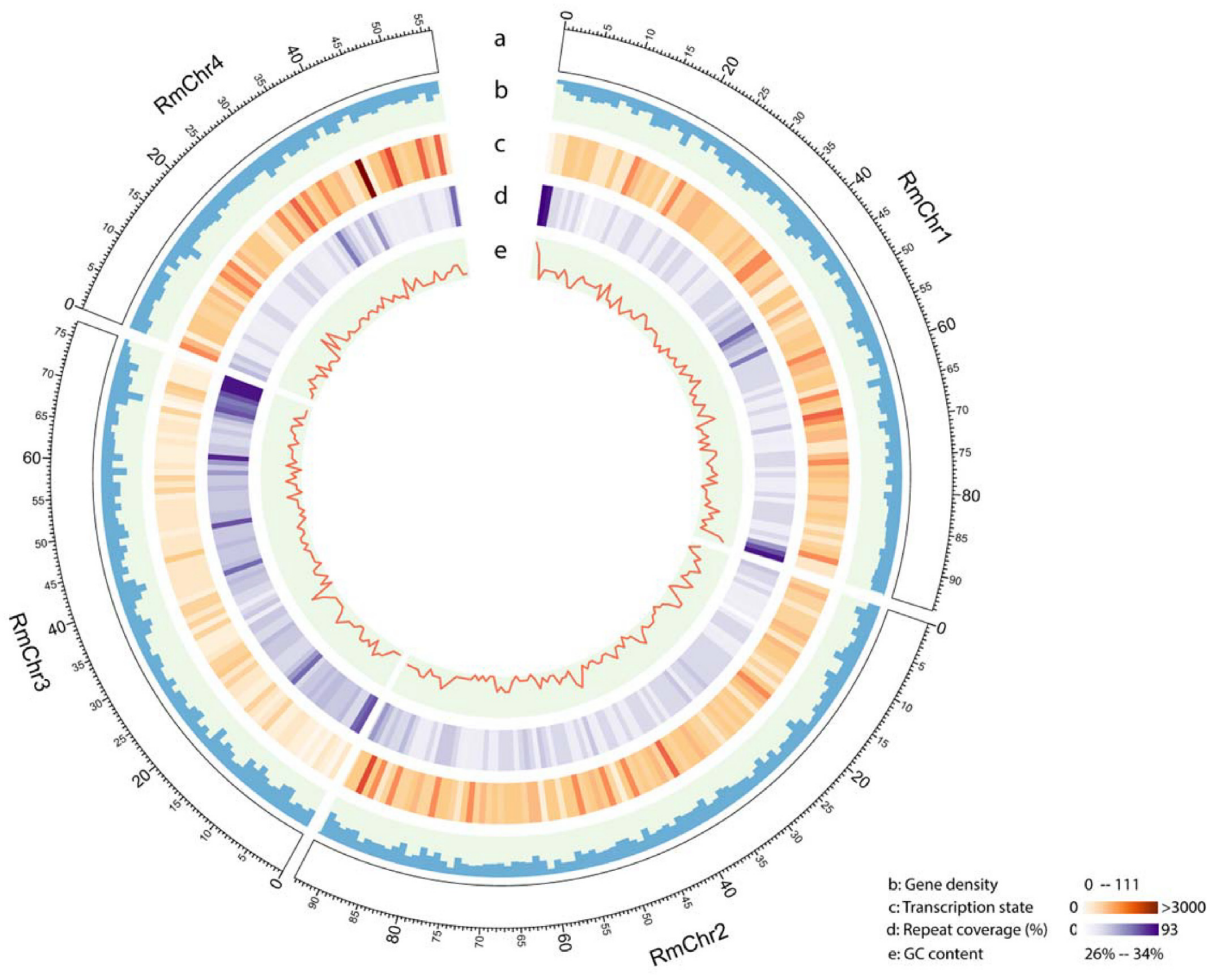
*R. maidis* genome landscape. (a) Ideogram of the 4 *R. maidis* pseudochromosomes at the Mb scale. (b) Gene density represented as number of genes per Mb. (c) Transcription state. The transcription level was estimated by read counts per million mapped reads in 1-Mb windows. (d) Percentage of coverage of repeat sequences per Mb. (e) Guanine-cytosine (GC) content in 1-Mb windows. The 4 *R. maidis* pseudo-chromosomes represented 99.3% of the genome assembly. This figure was generated using Circos (http://circos.ca/).

The assembled *R. maidis* genome, with a total length of 326.0 Mb, consisted of 689 contigs with an N50 length of 9.0 Mb. Thus, this is a much-improved genome assembly compared with the 6 previously published aphid genomes (Table 1). A total of 602 contigs spanning 323.4 Mb (99.2% of the assembly) were clustered into 4 groups, which was consistent with the commonly observed 2n = 8 karyotype of *R. maidis* [22]. Of the clustered contigs, 473 spanning 320.7 Mb (98.4% of the assembly) were successfully oriented (Fig. 2; Supplementary Fig. S1). To evaluate the completeness of the genome assembly, it was aligned to Illumina paired-end libraries, allowing up to 3 mismatches using BWA-MEM [25]. With this approach, 94.2% of the Illumina reads could be mapped back to the assembly, indicating that most of the reads were successfully assembled into the genome. RNA-Seq reads were also aligned to the genome assembly using HISAT2 [38], resulting in a mapping ratio of 94.1% (Supplementary Data S1). Furthermore, evaluation by Benchmarking Universal Single-Copy Orthologs (BUSCO, RRID:SCR_015008, version 3.0.2 [45] showed that 95.8% of the core eukaryotic genes were at least partially captured by the genome assembly and 94.5% were completely captured. The heterozygosity rate of the genome calculated by bbmap [46] is <0.00005, which is consistent with the profile of the k-mer distribution. The guanine-cytosine content of the genome is 27.7%, with 6.7% of total genome consisting of coding regions, 42.0% introns, and 51.2% intergenic regions. The 18,060-bp mitochondrial genome sequence was assembled separately (GenBank accession MK368778). Taken together, our evaluation indicated an overall high quality of the assembled *R. maidis* genome.

**Table 1:** Assembly statistics of 7 aphid genomes

Species	*R. maidis*	*A. glycines*	*M. persicae* *	*A. pisum*	*M. cerasi*	*R. padi*	*D. noxia* *
Sequencing source	[this study]	[33]	[29]	[27]	[28]	[28]	[31]
Genome assembly							
Assembly size (Mb)	326.0	302.9	347.3	541.6	405.7	319.4	393.0
Contig count	689	66,000	8,249	60,623	56,508	16,689	49,357
Contig N50 (bp)	9,046,396	15,844	144,275	28,192	17,908	96,831	12,578
Scaffold count	220	8,397	4,022	23,924	49,286	15,587	5,641
Scaffold N50 (bp)	93,298,903	174,505	435,781	518,546	23,273	116,185	397,774
Maximum scaffold length (Mb)	94.2	1.4	2.2	3	0.2	0.6	2.1
Minimum scaffold length (kb)	1.1	2	0.9	0.2	1	1	0.9
Genomic features							
Transcript length (bp)	1,834.6	1,520.1	1,838.7	1,964.1	NA	NA	NA
CDS length (bp)	1,242.04	1,240.3	1,328.3	1,157.6	952.7	1,155.09	970.2
Exon length (bp)	210.02	245.5	299.2	394.7	NA	NA	NA
Exon count/gene	6.31	6.19	6.14	4.97	NA	NA	NA
Gene counts [source]	17,629	19,182 [33]	18,529 [29]	36,195 [27]	28,408 [28]	28,542 [28]	19,097 [31]
	[this study]		25,726 [32]	27,676 [32]	28,688 [32]	26,286 [32]	25,987 [32]
			18,433 [29]				31,885 [30]
			23,822 [28]				
			24,742 [28]				
			21,441 [28]				

NA: This information could not be retrieved from the annotation files.

^*^More than 1 sequenced lineage.

Aphids have an X0 sex determination system, with males receiving only 1 copy of the sex chromosome. Segregation of sex chromosomes in *Myzus persicae* and *Acyrthosiphon pisum* has been studied extensively using simple sequence repeat markers [47–49]. We were not able to unambiguously assign the *R. maidis* X chromosome. Four *M. persicae* X chromosome markers [47] were assigned to 3 contigs on *R. maidis* chromosome 3. However, several *A. pisum* X chromosome markers [48, 49] were distributed across all 4 *R. maidis* chromosomes.

### Endosymbiont genomes

The genome sequence of the *Buchnera aphidicola* endosymbiont was separated from the *R. maidis* host genome sequences by aligning the initial assembly to the *B. aphidicola* reference genome from *A. pisum* (*Buchnera*APS; GeneBank ID: NC_002528.1, [50]). One single contig was extracted and polished using both PacBio long reads and Illumina short reads, as described above. Genome annotation was performed using prokka (Prokka, RRID:SCR_014732) [51]. The assembled *Buchnera*Rm genome had a length of 642,929 bp (Supplementary Fig. S2), with 602 predicted protein-coding genes. The 2 *Buchnera* plasmids, pLeu and pTrp, were also sequenced and assembled, with lengths of 7,852 and 3,674 bp, respectively (Supplementary Fig. S2).

To identify secondary bacterial symbionts in *R. maidis*, raw assembled contigs were compared against the reference sequences of previously identified secondary bacterial symbionts of aphids, including *Hamiltonella defensa, Regiella insecticola, Serratia symbiotica, Rickettsia, Spiroplasma*X‐type, *Sitobion miscanthi* L-type, *Arsenophonus*, and *Wolbachia* [52], using BLAST with the parameters" –dust yes –max_target_seqs 10 –evalue 1e–5 –outfmt '6 qseqid sseqid pident length mismatch gapopen qstart qend sstart send evalue bitscore qlen slen sstrand staxids sscinames sskingdoms stitle'". Although no hits were found, we cannot determine whether this absence of secondary endosymbionts is specific to our *R. maidis* isolate or whether it is a more general property of this species. Whereas some studies have found secondary symbionts, including *S. symbiotica, S. miscanthi*, and *H. defensa* in *R. padi* [53], a closely related aphid species, others have not [54].

### Annotation of repetitive elements

We identified miniature inverted-repeat transposable elements (MITEs) from the assembled *R. maidis* genome using MITE-Hunter [55] and then generated a *de novo* repeat library by scanning the assembled genome using RepeatModeler (RepeatModeler, RRID:SCR_015027) [56], which integrates results from RECON [57], TRF [58], and RepeatScout (RepeatScout, RRID:SCR_014653) [59] and classifies repeats with the RepBase library [60]. RepeatModeler identified 546 repeats, which were comparedagainst the NCBI non-redundant protein database using BLAST with an e-value cutoff of 1e–5. Those having hits to known protein sequences were excluded. Finally, we identified repeat sequences by scanning the assembled *R. maidis* genome using the *de novo* repeat library with RepeatMasker (RepeatMasker, RRID:SCR_012954) [61] and the RepeatRunner subroutine [62] in the MAKER annotation pipeline [63]. A total of 21.18% of the assembled *R. maidis* genome was annotated as repeat elements (Table 2). The most predominant repeat elements were unknown repeats and MITEs, which occupied 5.64% and 4.37% of the genome, respectively.

**Table 2:** Repeats in the *R. maidis* genome assembly

Class	No. of copies	Length (bp)	Coverage of genome (%)
SINE	27,308	7,085,803	2.17
LINE	6,688	1,596,259	0.49
Long terminal repeat	3,445	896,470	0.28
DNA transposon	53,797	9,499,710	2.91
MITE	64,663	14,240,430	4.37
Unclassified	49,627	18,375,079	5.64
Other*	375,149	17,360,944	5.33
Total	580,677	69,054,695	21.18

^*^Other includes microsatellites, simple repeats, and low-complexity sequences.

### Gene prediction

Protein-coding genes were predicted from the genome assembly of *R. maidis* using the automated pipeline MAKER (MAKER, RRID:SCR_005309) [63]. MAKER integrates the results from *ab initio* gene predictions with experimental gene evidence to produce a final consensus gene set. The evidence that was used included complete aphid coding sequences collected from NCBI [64], transcripts assembled from our strand-specific RNA-Seq data, high-quality transcript sequences from Iso-Seq, completed proteomes of *A. pisum, Aphis glycines, Diuraphis noxia, Myzus cerasi, M. persicae*, and *R. padi*, and proteins from the UniProt database. All of these sequences were aligned to the *R. maidis* genome using Spaln [65]. MAKER was used to run a battery of trained gene predictors, including Augustus (Augustus, RRID:SCR_008417) [66], BRAKER [67], and GeneMark-ET [68], and then integrated the experimental gene evidence to produce evidence-based predictions. Altogether, 17,629 protein-coding genes were predicted for the 4 *R. maidis* chromosomes and non-scaffolded contigs (Table 3). The gene count of *R. maidis* is similar to that of *A. glycines* [33] and *M. persicae* [29] but lower than other aphid genome annotations (Table 1). However, it should be noted that estimated gene counts can vary depending on both the quality of the DNA sequencing and the specific annotation pipeline that is used. A recent comparative analysis of 5 aphid genomes using the same annotation pipeline identified similar total numbers of genes, ranging from 25,726 to 27,676 (Table 1) [32].

**Table 3:** Gene distribution on the *R. maidis* chromosomes

Chromosome	Length (bp)	Gene count
**Chr1**	94,224,415	4,968
**Chr2**	93,298,903	5,124
**Chr3**	76,887,858	4,478
**Chr4**	56,292,413	3,034
**Not assigned**	5,319,566	25
**Total**	326,023,155	17,629

To functionally annotate the predicted genes, their protein sequences were compared against different protein databases including UnitProt (TrEMBL and SwissProt) and 2 insect proteomes (*A. pisum* and *Diaphorina citri*) using BLAST with an e-value cutoff of 1e–4. The protein sequences were also compared against the InterPro domain database [69]. Gene ontology (GO) annotation was performed with Blast2GO (Blast2GO, RRID:SCR_005828) [70]. Among the 17,629 predicted *R. maidis* genes, 75.6% had hits to proteins in the Swiss-Prot or TrEMBL database, 36.0% were annotated with GO terms, 75.2% contained InterPro domains, 76.3% shared detectable homology with *A. pisum* genes, and 47.9% shared detectable homology with *D. citri* genes. Among the 4,248 genes (24.1% of the total) having no significant homology with the *A. pisum* genome, 4,026 were annotated as “unknown protein.” Only 41 of these genes have GO annotations.

### Comparative genomics

**Figure 3:**
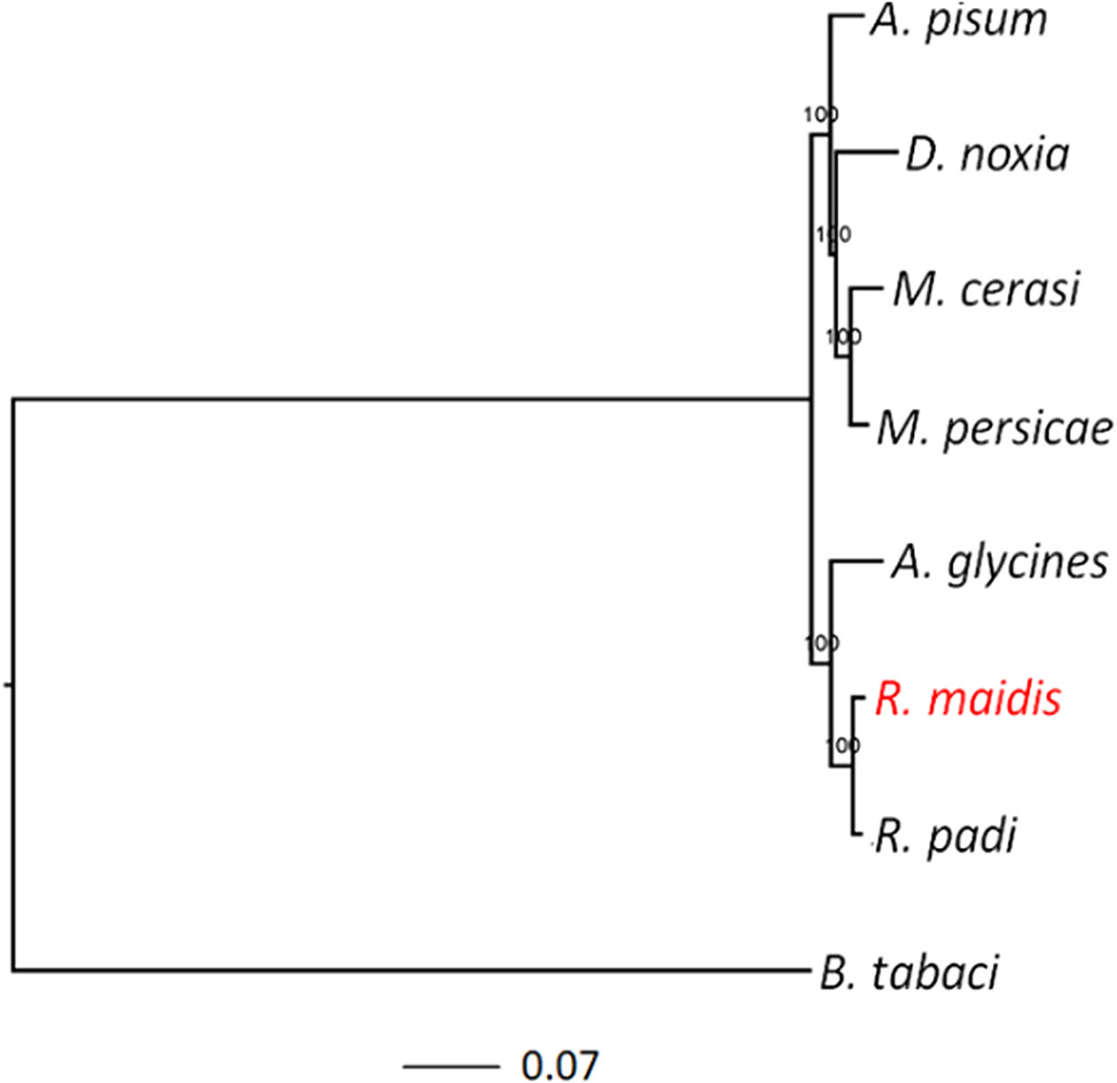
Phylogenetic relationships of *R. maidis* and 7 other arthropod species. *B. tabaci* was used as the outgroup taxon.

We compared the *R. maidis* genes with those of 6 other aphid species (*A. glycines, M. persicae, A. pisum, M. cerasi, R. padi*, and *D. noxia*), as well as the whitefly (*Bemisia tabaci*) [27–33, 71]. The proteome sequences of all 8 species were used to construct orthologous groups using OrthoMCL [72]. A total of 5,696 orthologous groups were shared by all 16 species, including 3,605 single-copy orthologous genes. Protein sequences of these single-copy genes were aligned with MUSCLE (MUSCLE, RRID:SCR_011812) [73], and positions in the alignment containing gaps in >20% of the sequences were removed by trimAl [74]. A phylogenetic tree was constructed using the maximum-likelihood method implemented in PhyML (PhyML, RRID:SCR_014629) [75], with the JTT model for amino acid substitutions and the aLRT method for branch support. *Bemisia tabaci* was used as the outgroup in the phylogenetic tree, which showed that *R. maidis* is close to *R. padi*, and separated from *A. pisum* and *M. persicae* (Fig. 3), consistent with a phylogeny that was derived using mtCOI [76].

### Identification of horizontal gene transfers

All of the *R. maidis* predicted gene models were compared against 6 protein databases derived from complete proteomes in UniProt (UniProt, RRID:SCR_002380), including those from bacteria, archaea, fungi, plants, metazoa (excluding proteins from other species in the Arthropoda), and other eukaryotes, using BLASTP (BLASTP, RRID:SCR_001010). The index of horizontal gene transfer (HGT), h, was calculated by subtracting the bitscore of the best metazoan match from that of the best non-metazoan match [77]. We required that these sequences be aligned better to the other 5 taxa than to the metazoan database, defining HGT candidates as those with h   ≥30 and a best non-metazoan hit bitscore  ≥100. The corresponding genome sequences of these candidates, as well as flanking aphid gene sequences at both ends, were manually checked using IGV [78] (Supplementary Figs S3-S22). If there is any region with substantially reduced coverage, between HGTs and their flanking aphid genes, then this HGT could be the result of incorrect assembly. Only HGTs in genomic regions with continuous read coverage were considered to be confirmed.

We phylogenetically validated all HGT candidates. Their protein sequences were compared against the protein databases of 6 taxa (archaea, bacteria, fungi, plants, metazoan, and other eukaryotes) using BLASTP. The top 5 hits from each taxon were extracted and aligned with the candidate HGT protein using ClustalW2 (ClustalW2, RRID:SCR_002909) [79]. Each alignment was trimmed to exclude regions where gaps were >20% of sequences. Phylogenetic trees were constructed using PhyML [75] using a JTT model with 100 bootstraps (Supplementary Data S2-S21). A horizontally transferred gene was considered valid if the gene was monophyletic within the bacteria, archaea, fungi, plants, or protozoa. This analysis identified 20 HGTs, including 2 of bacterial origin, 7 of fungal origin, 2 from protozoa, and 9 from algae (Table 4). The 2 bacterial genes were previously identified as horizontally transferred into *A. pisum* [80], and expression silencing of 1 of these genes, a bacteriocyte-expressed LD-carboxypeptidase A, was shown to reduce aphid performance [81]. A cluster of genes encoding multiple enzymes for carotenoid biosynthesis, which were horizontally transferred into the *A. pisum* genome from fungi [82], is also present in the *R. maidis* genome. Two *R. maidis* genes that cluster together with genes from trypanosomes and other protozoa have not been previously reported as horizontally transferred in aphids.

**Table 4:** Horizontally transferred genes in *R. maidis*

Gene ID	Function description	Possible origin
Rma07998	Peptidase U61; LD-carboxypeptidase A	Bacteria
Rma09603	Carbamoylphosphate synthase large subunit	Bacteria
Rma01752	Lycopene cyclase phytoene synthase	Fungi
Rma01753	Carotenoid desaturase	Fungi
Rma01754	Lycopene cyclase phytoene synthase	Fungi
Rma01756	Lycopene cyclase phytoene synthase	Fungi
Rma01758	Lycopene cyclase phytoene synthase	Fungi
Rma01759	Lycopene cyclase phytoene synthase	Fungi
Rma01760	Carotenoid desaturase	Fungi
Rma08772	Leucine rich repeat family protein	Protozoa
Rma11572	Antigenic protein, putative	Protozoa
Rma10344	Ankyrin repeat protein	Algae
Rma11418	Ankyrin repeat protein	Algae
Rma12243	Ankyrin repeat protein	Algae
Rma13322	Ankyrin repeat protein	Algae
Rma13584	Ankyrin repeat protein	Algae
Rma14036	Ankyrin repeat protein	Algae
Rma15269	Ankyrin repeat protein	Algae
Rma16213	Ankyrin repeat protein	Algae
Rma16838	Ankyrin repeat protein	Algae

**Figure 4:**
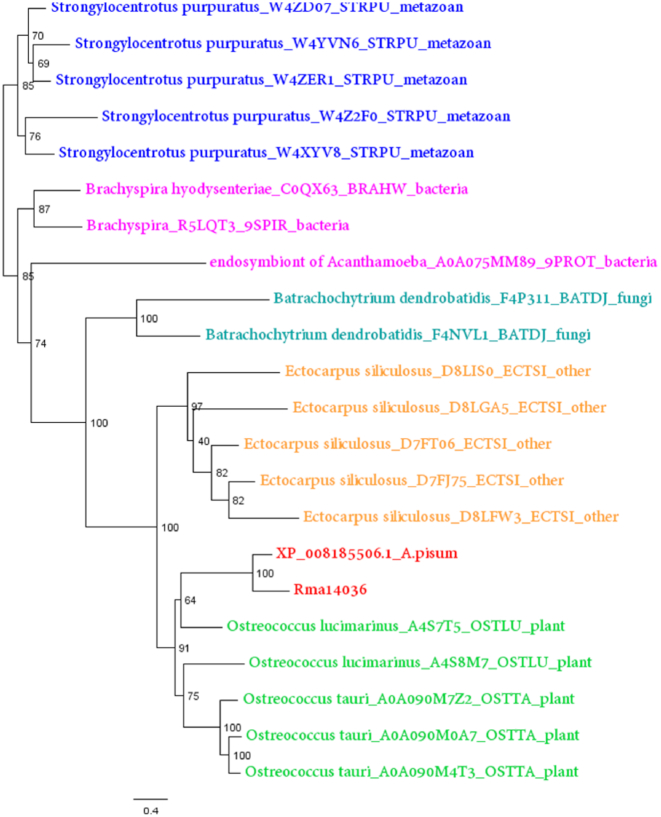
A family of aphid proteins (examples of Rma14036 from *R. maidis* and XP_008185506.1 from *A. pisum* are shown in red) cluster most closely with proteins from *Ostreococcus* algae.

It is perhaps surprising that 9 genes encoding proteins with ankyrin repeat domains show the highest similarity to genes from unicellular algae in the genus *Ostreococcus* and cluster with genes from this species in phylogenetic trees (Fig. 4; Supplementary Data S11–S21). However, this does not necessarily mean that these genes were transferred from *Ostreococcus*, a type of picoplankton, but only that these are the most similar sequences available in UniProt. Three lines of evidence confirm that these are actual genes encoded in aphids: (i) the sequences are contiguous with other aphid genes in contigs assembled from PacBio long reads (Supplementary Figs S13–S22), (ii) RNA-Seq shows that the genes are transcribed in *R. maidis* (Supplementary Fig. S23), and (iii) there are homologs of these genes in the other 6 published aphid genomes (e.g., XP_008185506.1 from *A. pisum*; Fig. 4).

### Detoxification and insecticide resistance

Cytochrome P450s, glutathione *S-*transferases (GSTs), carboxylesterases, uridine diphosphate (UDP)-glucosyltransferases, and ABC transporters function in the avoidance and/or detoxification of plant defensive metabolites [83, 84], and insecticide resistance [85, 86]. We identified such detoxification-related genes in *R. maidis* on the basis of protein domains that were predicted through InterProScan (InterProScan, RRID:SCR_005829) [87]. Cytochrome P450 genes were identified if their protein sequences contained the cytochrome P450 domain (InterPro ID: IPR001128). Genes with protein sequences containing the GST N-terminal and/or C-terminal domains (InterPro ID: IPR004045, IPR004046) were identified as GSTs. Carboxylesterases were identified on the basis of protein sequences that contained the carboxylesterase domain (InterPro domain ID: IPR002018) [88]. UDP-glucuronosyltransferases were identified if their protein sequences contained a UDP-glucuronosyl/UDP-glucosyltransferase domain (InterPro domain ID: IPR002213). ABC transporters were identified from the genome if their protein sequences contained an ABC transporter–like domain (InterPro ID: IPR003439). Using the same approach, genes from these families were also identified in the other 6 aphid genomes (*A. glycines, M. persicae, A. pisum, M. cerasi, R. padi*, and *D. noxia*). The number of predicted detoxification genes in *R. maidis* is the lowest among the 7 species that were examined (Table 5; Supplementary Data S30), consistent with *R. maidis* being a specialist monocot herbivore that may require a smaller repertoire of detoxification enzymes. Although the detoxification gene count in *A. pisum* was high, the mean lengths of the protein sequences were shorter than those in *R. maidis, A. glycines*, and *M. persicae* (Supplementary Fig. S24), suggesting that these genes could be incomplete or pseudogenes in *A. pisum*, possibly due to a lower quality genome assembly.

**Table 5:** Numbers of predicted detoxification genes in 7 aphid species

	*R. maidis*	*A. glycines*	*M. persicae*	*A. pisum*	*M. cerasi*	*R. padi*	*D. noxia*
Cytochrome P450s	59	61	67	82	74	67	60
Glutathione S-transferases	10	12	13	36	12	11	11
Carboxylesterases	23	31	37	48	36	34	32
UDP-glucuronosyltransferases	43	47	57	72	48	55	43
ABC transporters	68	74	67	126	68	71	63
Total	203	225	241	364	238	238	209

## Conclusion

As the currently most complete aphid genome, our *R. maidis* assembly will provide a valuable resource for comparisons with other species and the investigation of aphid genome evolution. Research on the ecological interactions of *R. maidis*, including host plant choices, detoxification of secondary metabolites, and gene expression responses, will be facilitated by the *R. maidis* genome sequence. Practical applications in agriculture may include the identification of virus transmission mechanisms and new targets for chemical pest control.

## Availability of supporting data and materials

This *R. maidis* Whole Genome Shotgun project has been deposited at DDBJ/ENA/GenBank under accession No. QORX00000000. The version described in this article is version QORX02000000. The *R. maidis* mitochondrial genome has been deposited in GenBank under accession No. MK368778. The *Buchnera aphidicola* Rm genome has been deposited in GenBank under accession No. CP032759. Raw genome and RNA-Seq sequences have been deposited in the NCBI Sequence Read Archive under accession No. SRP164762. Genome sequence and annotation data are also available via the *GigaScience* database (GigaDB) [89].

## Additional files

**Figure S1**. Hi-C contact map of the *R. maidis* genome

**Figure S2**. Circular view of the genome of the *Rhopalosiphum maidis* endosymbiont, *Buchnera aphidicola* (A), and its plasmids pLeu (B) and pTrp (C)

**Figure S3-S22**. PacBio read alignments around the HGT genes

**Figure S23**. RNA-Seq reads alignments around the Rma14036 HGT gene

**Figure S24**. Length distribution of protein sequences of detoxification gene families in 7 aphid species

**Data S1**. Summary of PacBio long reads and Illumina short reads

**Data S2-S21**. Phylogenetic tree files for testing candidate HGT

**Data S22**. Predicted detoxification genes in *R. maidis*

## Abbreviations

ATP: adenosine triphosphate; bp: base pairs; BUSCO: Benchmarking Universal Single-Copy Orthologs; EDTA: ethylenediaminetetraacetic acid; Gb: gigabase; GO: gene ontology; GST: glutathione S-transferase; HGT: horizontal gene transfer; Mb: megabase; MITE: miniature inverted-repeat transposable elements; NCBI: National Center for Biotechnology Information; PacBio: Pacific Biosciences; RNA-Seq: RNA sequencing; SMRT: single-molecule real-time sequencing; SPB: sample purification beads; UDP: uridine diphosphate.

## Competing interests

The authors declare that they have no competing interests.

## Funding

This research was sponsored by the Defense Advanced Research Projects Agency (DARPA) and was accomplished under cooperative agreement No. HR0011–17-2–0053. The views and conclusions contained in this document are those of the authors and should not be interpreted as representing the official policies, either expressed or implied, of DARPA or the US Government. The US Government is authorized to reproduce and distribute reprints for Government purposes notwithstanding any copyright notation hereon.

## Authors' contributions

G.J. and Z.F. conceived of the research; S.S., M.B., and A.R. raised aphids and isolated nucleic acids; W.C. conducted data analysis; and W.C. and G.J. wrote the manuscript.

## Supplementary Material

GIGA-D-18-00405_Original_Submission.pdf

GIGA-D-18-00405_Revision_1.pdf

GIGA-D-18-00405_Revision_2.pdf

GIGA-D-18-00405_Revision_3.pdf

Response_to_Reviewer_Comments_Original_Submission.pdf

Response_to_Reviewer_Comments_Revision_1.pdf

Response_to_Reviewer_Comments_Revision_2.pdf

Reviewer_1_Report_Original_Submission -- Andrew Michel 11/15/2018 Reviewed

Reviewer_2_Report_Original_Submission -- Anna-Maria Botha 11/21/2018 Reviewed

Reviewer_3_Report_Original_Submission -- Shuji Shigenobu 12/6/2018 Reviewed

Reviewer_3_Report_Revision_1 -- Shuji Shigenobu 2/4/2019 Reviewed

Supplemental Files
