# Supplementary material for: Genome sequence of the corn leaf aphid (Rhopalosiphum maidis Fitch)
Source: Gigascience. 2019 Apr 6;8(4):giz033. doi: 10.1093/gigascience/giz033 (PMC6451198; doi:10.1093/gigascience/giz033)
Supplement: GIGA-D-18-00405_Original_Submission.pdf [file giz033_giga-d-18-00405_original_submission.pdf]

GigaScience

Genome sequence of the corn leaf aphid (Rhopalosiphum maidis Fitch)

--Manuscript Draft--

**Manuscript Number:**	GIGA-D-18-00405
**Full Title:**	Genome sequence of the corn leaf aphid (Rhopalosiphum maidis Fitch)
**Article Type:**	Data Note
**Funding Information:**	Defense Advanced Research Projects Agency (HR0011-17-2-0053) — Dr. Georg Jander
**Abstract:**	Background: The corn leaf aphid (Rhopalosiphum maidis Fitch) is the most economically damaging aphid pest on maize (Zea mays), one of the world's most important grain crops. In addition to causing direct damage due to the removal of photoassimilates, R. maidis transmits several destructive maize viruses, including Maize yellow dwarf virus, Barley yellow dwarf virus, Sugarcane mosaic virus, and Cucumber mosaic virus.<br>Findings: A 326-Mb genome assembly of BTI-1, a parthenogenetically reproducing R. maidis clone, was generated with a combination of PacBio (208-fold coverage) and Illumina sequencing (80-fold coverage), which contains a total of 689 contigs with an N50 size of 9.0 Mb. The contigs were further clustered into four scaffolds using the Hi-C interaction maps, consistent with the commonly observed 2n = 8 karyotype of R. maidis. Most of the assembled contigs (473 spanning 321 Mb) were successfully orientated in the four scaffolds. The R. maidis genome assembly captured the full length of 95.8% of the core eukaryotic genes, suggesting that it is highly complete. Repetitive sequences accounted for 21.2% of the assembly, and a total of 17,647 protein-coding genes were predicted in the R. maidis genome with integrated evidence from ab initio and homology-based gene predictions and transcriptome sequences generated with both PacBio and Illumina. An analysis of likely horizontally transferred genes identified two from bacteria, seven from fungi, two from protozoa, and nine from algae.<br>Conclusions: A high-quality R. maidis genome was assembled at the chromosome level. This genome sequence will enable further research related to ecological interactions, virus transmission, pesticide resistance, and other aspects of R. maidis biology. It also serves as a valuable resource for comparative investigation of other aphid species.
**Corresponding Author:**	Georg Jander<br>Boyce Thompson Institute for Plant Research<br>Ithaca, NY UNITED STATES
**Corresponding Author Secondary Information:**	
**Corresponding Author's Institution:**	Boyce Thompson Institute for Plant Research
**Corresponding Author's Secondary Institution:**	
**First Author:**	Wenbo Chen
**First Author Secondary Information:**	
**Order of Authors:**	Wenbo Chen
	Sara Shakir
	Mahdiyeh Bigham
	Zhangjun Fei
	Georg Jander
**Order of Authors Secondary Information:**	
**Additional Information:**	

Powered by Editorial Manager® and ProduXion Manager® from Aries Systems Corporation

Question	Response
Are you submitting this manuscript to a special series or article collection?	No
**Experimental design and statistics**<br><br>Full details of the experimental design and statistical methods used should be given in the Methods section, as detailed in our Minimum Standards Reporting Checklist. Information essential to interpreting the data presented should be made available in the figure legends.<br><br>Have you included all the information requested in your manuscript?	Yes
**Resources**<br><br>A description of all resources used, including antibodies, cell lines, animals and software tools, with enough information to allow them to be uniquely identified, should be included in the Methods section. Authors are strongly encouraged to cite Research Resource Identifiers (RRIDs) for antibodies, model organisms and tools, where possible.<br><br>Have you included the information requested as detailed in our Minimum Standards Reporting Checklist?	Yes
**Availability of data and materials**<br><br>All datasets and code on which the conclusions of the paper rely must be either included in your submission or deposited in publicly available repositories (where available and ethically appropriate), referencing such data using a unique identifier in the references and in the "Availability of Data and Materials" section of your manuscript.	Yes

Powered by Editorial Manager® and ProduXion Manager® from Aries Systems Corporation

Have you have met the above requirement as detailed in our Minimum Standards Reporting Checklist?	

Manuscript
Click here to download Manuscript R maidis genome MS - 101218.docx

Genome sequence of the corn leaf aphid (*Rhopalosiphum maidis* Fitch)

Wenbo Chen[1], Sara Shakir[1,3], Mahdiyeh Bigham[1], Zhangjun Fei[1,2], and Georg Jander[1,*]

[1]Boyce Thompson Institute, 533 Tower Road, Ithaca, NY 14853, USA

[2]US Department of Agriculture-Agricultural Research Service, Robert W. Holley Center for Agriculture and Health, Ithaca, NY 14853, USA

[3]Present address: Plant Genetics Lab, Gembloux Agro-Bio Tech, University of Liège, Gembloux, Belgium

*To whom correspondence should be addressed:

Georg Jander

Boyce Thompson Institute

533 Tower Road

Ithaca NY 14853

Phone: (1) 607-254-1365

Email: gj32@cornell.edu

ORCID

Wenbo Chen: 0000-0003-4771-9437

Sara Shakir: 0000-0002-8830-5989.

Mahdieh Bigham: 0000-0003-1396-4338

Zhangjun Fei: 0000-0001-9684-1450

Georg Jander: 0000-0002-9675-934X

Abstract

Background: The corn leaf aphid (*Rhopalosiphum maidis* Fitch) is the most economically damaging aphid pest on maize (*Zea mays*), one of the world's most important grain crops. In addition to causing direct damage due to the removal of photoassimilates, *R. maidis* transmits several destructive maize viruses, including *Maize yellow dwarf virus*, *Barley yellow dwarf virus*, *Sugarcane mosaic virus*, and *Cucumber mosaic virus.*

Findings: A 326-Mb genome assembly of BTI-1, a parthenogenetically reproducing *R. maidis* clone, was generated with a combination of PacBio (208-fold coverage) and Illumina sequencing (80-fold coverage), which contains a total of 689 contigs with an N50 size of 9.0 Mb. The contigs were further clustered into four scaffolds using the Hi-C interaction maps, consistent with the commonly observed 2n = 8 karyotype of *R. maidis.* Most of the assembled contigs (473 spanning 321 Mb) were successfully orientated in the four scaffolds. The *R. maidis* genome assembly captured the full length of 95.8% of the core eukaryotic genes, suggesting that it is highly complete. Repetitive sequences accounted for 21.2% of the assembly, and a total of 17,647 protein-coding genes were predicted in the *R. maidis* genome with integrated evidence from *ab initio* and homology-based gene predictions and transcriptome sequences generated with both PacBio and Illumina. An analysis of likely horizontally transferred genes identified two from bacteria, seven from fungi, two from protozoa, and nine from algae.

Conclusions: A high-quality *R. maidis* genome was assembled at the chromosome level. This genome sequence will enable further research related to ecological interactions, virus transmission, pesticide resistance, and other aspects of *R. maidis* biology. It also serves as a valuable resource for comparative investigation of other aphid species.

Keywords: corn leaf aphid, genome, annotation, *Rhopalosiphum maidis*

Data Description

Introduction

Maize (*Zea mays*), the world's most productive grain crop, is susceptible to more than 90 species of herbivorous insects [1-3]. Among aphids that feed on maize, the corn leaf aphid (*Rhopalosiphum maidis* Fitch) is the most commonly encountered, particularly in tropical and warmer temperate areas [4]. Relative to other maize-feeding aphids (*Rhopalosiphum padi*, *Schizaphis graminum*, *Sitobion avenae*, and *Metopolophium dirhodum*), *R. maidis* exhibits a greater tolerance of benzoxazinoids, the most abundant class of maize defensive metabolites [5]. However, the mechanism of aphid resistance to these plant toxins is not known, and natural variation in benzoxazinoid content among maize inbred lines nevertheless influences growth and reproduction of *R. maidis* [6, 7].

Damage caused to maize by *R. maidis* takes several forms, and the resulting yield losses can be quite variable from year to year. Growth and yield are reduced through the removal of photosynthates by large numbers of aphids [8]. On flowering-stage maize, aphids tend to congregate on the tassels, where large amounts of honeydew can prevent the release of pollen from the anthers, thereby reducing seed set by up to 90% [9, 10]. Additional damage comes from the fact that *R. maidis* transmits several important maize viruses, including *Maize yellow dwarf virus*, *Barley yellow dwarf virus*, *Sugarcane mosaic virus*, and *Cucumber mosaic virus* [11-15].

In addition to feeding on maize, *R. maidis* also infests a variety of other monocot species, including barley, oat, rice, rye, sorghum, sugarcane, and wheat [4]. In one study, barley was reported as the most suitable grain crop host for *R. maidis* [16]. However, as in the case of maize, there is also considerable within-species variation for *R. maidis* resistance in barley [17].

The origin of *R. maidis* is likely in Asia, and it has been subsequently introduced in most grain-growing areas of the world. In almost all parts of its range, *R. maidis* is anholocyclic. However, sexual reproduction has been reported in Pakistan and Korea, with *Prunus* ssp. as the primary host [18, 19]. In populations in Japan and Kenya, males but not sexually reproducing females have been found [20, 21]. Consistent with the sometimes permanently parthenogenetic life cycle of *R. maidis,* there is within-species variation in the chromosome numbers. Karyotypes of 2n = 8, 9, and 10 have been reported. There also is evidence of host specificity among the

karyotypes. Whereas *R. maidis* strains on maize tend to have 2n = 8, those on barley generally have 2n = 10 [22, 23].

Here we report the genome sequence of *R. maidis* isolate BTI-1. Comparisons to six previously published aphid genomes [24-31] showed an improved assembly, with most of the sequences assembled into four scaffolds, consistent with the 2n = 8 karyotype of *R. maidis.* Analysis of the assembled *R. maidis* genome identified horizontally transferred genes, repetitive elements, and likely xenobiotic detoxification enzymes.

Sampling and genome sequencing

Insect Colony. BTI-1, a corn leaf aphid (*R. maidis*) isolate, which was originally collected from maize (*Z. mays*) in New York State, was obtained from Stewart Gray (USDA Plant Soil and Nutrition Laboratory, Ithaca, NY). An isogenic colony was started from a single parthenogenetic female *R. maidis* and was maintained on barley (*Hordeum vulgare*) prior to the collection of insects for genome and transcriptome sequencing.

Genomic DNA was prepared from 100-200 mg of fresh *R. maidis* tissue using a previously described protocol [32]. Briefly, mixed-instar whole aphids were ground in liquid nitrogen and incubated at 65°C in microprep buffer made up of DNA extraction buffer (0.35M sorbitol, 0.1M Tris-base, pH7.5, 5mM ethylenediaminetetraacetic acid), nuclei lysis buffer (0.2M Tris-base, pH 7.5, 0.05M ethylenediaminetetraacetic acid, 2M NaCl, 2% cetyl trimethylammonium bromide), 5% sarkosyl and 0.5% sodium bisulfite for 30 min. This solution was then treated with chloroform:isoamyl alcohol (24:1) and centrifuged for 10 min at 14,000 *g*. The supernatant was treated with RNase A and DNA was pelleted by centrifugation at 4°C at 14,000 *g* for 10 min. DNA pellet was washed with 100% isopropanol and then with 70% ethanol and dissolved in 50 µl of nuclease free water. Around 50 µg of high molecular weight DNA was prepared for PacBio library construction and sequencing using SMRT Cell template preparation kits (Pacific Biosciences), and sequencing was conducted at the Icahn Institute and Department of Genetics and Genomic Sciences, Icahn School of Medicine at Mount Sinai. A total of 16 SMRT Cells were run on the PacBio Sequel platform, yielding 70 Gb raw sequence data (**Supplemental Table S1**), representing a 208-fold coverage of the *R. maidis* genome, which was estimated to be 338 Mb using the kmer approach ([33]; **Figure 1**). For short-read sequencing, one paired-end library was

constructed using the Illumina TruSeq DNA sample preparation kit following the manufacturer's instructions, and sequenced on an Illumina HiSeq 2500 system, which yielded about 75 Gb of raw sequence data (**Supplemental Table S2**). Raw Illumina reads were processed to remove duplicated read pairs, which were defined as having identical bases in the first 100 bp of both left and right reads, and only one read pair from each duplicated sequence was kept. Illumina adapters and low-quality sequences were removed from the reads using Trimmomatic [34]. The kmer depth distribution of the cleaned high-quality sequences displayed a single peak (**Supplemental Figure S1**), indicating that the sequenced sample has a low level of heterozygosity.

Transcriptome sequencing

Transcriptome sequencing (Illumina strand-specific RNA-Seq and PacBio Iso-Seq) was conducted to aid gene prediction. Total RNA was extracted using the SV Total RNA isolation kit (Promega: Catalog number: Z3100). Briefly, cells were lysed by grinding 100-120 mg of insect tissue in liquid nitrogen, followed by incubation at 70°C in RNA lysis buffer (4M guanidine thiocyanate (GTC), 0.01M Tris, pH 7.5, 0.97% β-mercaptoethanol) for 3 min. This solution was then centrifuged for 10 min at 14,000 *g* and the supernatant was passed through a spin column provided with the kit, followed by DNase treatment. RNA was washed with RNA wash solution (60 mM potassium acetate, 10 mM Tris-HCl (pH 7.5), 60% ethanol) and dissolved in 50 µl of nuclease-free water. Strand-specific RNA-Seq libraries were constructed using a previously described protocol [35] and sequenced at Biotechnology Resource Center of Cornell University on an Illumina HiSeq 2500 sequencing system. More than 188 million paired-end reads with lengths of 151 bp were obtained (**Supplemental Table S2**). Raw reads were processed by trimming adaptor and low-quality sequences using Trimmomatic [34]. The cleaned reads were aligned to the assembled *R. maidis* genome using HISAT2 [36], followed by reference-guided assembly using StringTie [37]. The assembled transcripts were used to improve protein-coding gene predictions in the *R. maidis* genome.

For Iso-Seq, 20 µg RNA, isolated from 100-120 mg of fresh *R. maidis* tissue using the SV Total RNA isolation kit (Promega) with the method described above, was shipped to Duke Center for Genomic and Computational Biology for PacBio large-insert (15-20kb) library construction and sequencing using standard SMRTbell template preparation kits. One SMRT cell was run on the

PacBio Sequel platform, yielding ~10 Gb raw sequence data (**Supplemental Table S1**). The PacBio raw reads were processed using IsoSeq3 (https://github.com/PacificBiosciences/IsoSeq3). Briefly, one representative Circular Consensus Sequence (CCS) was generated for each zero-mode waveguide (ZMW). Only ZMWs with at least one full pass, meaning that each primer has been seen at least once, were used for the subsequent analysis. The CCSs were processed to remove the 5' and 3' primers, trim off polyA tails and remove artificial concatemers to create full-length, non-concatemer (FLNC) reads. The FLNC reads were then clustered together. The final polishing step created a consensus sequence for each clustered transcript. A total of 21,114 high quality transcripts were generated, and were used to support protein-coding gene predictions in the *R. maidis* genome.

Hi-C library construction and sequencing

For Hi-C sequencing, 200 mg of *R. maidis* tissue was used for chromatin isolation and library preparation using the animal Hi-C kit from Phase Genomics (https://phasegenomics.com*)*. Hi-C Libraries were sequenced at the Biotechnology Resource Center, Cornell University, using the NextSeq500 platform (Illumina) to obtain 76-nt paired-end reads. Raw reads were processed by trimming adaptor and low-quality sequences using Trimmomatic [34]. The cleaned Hi-C reads were aligned to the assembled contigs using BWA-aln [38], and the optimal placement of each read pair was determined by BWA-sampe [38]. Reads that did not map within 500 bp of a restriction enzyme site were removed using the PreprocessSAMs.pl script in LACHSIS [39]. Finally, only reads with mapping quality greater than 30 were used for scaffolding by LACHSIS [39].

Genome assembly

The PacBio long reads were corrected and assembled with the Canu assembler [40] (version 1.6). The resulting contigs were polished by aligning the raw PacBio reads to the assembly, and correcting the sequencing errors using Arrow (https://github.com/PacificBiosciences/GenomicConsensus). To further improve the assembly, another round of polishing was performed by aligning the Illumina short reads to the assembly and correcting the sequencing errors using Pilon [41]. The assembled contigs were then compared

against the NCBI non-redundant nucleotide (nt) database using BLASTN with an e-value cutoff of 1e-5. Contigs with over 90% of their lengths similar to only bacterial or viral sequences were considered to be contaminants and were discarded. The final contigs were clustered and ordered into chromosomes by Hi-C reads using LACHSIS [39] with default parameters.

The assembled *R. maidis* genome had a total length of 326.0 Mb and consisted of 689 contigs with an N50 length of 9.0 Mb. Thus, this is a much-improved genome assembly compared to the six previously published aphid genomes (**Table 1**). A total of 602 contigs spanning 323.4 Mb (99.2% of the assembly) were clustered into four groups, which was consistent with the commonly observed 2n = 8 karyotype of *R. maidis* [22]. Of the clustered contigs, 473 spanning 320.6 Mb (98.4% of the assembly) were successfully orientated (**Figure 1, Supplemental Figure 2**). To evaluate the completeness of the *R. maidis* genome assembly, the Illumina paired-end library were aligned to the assembly, allowing up to three mismatches using BWA-MEM [38]. With this approach, 94.9% of the Illumina reads could be mapped back to the assembly, indicating that most of the reads were successfully assembled into the genome. RNA-Seq reads also were aligned to the genome assembly using HISAT2 [36], resulting a mapping ratio of 94.5% (**Supplemental Table S2**). Furthermore, the completeness of the genome assembly, as evaluated by BUSCO (v 3.0.2 [42], showed that 95.8% of the core eukaryotic genes were at least partially captured by the genome assembly and 94.5% were completely captured. Taken together, our evaluations indicated an overall high quality of the assembled *R. maidis* genome.

Endosymbiont genomes

The genome sequence of the *Buchnera aphidicola* endosymbiont was separated from the *R. maidis* host genome sequences by aligning the initial assembly to the *Buchnera* reference genome (GeneBank ID: NC_002528.1). One single contig was extracted and polished using both PacBio long reads and Illumina short reads, as described above. Genome annotation was performed using prokka [43]. The assembled *Buchnera*Rm genome had a length of 642,929 bp (**Supplemental Figure S3**), with a total of 602 predicted protein-coding genes. The two *Buchnera* plasmids, pLeu and pTrp, were also sequenced and assembled, with lengths of 7,852 bp and 3,674 bp, respectively (**Supplemental Figure S3**).

To identify secondary bacterial symbionts in *R. maidis*, raw assembled contigs were compared against the reference sequences of previously identified secondary bacterial symbionts of aphids, including *Hamiltonella defensa*, *Regiella insecticola*, *Serratia symbiotica*, *Rickettsia*, *Spiroplasma*, X-type, *Arsenophonus,* and *Wolbachia* [44], using BLAST. No hits were found, suggesting that these secondary bacterial symbionts are not hosted by the sequenced *R. maidis* strain.

Annotation of repetitive elements

We first identified MITE (miniature inverted-repeat transposable elements) from the assembled *R. maidis* genome using MITE-Hunter [45], and then generated a *de novo* repeat library by scanning the assembled genome using RepeatModeler (http://www.repeatmasker.org/RepeatModeler), which integrates results from RECON [46], TRF [47], and RepeatScout [48] and classifies repeats with the RepBase library [49]. RepeatModeler identified a total of 546 repeats. We subsequently compared these repeat sequences against the NCBI non-redundant (nr) protein database using BLAST with an e-value cutoff of 1e-5, and those having hits to known protein sequences were excluded. Finally, we identified repeat sequences by scanning the assembled *R. maidis* genome using the *de novo* repeat library with RepeatMasker (http://www.repeatmasker.org/) and the RepeatRunner subroutine (http://www.yandell-lab.org/software/repeatrunner.html) in the MAKER annotation pipeline [50]. A total of 21.2% of the assembled *R. maidis* genome was annotated as repeat elements (**Table 2**). The most predominant repeat elements were unknown repeats and MITEs, which occupied 5.6% and 4.4% of the genome respectively.

Gene prediction

Protein-coding genes were predicted from the genome assembly of *R. maidis* using the automated pipeline MAKER [50]. MAKER integrates the results from *ab initio* gene predictions with experimental gene evidence to produce final consensus gene set. The evidence that was used included complete aphid coding sequences collected from NCBI, transcripts assembled from our strand-specific RNA-Seq data, high quality transcript sequences from Iso-Seq, completed proteomes of *Acyrthosiphon pisum, Aphis glycines, Diuraphis noxia, Myzus cerasi, Myzus persicae,* and *Rhopalosiphum padi*, and proteins from the Swiss-Prot database. All of these sequences were aligned to the *R. maidis* genome using Spaln [51]. MAKER was used to run a

battery of trained gene predictors, including Augustus [52], BRAKER [53] and GeneMark-ET [54], and then integrated the experimental gene evidence to produce evidence-based predictions. Altogether, 17,647 protein-coding genes were predicted in the *R. maidis* genome. The gene count of *R. maidis* is close to those in *Ap. Glycines* and *M. persicae*, while fewer than those in *Ac. Pisum*, *M. cerasi*, *R. padi* and *D. noxia*, which possess larger genome sizes (**Table 1**). The mean lengths of coding sequences were similar, with the exception of *M. cerasi* and *D. noxia*.

To functionally annotate the predicted genes, their protein sequences were compared against different protein databases including UnitProt (TrEMBL and SwissProt) and two insect proteomes (pea aphid and psyllid) using BLAST with an e-value cutoff of 1e-4. The protein sequences were also compared against the InterPro domain database [55]. GO annotation was performed with Blast2GO [56]. Among the 17,647 predicted *R. maidis* genes, 75.6% had hits to proteins in the Swiss-Prot or TrEMBL database, 36.0% were annotated with GO terms, 75.2% contained InterPro domains, 76.3% shared detectable homology with *A. pisum* genes, and 47.9% shared detectable homology with *Diaphorina citri* genes.

Comparative genomics

We compared the *R. maidis* genes with those of six other aphid species (*Ap. glycines, M. persicae, Ac. pisum, M. cerasi, R. padi,* and *D. noxia*), as well as the whitefly (*Bemisia tabaci*) [24-31]. The proteome sequences of all eight species were used to construct orthologous groups using OrthoMCL [57]. A total of 5,696 orthologous groups were shared by all 16 species, including 3,605 single-copy orthologous genes. These single-copy genes were used to reconstruct their phylogenetic relationships. Briefly, protein sequences of the single-copy genes were aligned with MUSCLE [58], and positions in the alignment containing gaps in more than 20% of the sequences were removed by trimAl [59]. A phylogenetic tree was then constructed using the Maximum-Likelihood method implemented in PhyML [60], with the JTT model for amino acid substitutions and the aLRT method for branch support. *B. tabaci* was used as the outgroup in the phylogenetic tree, which showed that *R. maidis* is close to *R. padi*, and separated from *A. pisum* and *M. persicae* (**Figure 2**), consistent with a phylogeny that was derived using mtCOI [61].

Identification of horizontal gene transfers

All of the *R. maidis* predicted gene models were compared against six protein databases derived from complete proteomes in UniProt, including those from bacteria, archaea, fungi, plants, metazoa (excluding proteins from other species in the Arthropoda), and other eukaryotes, using BLASTP. The index of horizontal gene transfer (HGT), h, was calculated by subtracting the bitscore of the best metazoan match from that of the best non-metazoan match [61].We required that these sequences were aligned better to the other five taxa than to the metazoan database, defining HGT candidates as those with $h \geq 30$ and a best non-metazoan hit bitscore ≥ 100. The corresponding genome sequences of these candidates as well as 1000-bp flanking sequences at both ends were manually checked for potential genome assembly errors, and none were found.

We phylogenetically validated all HGT candidates. Their protein sequences were compared against the protein databases of six taxa (archaea, bacteria, fungi, plants, metazoan, and other eukaryotes) using BLASTP. The top five hits from each taxon were extracted, and aligned with the candidate HGT protein using ClustalW2 [63]. Each alignment was trimmed to exclude regions where gaps were more than 20% of sequences. Phylogenetic trees were constructed using PhyML [60] using a JTT model with 100 bootstraps. A horizontally transferred gene was considered valid if the gene was monophyletic within the bacteria, archaea, fungi, plants, or protozoa. This analysis identified 20 HGTs, including two of bacterial origin, seven of fungal origin, two from protozoa, and nine from algae (**Table 3**). With the exception of the two protozoa-derived genes, expression of all of the HGTs was detected in the RNAseq data set.

The two bacterial genes were previously identified as horizontally transferred into *Ac. pisum* [64], and expression silencing of one of these genes, a bacteriocyte-expressed LD-carboxypeptidase A, was shown to reduce aphid performance [65]. A cluster of genes encoding multiple enzymes for carotenoid biosynthesis, which were horizontally transferred into the *Ac. pisum* genome from fungi [66], is also present in the *R. maidis* genome. Two *R. maidis* genes that cluster together with genes from trypanosomes and other protozoa have not been previously reported as horizontally transferred in aphids. Finally, nine genes encoding proteins containing ankyrin repeat domains show highest similarity to genes from unicellular algae in the genus *Ostreococcus.*

Detoxification and insecticide resistance

Cytochrome P450s, glutathione *S*-transferases (GSTs), carboxylesterases, UDP-glucosyltransferases (UGTs), and ABC transporters function in the avoidance and/or detoxification of plant defensive metabolites [67, 68], and insecticide resistance [69, 70]. We identified such detoxification-related genes in *R. maidis* based on protein domains that were predicted through InterProScan [71]. Cytochrome P450 genes were identified if their protein sequences contained the cytochrome P450 domain (InterPro ID: IPR001128). Genes with protein sequences containing the GST N-terminal and/or C-terminal domains (InterPro ID: IPR004045, IPR004046) were identified as GSTs. Carboxylesterases were identified on the basis of protein sequences that contained the carboxylesterase domain (InterPro domain ID: IPR002018) [72]. UDP-glucuronosyltransferases were identified if their protein sequences contained a UDP-glucuronosyl/UDP-glucosyltransferase domain (InterPro domain ID: IPR002213). ABC transporters were identified from the genome if their protein sequences contained an ABC transporter-like domain (InterPro ID: IPR003439). Using the same approach, genes from these families were also identified in the other six aphid genomes (*Ap. glycines*, *M. persicae*, *Ac. pisum*, *M. cerasi*, *R. padi*, *D. noxia*). The number of predicted detoxification genes in *R. maidis* is the lowest among the seven species that were examined (**Table 4** and **Supplemental Table S3**), consistent with *R. maidis* being a specialist monocot herbivore that may require a smaller repertoire of detoxification enzymes. Although the detoxification gene count in *Ac. pisum* was high, the average lengths of the protein sequences were shorter than those in *R. maidis*, *Ap. glycines*, and *M. persicae* (**Supplemental Figure S4**), suggesting that these genes could be incomplete or pseudogenes in *Ac. Pisum*, possibly due to a lower-quality genome assembly.

Conclusion

As the currently most complete aphid genome, our *R. maidis* assembly will provide a valuable resource for comparisons with other species and the investigation of aphid genome evolution. Research on the ecological interactions of *R. maidis*, including host plant choices, detoxification of secondary metabolites, and gene expression responses, will be facilitated by the *R, maidis* genome sequence. Practical applications in agriculture may include the identification of virus transmission mechanisms and new targets for chemical pest control.

Availability of supporting data

This Whole Genome Shotgun project has been deposited at DDBJ/ENA/GenBank under the accession QORX00000000. The version described in this paper is version QORX01000000. The *Buchnera aphidicola* Rm genome has been deposited in GenBank under accession CP032759. Raw genome and RNA-Seq sequences have been deposited in the NCBI Short Sequence Archive (SRA) under accession SRP164762.

Additional Files

Figure S1. Kmer (K=31) distribution of Illumina genome sequencing reads of *R. maidis*. The total count of kmers was 11,495,021,417, and the peak of kmer depth was 34. The genome size of *R. maidis* was calculated by dividing the total kmer count by the peak depth, which was approximately 338 Mb.

.

Figure S2. Hi-C contact map of the *R. maidis* genome

.

Figure S3. Circular view of the genome of the *Rhopalosiphum maidis* endosymbiont, *Buchnera aphidicola* (A) and its plasmids pLeu (B) and pTrp (C).

Figure S4. Length distribution of protein sequences of detoxification gene families in seven aphid species.

Table S1. Summary of PacBio long reads

Table S2. Summary of Illumina short reads

Table S3. Detoxification genes in *Rhopalosiphum maidis*

Abbreviations

CCS: circular consensus sequence

ZMW: zero-mode waveguide

FLNC: full-length, non-concatemer reads

nt: nucleotide

HGT: horizontal gene transfer

GST: glutathione S-transferase

CCE: carboxylesterase

UGT: UDP-glucuronosyltransferases

Author contributions

GJ and ZF conceived of the research, SS and MB raised aphids and isolated nucleic acids, WC conducted data analysis, and WC, SS, and GJ wrote the manuscript.

Ethics approval and consent to participate

This is not required for experiments with *R. maidis.*

Competing interests

The authors have no competing financial and non-financial competing interests.

Funding

This research was sponsored by the Defense Advanced Research Projects Agency (DARPA) and was accomplished under cooperative agreement number HR0011-17-2-0053. The views and conclusions contained in this document are those of the authors and should not be interpreted as representing the official policies, either expressed or implied, of DARPA or the U.S. Government. The U.S. Government is authorized to reproduce and distribute reprints for Government purposes notwithstanding any copyright notation hereon.

References

1. Meihls LN, Kaur H, Jander G. Natural variation in maize defense against insect herbivores. Cold Spring Harbor Symp Quant Biol. 2012;77:269-283.
2. McMullen M, Frey M, Degenhardt J. Genetics and biochemistry of insect resistance in maize. In: J.L. Bennetzen and S. Hake, editors. Handbook of Maize: its Biology. New York: Springer; 2009. p. 587.
3. Machado S, Bynum ED, Archer TL, Lascano RJ, Wilson LT, Bordovsky J, et al. Spatial and temporal variability of corn growth and grain yield: Implications for site-specific farming. Crop Science. 2002;42:1564-1576.
4. Blackman RL, Eastop VF. Aphids on the World's Crops. Chichester: Wiley; 2000.
5. Caballero PP, Ramirez CC, Niemeyer HM. Specialisation pattern of the aphid *Rhopalosiphum maidis* is not modified by experience on a novel host. Ent Exp Appl. 2001;100:43-52.
6. Meihls LN, Handrick V, Glauser G, Barbier H, Kaur H, Haribal MM, et al. Natural variation in maize aphid resistance is associated with 2,4-dihydroxy-7-methoxy-1,4-benzoxazin-3-one glucoside methyltransferase activity. Plant Cell. 2013;25:2341-2355.
7. Betsiashvili M, Ahern KR, Jander G. Additive effects of two quantitative trait loci that confer *Rhopalosiphum maidis* (corn leaf aphid) resistance in maize inbred line Mo17. J Exp Bot. 2015;66:571-578.
8. Bing JW, Guthrie WD, Dicke FF, Obrycki JJ. Seedling stage feeding by corn leaf aphid (Homoptera, Aphididae) - Influence on plant development in maize. J Econ Ent. 1991;84:625-632.
9. Carena MJ, Glogoza P. Resistance of maize to the corn leaf aphid: A review. Maydica. 2004;49:241-254.
10. Foott WH, Timmins PR. Effects of infestations by corn leaf aphid, *Rhopalosiphum maidis* (Homoptera-Aphididae), on field corn in southwestern Ontario. Can Ent. 1973;105:449-458.
11. El-Muadhidi MA, Makkouk KM, Kumari SG, Myasser J, Murad SS, Mustafa R. Survey for legume and cereal viruses in Iraq. Phytopathol. Mediterr. 2001;40:224-223.

12. Hawkes JR, Jones RAC. Incidence and distribution of Barley yellow dwarf virus and Cereal yellow dwarf virus in over-summering grasses in a Mediterranean-type environment. Aust J Ag Res. 2005;56:257-270.

13. Jarošová J, Chrpová J, Šíp V, Kundu JK. A comparative study of the Barley yellow dwarf virus species PAV and PAS: distribution, accumulation and host resistance. Plant Pathol. 2013;62:436-443.

14. Power AG, Borer ET, Hosseini P, Mitchell CE, Seabloom EW. The community ecology of barley/cereal yellow dwarf viruses in Western US grasslands. Virus Res. 2011;159:95-100.

15. Krueger EN, Beckett RJ, Gray SM, Miller WA. The complete nucleotide sequence of the genome of Barley yellow dwarf virus-RMV reveals it to be a new Polerovirus distantly related to other yellow dwarf viruses. Front Microbiol. 2013;4.

16. El-Ibrashy MT, El-Ziady S, Riad AA. Laboratory studies on the biology of the corn leaf aphid, *Rhopalosiphum maidis* (Homoptera: Aphididae). Ent Exp Appl. 1972;15:166-174.

17. Gill CC, Metcalfe DR. Resistance in barley to the corn leaf aphid *Rhopalosiphum maidis*. Can J Plant Sci. 1977;57:1063-1070.

18. Lee S, Holman J, Havelka J. Illustrated Catalogue of Aphididae in the Korean Peninsula Part I, Subfamily Aphidinae. Insects of Korea Ser. 9. Deajon, Korea: Korea Research Institute of Bioscience and Biotechnology; 2002.

19. Remaudière G, Naumann-Etienne K. Découverte au Pakistan de l'hôte primaire de *Rhopalosiphum maidis*. C. R. Acad. Agric. Fr. . 1991;77:61-62.

20. Torikura H. Revisional notes on Japanese *Rhopalosiphum*, with keys to species based on the morphs on the primary host. Jap J Ent. 1991;59:257-273.

21. Eastop VF. The males of *Rhopalosiphum maidis* (Fitch) and the discussion of the use of males in aphid taxonomy. Proc. Roy. Ent. Soc. Lond. (A). 1954;29:84-86.

22. Brown PA, Blackman RL. Karyotype variation in the corn leaf aphid, *Rhopalosiphum maidis* (Fitch), species complex (Hemiptera, Aphididae) in relation to host-plant and morphology. Bull Ent Res. 1988;78:351-363.

23. Blackman RA, Brown PA. Morphometric variation within and between populations of *Rhopalosiphum maidis* with a discussion of the taxonomic treatment of permanently parthenogenetic aphids (Homoptera: Aphididae). Ent Gen. 1991;16:97-113.

24. Richards S, Gibbs RA, Gerardo NM, Moran N, Nakabachi A, Stern D, et al. Genome sequence of the pea aphid *Acyrthosiphon pisum*. PLoS Biology. 2010;8:e1000313.

25. Thorpe P, Cock PJ, Bos J. Comparative transcriptomics and proteomics of three different aphid species identifies core and diverse effector sets. BMC Genomics. 2016;17:172.

26. Chen W, Hasegawa DK, Kaur N, Kliot A, Pinheiro PV, Luan J, et al. The draft genome of whitefly *Bemisia tabaci* MEAM1, a global crop pest, provides novel insights into virus transmission, host adaptation, and insecticide resistance. BMC Biol. 2016;14:110.

27. Mathers TC, Chen Y, Kathakottil G, Legeai F, Mugford ST, Baa-Puolet P, et al. Rapid transcriptional plasticity of duplicated gene clusters enables a clonally reproducing aphid to colonize diverse plant species. Genome Biol. 2017;18:27.

28. Wenger JA, Cassone BJ, Legeai F, Johnston JS, Bansal R, Yates AD, et al. Whole genome sequence of the soybean aphid, *Aphis glycines*. Insect Biochem Mol Biol. 2017;epub ahead of print:doi: S0965-1748(17)30005-X [pii] 10.1016/j.ibmb.2017.01.005.

29. Thorpe P, Escudero-Martinez CM, Cock PJA, Eves-van den Akker S, Bos JIB. Shared transcriptional control and disparate gain and loss of aphid parasitism genes. Genome Biol Evol. 2018;epub ahead of print:doi: 5079402 [pii] 10.1093/gbe/evy183.

30. Burger NFV, Botha AM. Genome of Russian wheat aphid an economically important cereal aphid. Stand Genomic Sci. 2017;12:90.

31. Nicholson SJ, Nickerson ML, Dean M, Song Y, Hoyt PR, Rhee H, et al. The genome of *Diuraphis noxia*, a global aphid pest of small grains. BMC Genomics. 2015;16:429.

32. Fulton M, Chunwongse J, tanksley SD. Microprep protocol for extractino of DNA from tomato and other herbaceous plants. Plant Mol Biol Rep. 1995;13:207-209.

33. Chen W, Hasegawa DK, Arumuganathan K, Simmons AM, Wintermantel WM, Fei Z, et al. Estimation of the whitefly *Bemisia tabaci* genome size based on k-mer and flow cytometric analyses. Insects. 2015;6:704-15.

34. Bolger AM, Lohse M, Usadel B. Trimmomatic: A flexible trimmer for Illumina sequence data. Bioinformatics. 2014;30:2114-2120.

35. Zhong S, Joung JG, Zheng Y, Chen YR, Liu B, Shao Y, et al. High-throughput Illumina strand-specific RNA sequencing library preparation. Cold Spring Harb Protoc. 2011;2011:940-949.

36. Kim D, Langmead B, Salzberg SL. HISAT: A fast spliced aligner with low memory requirements. Nat Methods. 2015;12:357-360.

37. Pertea M, Pertea GM, Antonescu CM, Chang TC, Mendell JT, Salzberg SL. StringTie enables improved reconstruction of a transcriptome from RNA-seq reads. Nat Biotechnol. 2015;33:290-295.

38. Li H, Durbin R. Fast and accurate short read alignment with Burrows-Wheeler transform. Bioinformatics. 2009;25:1754-1760.

39. Burton JN, Adey A, Patwardhan RP, Qiu R, Kitzman JO, Shendure J. Chromosome-scale scaffolding of de novo genome assemblies based on chromatin interactions. Nat Biotechnol. 2013;31:1119-1125.

40. Koren S, Walenz BP, Berlin K, Miller JR, Bergman NH, Phillippy AM. Canu: scalable and accurate long-read assembly via adaptive k-mer weighting and repeat separation. Genome Res. 2017;27:722-736.

41. Walker BJ, Abeel T, Shea T, Priest M, Abouelliel A, Sakthikumar S, et al. Pilon: An integrated tool for comprehensive microbial variant detection and genome assembly improvement. PloS One. 2014;9:e112963.

42. Simao FA, Waterhouse RM, Ioannidis P, Kriventseva EV, Zdobnov EM. BUSCO: Assessing genome assembly and annotation completeness with single-copy orthologs. Bioinformatics. 2015;31:3210-3212.

43. Seemann T. Prokka: Rapid prokaryotic genome annotation. Bioinformatics. 2014;30:2068-2069.

44. Zytynska SE, Weisser WW. The natural occurrence of secondary bacterial symbionts in aphids. Ecol Ent. 2015;41:13-26.

45. Han Y, Wessler SR. MITE-Hunter: a program for discovering miniature inverted-repeat transposable elements from genomic sequences. Nucleic Acids Res. 2010;38:e199-e199.

46. Bao Z, Eddy SR. Automated de novo identification of repeat sequence families in sequenced genomes. Genome Res. 2002;12:1269-1276.

47. Benson G. Tandem repeats finder: A program to analyze DNA sequences. Nucleic Acids Res. 1999;27:573-580.

48. Price AL, Jones NC, Pevzner PA. De novo identification of repeat families in large genomes. Bioinformatics. 2005;21 Suppl 1:i351-8.

49. Bao W, Kojima KK, Kohany O. Repbase Update, a database of repetitive elements in eukaryotic genomes. Mobile DNA. 2015;6:11.

50. Cantarel BL, Korf I, Robb SM, Parra G, Ross E, Moore B, et al. MAKER: An easy-to-use annotation pipeline designed for emerging model organism genomes. Genome Res. 2008;18:188-196.

51. Gotoh O. Direct mapping and alignment of protein sequences onto genomic sequence. Bioinformatics. 2008;24:2438-44.

52. Stanke M, Waack S. Gene prediction with a hidden Markov model and a new intron submodel. Bioinformatics. 2003;19 Suppl 2:ii215-25.

53. Hoff KJ, Lange S, Lomsadze A, Borodovsky M, Stanke M. BRAKER1: Unsupervised RNA-Seq-Based Genome Annotation with GeneMark-ET and AUGUSTUS. Bioinformatics. 2015.

54. Lomsadze A, Burns PD, Borodovsky M. Integration of mapped RNA-Seq reads into automatic training of eukaryotic gene finding algorithm. Nucleic Acids Res. 2014;42:e119.

55. Mitchell A, Chang HY, Daugherty L, Fraser M, Hunter S, Lopez R, et al. The InterPro protein families database: The classification resource after 15 years. Nucleic Acids Res. 2015;43:D213-21.

56. Conesa A, Gotz S, Garcia-Gomez JM, Terol J, Talon M, Robles M. Blast2GO: A universal tool for annotation, visualization and analysis in functional genomics research. Bioinformatics. 2005;21:3674-3676.

57. Li L, Stoeckert CJ, Jr., Roos DS. OrthoMCL: Identification of ortholog groups for eukaryotic genomes. Genome Res. 2003;13:2178-2189.

58. Edgar RC. MUSCLE: Multiple sequence alignment with high accuracy and high throughput. Nucleic Acids Res. 2004;32:1792-1797.

59. Capella-Gutierrez S, Silla-Martinez JM, Gabaldon T. TrimAl: A tool for automated alignment trimming in large-scale phylogenetic analyses. Bioinformatics. 2009;25:1972-1973.

60. Guindon S, Delsuc F, Dufayard JF, Gascuel O. Estimating maximum likelihood phylogenies with PhyML. Methods Mol Biol. 2009;537:113-37.

61. Papasotiropoulos V, Tsiamis G, Papaioannou C, Ioannidis P, Klossa-Kilia E, Papapanagiotou AP, et al. A molecular phylogenetic study of aphids (Hemiptera: Aphididae) based on mitochondrial DNA sequence analysis. J Biol Res. 2013;20:195.

62. Crisp A, Boschetti C, Perry M, Tunnacliffe A, Micklem G. Expression of multiple horizontally acquired genes is a hallmark of both vertebrate and invertebrate genomes. Genome Biol. 2015;16:50.

63. Larkin MA, Blackshields G, Brown NP, Chenna R, McGettigan PA, McWilliam H, et al. Clustal W and Clustal X version 2.0. Bioinformatics. 2007;23:2947-8.

64. Nikoh N, McCutcheon JP, Kudo T, Miyagishima SY, Moran NA, Nakabachi A. Bacterial genes in the aphid genome: absence of functional gene transfer from Buchnera to its host. PLoS Genet. 2010;6:e1000827.

65. Chung SH, Jing X, Luo Y, Douglas AE. Targeting symbiosis-related insect genes by RNAi in the pea aphid-*Buchnera* symbiosis. Insect Biochem Mol Biol. 2018;95:55-63.

66. Moran NA, Jarvik T. Lateral transfer of genes from fungi underlies carotenoid production in aphids. Science. 2010;328:624-627.

67. Woldman Y, Appling DR. A general method for determining the contribution of split pathways in metabolite production in the yeast Saccharomyces cerevisiae. Metab Eng. 2002;4:170-81.

68. Field LM. Methylation and expression of amplified esterase genes in the aphid *Myzus persicae* (Sulzer). Biochem J. 2000;349:863-868.

69. Huang FF, Chai CL, Zhang Z, Liu ZH, Dai FY, Lu C, et al. The UDP-glucosyltransferase multigene family in *Bombyx mori*. BMC genomics. 2008;9:563.

70. Dermauw W, Van Leeuwen T. The ABC gene family in arthropods: comparative genomics and role in insecticide transport and resistance. Insect Biochem Mol Biol. 2014;45:89-110.

71. Jones P, Binns D, Chang HY, Fraser M, Li W, McAnulla C, et al. InterProScan 5: Genome-scale protein function classification. Bioinformatics. 2014;30:1236-1240.

72. Oakeshott JG, Claudianos C, Campbell PM, Newcomb RD, Russell RJ. Biochemical genetics and genomics of insect esterases. In: L.I. Gilbert and S.S. Gill, editors. Insect Pharmacology: Channels, Receptors, Toxins and Enzyme. Amsterdam: Academic Press; 2010. p. 392.

Table 1. Assembly statistics of seven aphid genomes.

	R. maidis	*Ap. glycines*	*M. persicae*	*Ac. pisum*	*M. cerasi*	*R. padi*	*D. noxia*
Genome assembly							
Assembly size (Mb)	326.0	302.9	347.3	541.6	405.7	319.4	393.0
Contig Count	689	66,000	8,249	60,623	56,508	16,689	49,357
Contig N50 (bp)	9,046,396	15,844	144,275	28,192	17,908	96,831	12,578
Scaffold Count	220	8,397	4,022	23,924	49,286	15,587	5,641
Scaffold N50 (bp)	95,599,851	174,505	435,781	518,546	23,273	116,185	397,774
Max. scaffold length (Mb)	116.8	1.4	2.2	3	0.2	0.6	2.1
Min. scaffold length (kb)	1.1	2	0.9	0.2	1	1	0.9
Genomic features							
Gene count	17,647	19,182	18,529	36,195	28,688	26,286	25,987
Transcript length (bp)	1,834.6	1,520.1	1,838.7	1,964.1	NA	NA	NA
CDS length (bp)	1,242.04	1,240.3	1,328.3	1,157.6	952.7	1155.09	970.2
exon length (bp)	210.02	245.5	299.2	394.7	NA	NA	NA
exon count/gene	6.31	6.19	6.14	4.97	NA	NA	NA

NA: This information could not be retrieved from the annotation files.

Table 2. Repeats in the *R. maidis* genome assembly

Class	No. of Copies	Length (bp)	Coverage of genome (%)
SINE	27,308	7,085,803	2.17
LINE	6,688	1,596,259	0.49
LTR	3,445	896,470	0.28
DNA transposon	53,797	9,499,710	2.91
MITE	64,663	14,240,430	4.37
Unclassified	49,627	18,375,079	5.64
Other*	375,149	17,360,944	5.33
Total	580,677	69,054,695	21.18

*Other: microsatellites/simple repeats/low complexity sequences

Table 3. Horizontally transferred genes in *R. maidis*

**Gene ID**	**Function description**	**Orign**	**RPKM**
Rma07998	Peptidase U61; LD-carboxypeptidase A	Bacteria	1.63
Rma09603	Carbamoylphosphate synthase large subunit	Bacteria	10.19
Rma01752	Lycopene cyclase phytoene synthase	Fungi	6.89
Rma01753	Carotenoid desaturase	Fungi	36.65
Rma01754	Lycopene cyclase phytoene synthase	Fungi	4.01
Rma01756	Lycopene cyclase phytoene synthase	Fungi	2.67
Rma01758	Lycopene cyclase phytoene synthase	Fungi	6.51
Rma01759	Lycopene cyclase phytoene synthase	Fungi	6.13
Rma01760	Carotenoid desaturase	Fungi	18.19
Rma08772	Leucine Rich Repeat family protein	Protozoa	0
Rma11572	Antigenic protein, putative	Protozoa	0
Rma10344	Ankyrin repeat protein	Algae	0.07
Rma11418	Ankyrin repeat protein	Algae	0.03
Rma12243	Ankyrin repeat protein	Algae	0.2
Rma13322	Ankyrin repeat protein	Algae	5.61
Rma13584	Ankyrin repeat protein	Algae	0.1
Rma14036	Ankyrin repeat protein	Algae	0.22
Rma15269	Ankyrin repeat protein	Algae	0.09
Rma16213	Ankyrin repeat protein	Algae	16.14
Rma16838	Ankyrin repeat protein	Algae	0.21

Table 4. Numbers of predicted detoxification genes in seven aphid species

	R. maidis	*Ap. glycines*	*M. persicae*	*Ac. pisum*	*M. cerasi*	*R. padi*	*D. noxia*
Cytochrome P450s	59	61	67	82	74	67	60
Glutathione S-transferases	10	12	13	36	12	11	11
Carboxylesterases	23	31	37	48	36	34	32
UDP-glucuronosyltransferases	43	47	57	72	48	55	43
ABC transporters	68	74	67	126	68	71	63
Total	203	225	241	364	238	238	209

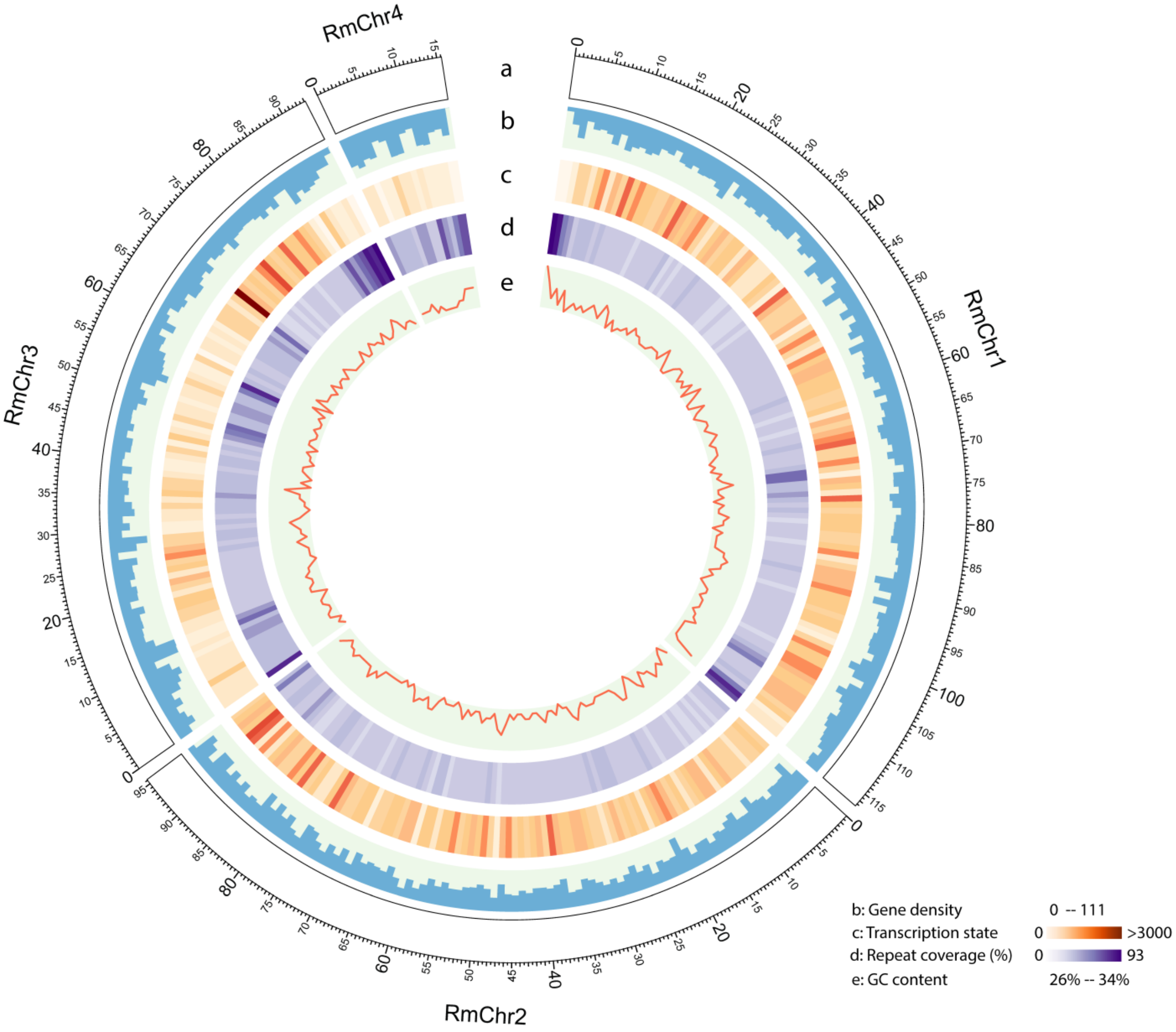

Figure 1. *Rhopalosiphum maidis* genome landscape. (a) Ideogram of the four *R. maidis* pseudochromosomes at the Mb scale. (b) Gene density represented as number of genes per Mb. (c) Transcription state. The transcription level was estimated by read counts per million mapped reads in 1-Mb windows. (d) Percentage of coverage of repeat sequences per Mb. (e) GC content in 1-Mb windows. The four *R. maidis* pseudo-chromosomes represented 98.4% of the genome assembly. This figure was generated using Circos (http://circos.ca/).

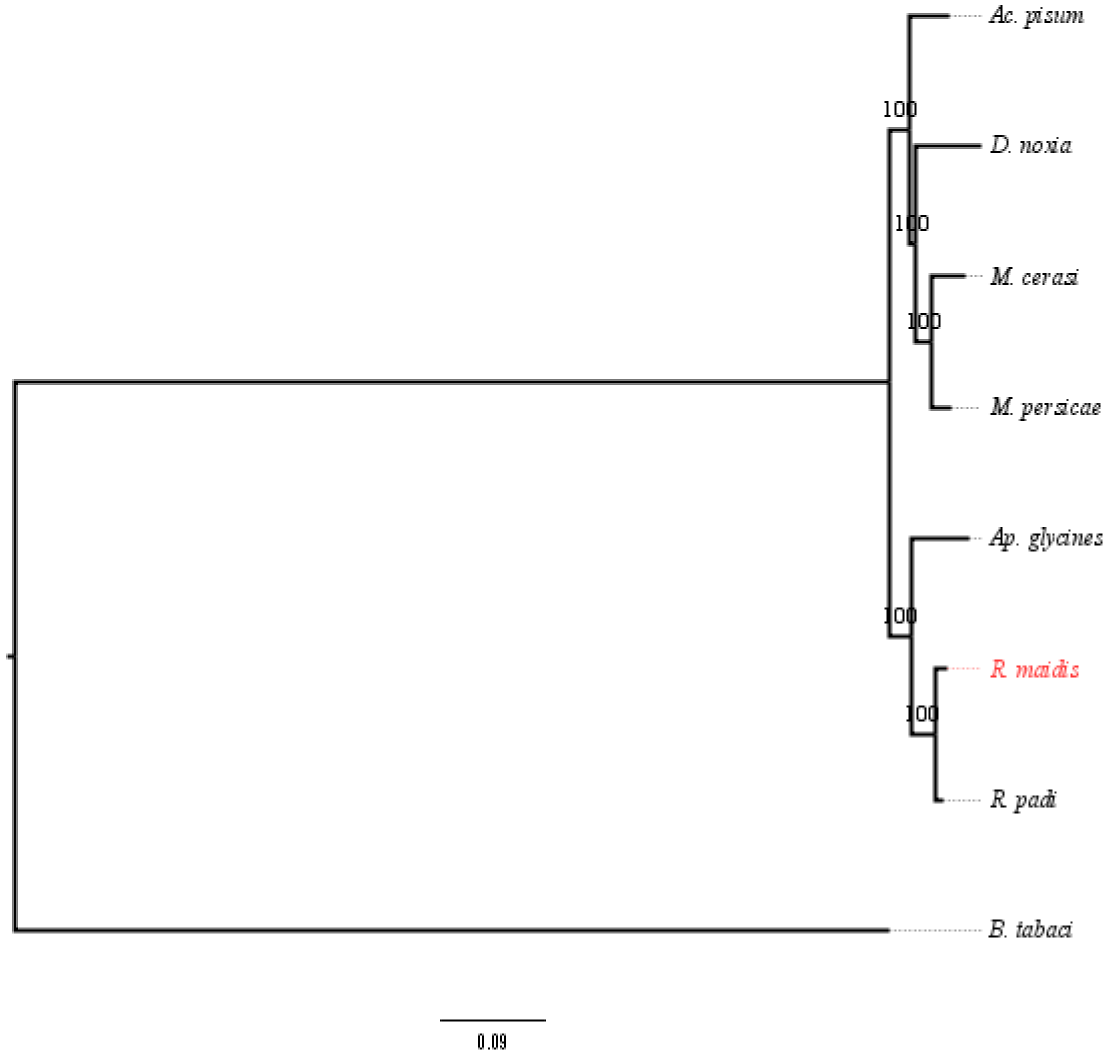

Figure 2. Phylogenetic relationships of *R. maidis* and 7 other arthropod species. *B. tabaci* was used as the outgroup taxon.

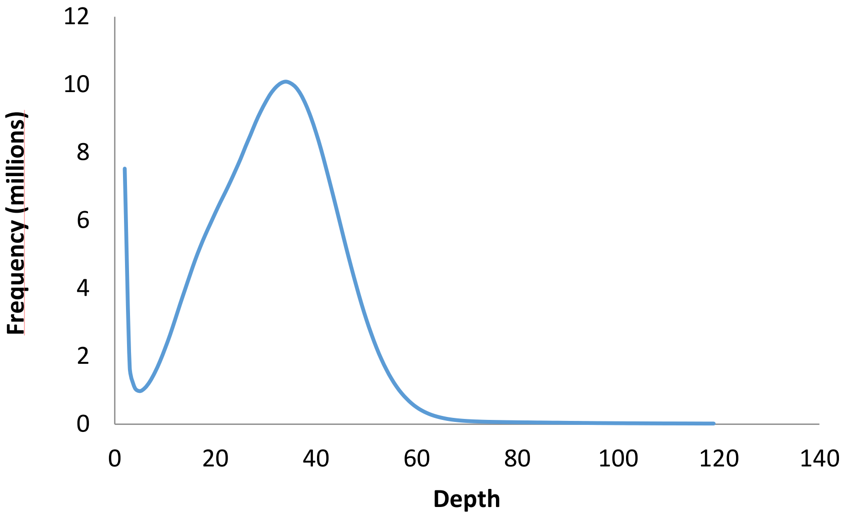

Figure S1. K-mer (K=31) distribution of Illumina genome sequencing reads of *R. maidis*. The total count of K-mers was 11,495,021,417, and the peak of K-mer depth was 34. The genome size of *R. maidis* was calculated by dividing the total K-mer count by the peak depth, which was approximately 338 Mb.

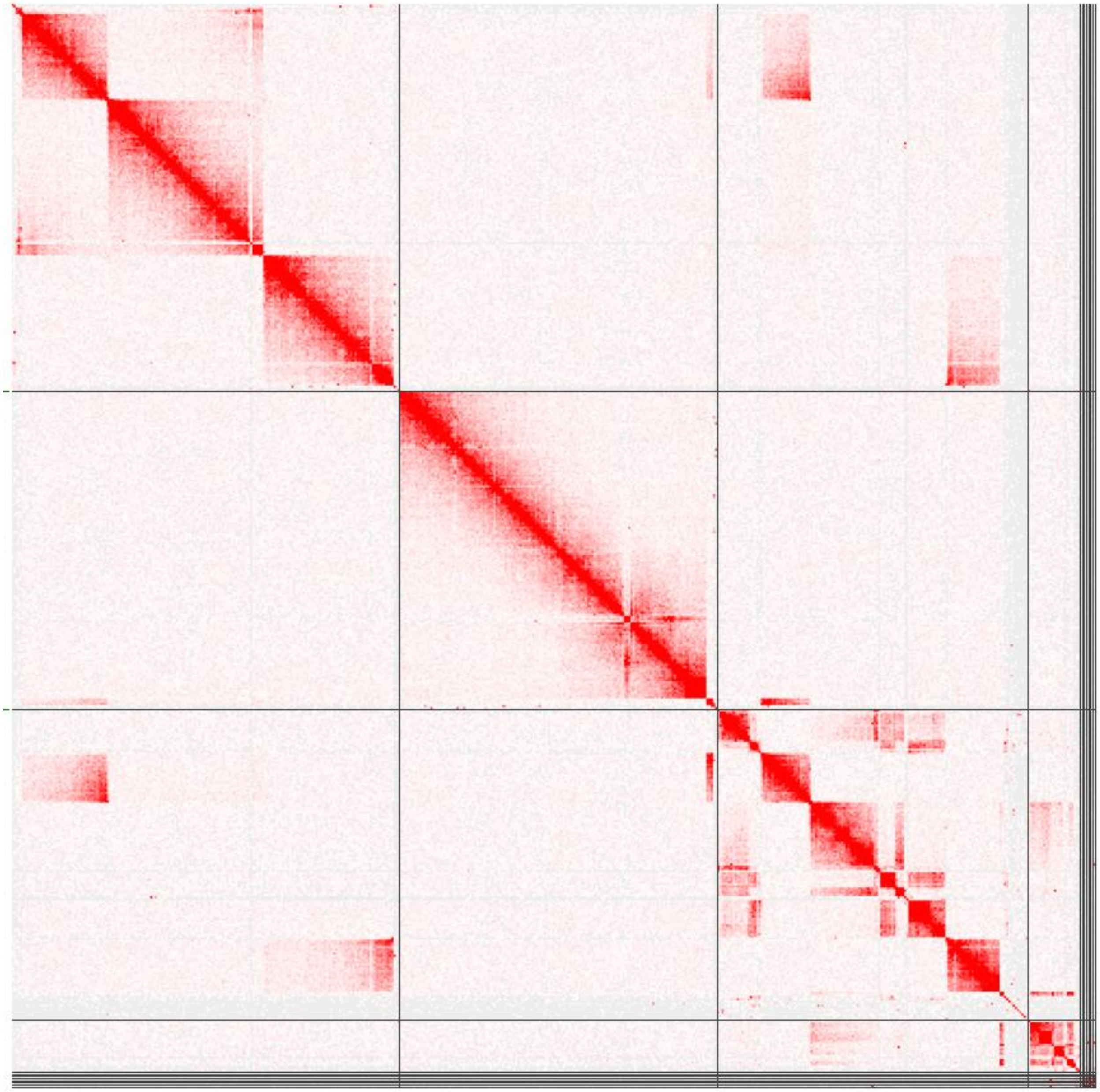

Figure S2. Hi-C contact map of the *R. maidis* genome

A.

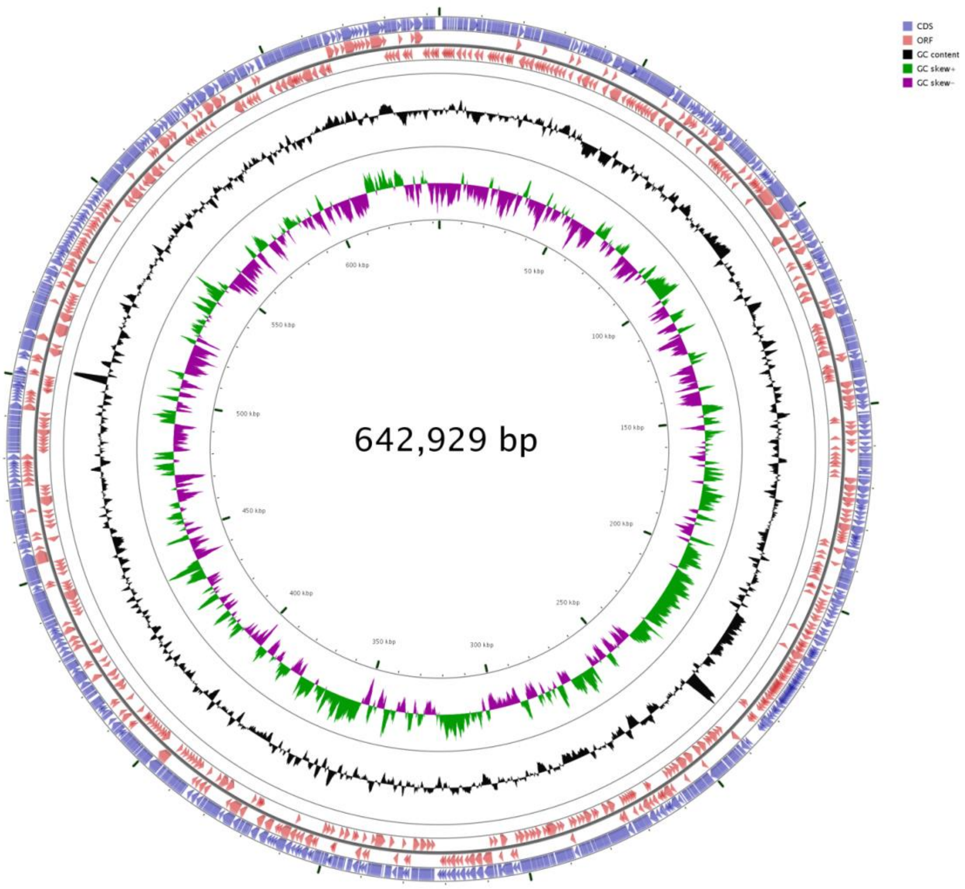

B.

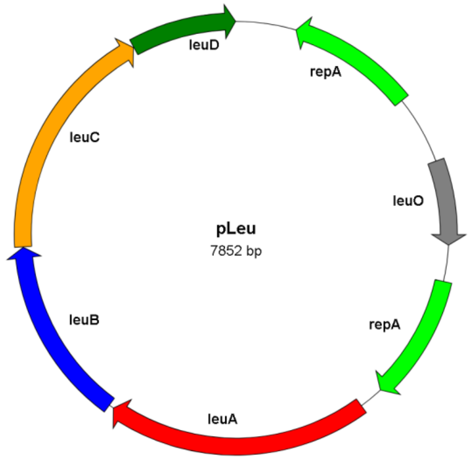

C.

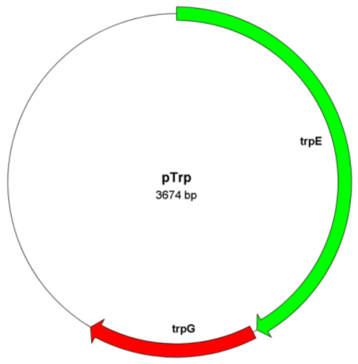

Figure S3. Circular view of the genome of the *Rhopalosiphum maidis* endosymbiont, *Buchnera aphidicola* (A) and its plasmids pLeu (B) and pTrp (C).

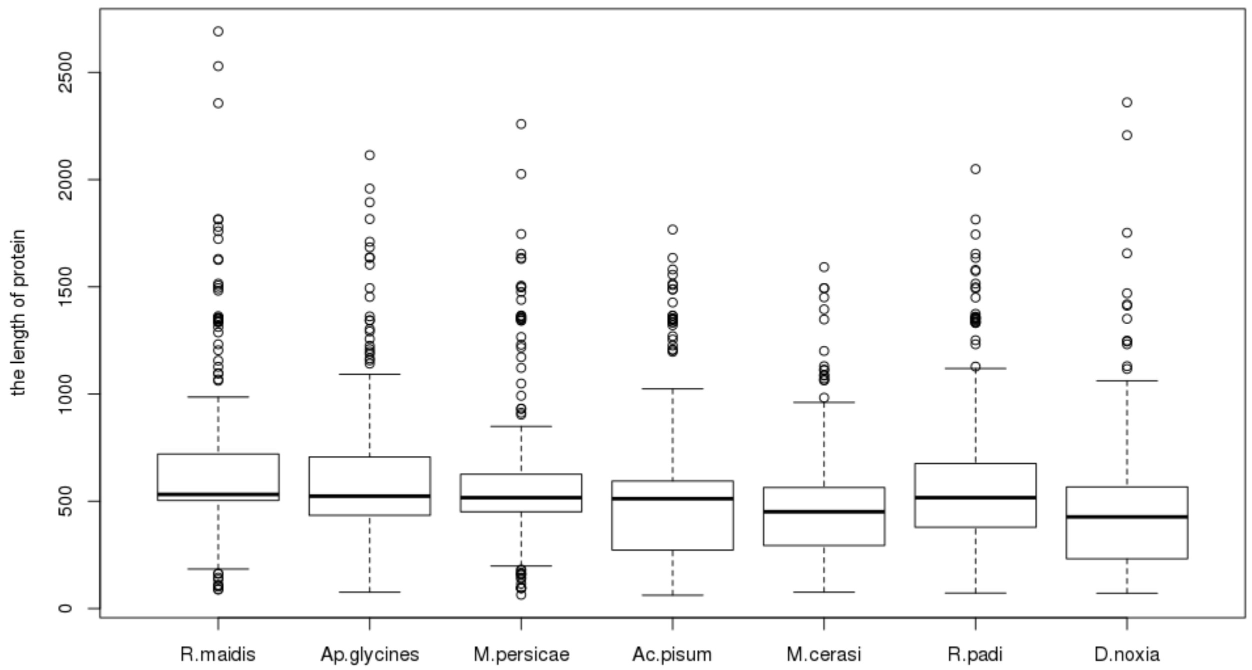

Figure S4. Length distribution of protein sequences of detoxification gene families in seven aphid species.

Supplementary Tables

Click here to access/download
Supplementary Material
R maidis genome Supplementary Tables-101118.xlsx
