# Supplementary material for: Genome sequence of the corn leaf aphid (Rhopalosiphum maidis Fitch)
Source: Gigascience. 2019 Apr 6;8(4):giz033. doi: 10.1093/gigascience/giz033 (PMC6451198; doi:10.1093/gigascience/giz033)
Supplement: GIGA-D-18-00405_Revision_2.pdf [file giz033_giga-d-18-00405_revision_2.pdf]

GigaScience

Genome sequence of the corn leaf aphid (Rhopalosiphum maidis Fitch)

--Manuscript Draft--

Manuscript Number:	GIGA-D-18-00405R2
Full Title:	Genome sequence of the corn leaf aphid (Rhopalosiphum maidis Fitch)
Article Type:	Data Note
Funding Information:	Defense Advanced Research Projects Agency (HR0011-17-2-0053) — Dr. Georg Jander
Abstract:	Background: The corn leaf aphid (Rhopalosiphum maidis Fitch) is the most economically damaging aphid pest on maize (Zea mays), one of the world's most important grain crops. In addition to causing direct damage due to the removal of photoassimilates, R. maidis transmits several destructive maize viruses, including Maize yellow dwarf virus, Barley yellow dwarf virus, Sugarcane mosaic virus, and Cucumber mosaic virus.<br>Findings: The genome of a parthenogenetically reproducing R. maidis clone was assembled with a combination of PacBio (207-fold coverage) and Illumina (83-fold coverage) sequencing. The 689 assembled contigs, which have an N50 size of 9.0 Mb and a low level of heterozygosity, were clustered using Phase Genomics Hi-C interaction maps. Consistent with the commonly observed 2n = 8 karyotype of R. maidis, most of the contigs (473 spanning 321 Mb) were successfully oriented into four scaffolds. The genome assembly captured the full length of 95.8% of the core eukaryotic genes, indicating that it is highly complete. Repetitive sequences accounted for 21.2% of the assembly, and a total of 17,629 protein-coding genes were predicted with integrated evidence from ab initio and homology-based gene predictions and transcriptome sequences generated with both PacBio and Illumina. An analysis of likely horizontally transferred genes identified two from bacteria, seven from fungi, two from protozoa, and nine from algae. Repeat elements, transposons, and genes encoding likely detoxification enzymes (cytochrome P450s, glutathione S-transferases, carboxylesterases, UDP-glucosyltransferases, and ABC transporters) were identified in the genome sequence. Other than Buchnera aphidicola, (642,929 bp, 602 genes) no endosymbiont bacteria were found in R. maidis.<br>Conclusions: A high-quality R. maidis genome was assembled at the chromosome level. This genome sequence will enable further research related to ecological interactions, virus transmission, pesticide resistance, and other aspects of R. maidis biology. It also serves as a valuable resource for comparative investigation of other aphid species.
Corresponding Author:	Georg Jander<br>Boyce Thompson Institute for Plant Research<br>Ithaca, NY UNITED STATES
Corresponding Author Secondary Information:	
Corresponding Author's Institution:	Boyce Thompson Institute for Plant Research
Corresponding Author's Secondary Institution:	
First Author:	Wenbo Chen
First Author Secondary Information:	
Order of Authors:	Wenbo Chen
	Sara Shakir
	Mahdiyeh Bigham
	Annett Richter
	Zhangjun Fei

Powered by Editorial Manager® and ProduXion Manager® from Aries Systems Corporation

	Georg Jander
**Order of Authors Secondary Information:**	
**Response to Reviewers:**	>>In response to reviewer comments, we have made the described change to our manuscript. The change is marked in red in the resubmitted manuscript.<br><br>Reviewer #3:<br>The authors have done a good job in addressing the previous comments and improved the manuscript significantly. In particular, they provided sufficient information on the materials and methods in the revised manuscript. I am almost satisfied with the revision. I have only a single issue to ask the authors to confirm as below:<br><br>* L157 : DNA samples were transferred to a Covaris G-tube ... : G-tube is usually used to shear DNA into fragments ranging from 6kb to 20kb (see manufacture's website); it is typically used in long-read NGS library prep. The target fragmentation size of the PCR-free Illumina library was 550 bp as described in the manuscript (L161). I concern the discrepancy.<br><br>>>This was a mistake. We have changed “Covaris G-tube” to “Covaris microTUBE” in this sentence.
**Additional Information:**	
**Question**	**Response**
Are you submitting this manuscript to a special series or article collection?	No
**Experimental design and statistics**<br><br>Full details of the experimental design and statistical methods used should be given in the Methods section, as detailed in our Minimum Standards Reporting Checklist. Information essential to interpreting the data presented should be made available in the figure legends.<br><br>Have you included all the information requested in your manuscript?	Yes
**Resources**<br><br>A description of all resources used, including antibodies, cell lines, animals and software tools, with enough information to allow them to be uniquely identified, should be included in the Methods section. Authors are strongly encouraged to cite Research Resource Identifiers (RRIDs) for antibodies, model organisms and tools, where possible.<br><br>Have you included the information	Yes

Powered by Editorial Manager® and ProduXion Manager® from Aries Systems Corporation

requested as detailed in our Minimum Standards Reporting Checklist?	
**Availability of data and materials**<br><br>All datasets and code on which the conclusions of the paper rely must be either included in your submission or deposited in publicly available repositories (where available and ethically appropriate), referencing such data using a unique identifier in the references and in the "Availability of Data and Materials" section of your manuscript.<br><br>Have you have met the above requirement as detailed in our Minimum Standards Reporting Checklist?	Yes

Powered by Editorial Manager® and ProduXion Manager® from Aries Systems Corporation

Manuscript
Click here to access/download;Manuscript;R maidis genome MS 2-7-19.docx
Click here to view linked References

Genome sequence of the corn leaf aphid (*Rhopalosiphum maidis* Fitch)

Wenbo Chen[1], Sara Shakir[1,3], Mahdiyeh Bigham[1], Annett Richter[1], Zhangjun Fei[1,2], and Georg Jander[1,*]

[1]Boyce Thompson Institute, 533 Tower Road, Ithaca NY 14853

[2]US Department of Agriculture-Agricultural Research Service, Robert W. Holley Center for Agriculture and Health, Ithaca, NY, 14853, USA

[3]Present address: Plant Genetics Lab, Gembloux Agro-Bio Tech, University of Liège, Gembloux, Belgium

*To whom correspondence should be addressed:
Georg Jander
Boyce Thompson Institute
533 Tower Road
Ithaca NY 14853
Phone: (1) 607-254-1365
Email: gj32@cornell.edu

Abstract

Background: The corn leaf aphid (*Rhopalosiphum maidis* Fitch) is the most economically damaging aphid pest on maize (*Zea mays*), one of the world's most important grain crops. In addition to causing direct damage due to the removal of photoassimilates, *R. maidis* transmits several destructive maize viruses, including *Maize yellow dwarf virus*, *Barley yellow dwarf virus*, *Sugarcane mosaic virus*, and *Cucumber mosaic virus.*

Findings: The genome of a parthenogenetically reproducing *R. maidis* clone was assembled with a combination of PacBio (207-fold coverage) and Illumina (83-fold coverage) sequencing. The 689 assembled contigs, which have an N50 size of 9.0 Mb and a low level of heterozygosity, were clustered using Phase Genomics Hi-C interaction maps. Consistent with the commonly observed 2n = 8 karyotype of *R. maidis*, most of the contigs (473 spanning 321 Mb) were successfully oriented into four scaffolds. The genome assembly captured the full length of 95.8% of the core eukaryotic genes, indicating that it is highly complete. Repetitive sequences accounted for 21.2% of the assembly, and a total of 17,629 protein-coding genes were predicted with integrated evidence from *ab initio* and homology-based gene predictions and transcriptome sequences generated with both PacBio and Illumina. An analysis of likely horizontally transferred genes identified two from bacteria, seven from fungi, two from protozoa, and nine from algae. Repeat elements, transposons, and genes encoding likely detoxification enzymes (cytochrome P450s, glutathione S-transferases, carboxylesterases, UDP-glucosyltransferases, and ABC transporters) were identified in the genome sequence. Other than *Buchnera aphidicola*, (642,929 bp, 602 genes) no endosymbiont bacteria were found in *R. maidis.*

Conclusions: A high-quality *R. maidis* genome was assembled at the chromosome level. This genome sequence will enable further research related to ecological interactions, virus transmission, pesticide resistance, and other aspects of *R. maidis* biology. It also serves as a valuable resource for comparative investigation of other aphid species.

Keywords: corn leaf aphid, genome, annotation, *Rhopalosiphum maidis*

Data Description

Introduction

Maize (*Zea mays*), the world's most productive grain crop, is susceptible to more than 90 species of herbivorous insects [1-3]. Among aphids that feed on maize, the corn leaf aphid (*Rhopalosiphum maidis* Fitch) is the most commonly encountered, particularly in tropical and warmer temperate areas [4]. Relative to other maize-feeding aphids (*Rhopalosiphum padi*, *Schizaphis graminum*, *Sitobion avenae*, and *Metopolophium dirhodum*), *R. maidis* exhibits a greater tolerance of benzoxazinoids, the most abundant class of maize defensive metabolites [5]. However, the mechanism of aphid resistance to these plant toxins is not known, and natural variation in benzoxazinoid content among maize inbred lines nevertheless influences growth and reproduction of *R. maidis* [6, 7].

Damage caused to maize by *R. maidis* takes several forms, with the resulting yield losses being`quite variable from year to year. Growth and yield are reduced through the removal of photosynthates by large numbers of aphids [8]. On flowering-stage maize, aphids tend to congregate on the tassels, where large amounts of honeydew can prevent the release of pollen from the anthers, thereby reducing seed set by up to 90% [9, 10]. Additional damage comes from the transmission of several important maize viruses, including *Maize yellow dwarf virus*, *Barley yellow dwarf virus*, *Sugarcane mosaic virus*, and *Cucumber mosaic virus* [11-15], by *R. maidis*.

In addition to feeding on maize, *R. maidis* infests a variety of other monocot species, including barley, oat, rice, rye, sorghum, sugarcane, and wheat [4]. In one study, barley was reported as the most suitable grain crop host [16]. However, as in the case of maize, there is also considerable within-species variation for *R. maidis* resistance in barley [17].

The origin of *R. maidis* is likely in Asia, and it has been subsequently introduced in most grain-growing areas of the world [4]. In almost all parts of its range, *R. maidis* is anholocyclic, *i.e.* reproduction occurs entirely by parthenogenesis. However, sexual reproduction has been reported in Pakistan and Korea, with *Prunus* ssp. as the primary host [18, 19]. In populations in Japan and Kenya, males but not sexually reproducing females have been found [20, 21]. Consistent with the sometimes permanently parthenogenetic life cycle of *R. maidis,* there is within-species variation in the chromosome numbers. Karyotypes of 2n = 8, 9, and 10 have been reported. There also is

evidence of host specificity among the karyotypes. Whereas *R. maidis* strains on maize tend to have 2n = 8, those on barley generally have 2n = 10 [22, 23].

Here we report the *R. maidis* isolate BTI-1 genome sequence, assembled using Pacific Biosciences (PacBio) sequencing, Illumina sequencing, and Phase Genomics Hi-C scaffolding. Contigs were assembled using PacBio, which provides >10,000 bp read lengths, but has an approximately 10% error rate [24]. These errors were corrected by Illumina sequencing reads, with 151 bp paired-end read lengths and a 0.1% error rate. Contigs assembled from PacBio were linked into chromosome-scale scaffolds using Hi-C, which identifies long-range contact information for DNA sequences [25, 26]. To assist in annotation of the *R. maidis* genome, we sequenced cDNA libraries using both PacBio sequencing to get full-length transcripts (Iso-Seq) and Illumina sequencing (RNA-Seq) to get a higher sequence accuracy. Comparisons to six previously published aphid genomes [27-33] showed an improved assembly, with most of the sequences assembled into four scaffolds, consistent with the 2n = 8 karyotype of *R. maidis* on maize. Analysis of the assembled *R. maidis* genome identified horizontally transferred genes, repetitive elements, and likely xenobiotic detoxification enzymes.

Sampling and genome sequencing

Insect Colony. BTI-1, a corn leaf aphid (*R. maidis*) isolate, which was originally collected from maize (*Z. mays*) in New York State, was obtained from Stewart Gray (USDA Plant Soil and Nutrition Laboratory, Ithaca, NY, USA). An isogenic colony was started from a single parthenogenetic female *R. maidis* and was maintained on barley (*Hordeum vulgare*) prior to the collection of insects for genome and transcriptome sequencing.

Genome Sequencing. Genomic DNA was prepared from 100-200 mg of fresh mixed-instar *R. maidis* tissue using a previously described protocol [34]. Briefly, mixed-instar whole aphids collected from barley were ground in liquid nitrogen with a mortar and pestle, and then incubated at 65°C in 400 µl microprep buffer made up of DNA extraction buffer [0.35 M sorbitol, 0.1 M Tris-base, pH7.5, 5 mM ethylenediaminetetraacetic acid (EDTA)], nuclei lysis buffer [0.2 M Tris-base, pH 7.5, 0.05 M EDTA, 2 M NaCl, 2% cetyltrimethylammonium bromide (CTAB)], 5% sarkosyl, and 0.5% sodium bisulfite (added right before use) for 30 min. The cooled-down solution was then treated with 400 µl chloroform:isoamyl alcohol (24:1), vortexed vigorously, and centrifuged for 10 min at 14,000 *g*. To the upper aqueous phase 3 µl of Rnase A was added and

the samples were incubated for 15 min at 37°C followed by adding 400 µl chloroform:isoamyl alcohol (24:1), vortexing vigorously, and repeating the centrifugation step for 10 min at 14,000 g. To precipitate the DNA, 200 µl of ice-cold 2-propanol was added and gently inverted. The DNA was pelleted by centrifugation at 4°C at 14,000 *g* for 10 min. The DNA pellet was washed with 70% ethanol and, after air drying, was dissolved in 50 µl of nuclease-free water. The quantity and quality of aphid genomic DNA was assessed using a Qubit 3 fluorometer (Thermo Fisher, Waltham, MA, USA) and a Bioanalyzer DNA12000 kit (Agilent, Santa Clara, CA, USA), respectively. Approximately 20 µg of DNA was used for PacBio library construction and sequencing according to the manufacturer's (Pacific Biosciences, Menlo Park, CA, USA) instructions for SMRT 20 kb DNA template preparation, using the Pacific Biosciences Sequel 2.0 sequencing enzyme and chemistry, respectively. Briefly, aphid DNA was first re-purified using a 0.5× AMPure XP (Beckman Coulter, Indianapolis, IA, USA) purification step (0.5× AMPure beads added, by volume, to the DNA sample dissolved in 150 µl Elution Buffer, vortexed for 10 min at 2,000 *g*, followed by two washes with 70% alcohol and finally diluted in Elution Buffer), to remove small fragments and/or biological contaminants. The DNA was then sheared to 25-30 kb using a Covaris G-tube (Covaris, Wobern, MA, USA) and an Eppendorf 5424 centrifuge (Eppendorf, Hamburg, Germany) at 3,000 *g*. The DNA was further purified with 0.5× AMPure XP and the average fragment size was assessed with the Agilent Bioanalyzer DNA12000 kit. The purified DNA sample was taken through DNA damage and end-repair steps. Briefly, the DNA fragments, after processing with 0.18 U/ µl of P6 polymerase, were repaired using DNA damage repair solution (1× DNA damage repeat buffer, 1× NAD+, 1 mM ATP high, 0.1 mM dNTP, and 1× DNA damage repeat mix) in a volume of 52 µl and incubated at 37°C for 20 min. DNA ends were repaired by adding 1× end repair mix to the solution, which was incubated at 25°C for 10 min, followed by a second 0.45× Ampure XP purification step.

SMRTbell library preparation (Pacific Biosciences) was performed as follows: 0.75 µM of blunt adapter was added to the DNA, followed by 1× template prep buffer, 0.05 mM ATP low, and 0.75 U/µl T4 ligase to ligate the SMRTbell adapters to the DNA fragments in the final volume of 40 µl. This solution was incubated at 25°C overnight, followed by 10 min ligase denaturation step at 65°C. After ligation, the library was treated with an exonuclease cocktail to remove unligated DNA fragments using a solution of 1.81 U/µl Exonuclease III and 0.18 U/µl Exonuclease VII and then incubated at 37°C for 1 h. Two additional 0.45× Ampure XP purification steps were

performed to remove <1,000-bp molecular-weight DNA and organic contaminants. Further size-selection of 17-50 kb was performed on Sage BluePippin (Sage Science, Beverly, MA, USA). The 0.75 % agarose gel was run for 4.5 hours using the manufacturer's protocol. An additional DNA damage repair step was performed after the size selection by incubating the template at 37°C for 30 minutes, followed by 0.8× AMP bead purification. The size of the resulting fragments was confirmed using the Agilent bioanalyzer DNA12000 kit, and the mass was quantified using an Invitrogen Qubit 3 fluorometer (Thermo Fisher) before proceeding with primer annealing, polymerase and DNA sequencing on a Sequel system (Pacific Biosciences). Polymerase 2.0 binding to the template was performed by incubating for 4 hours at 30°C. The binding complex was then diluted and diffusion-loaded at 6 pM on the plate on Sequel 5.0 system and sequenced on a Sequel machine with 2.0 chemistry recording 10-h movies. Sequencing was conducted at the Icahn Institute and Department of Genetics and Genomic Sciences, Icahn School of Medicine at Mount Sinai (New York, NY, USA). The raw data analysis was performed on SMRTLink 5.0. A total of 16 SMRT Cells were run on the PacBio Sequel platform, yielding 70 Gb raw sequence data (Supplementary Data S1) for the *R. maidis* genome, which was initially estimated to be 338 Mb using the k-mer approach [35] (Figure 1).

For short-read sequencing, one paired-end library was constructed using the Illumina TruSeq DNA sample preparation kit (Illumina, San Diego, CA, USA), following the manufacturer’s instructions. The quantity of DNA was measured using an Invitrogen Qubit 3 fluorometer, and ~2 µg of DNA was normalized in resuspension buffer (RSB) in a volume of 55 µl, vortexed at 1,800 *g* for 2 minutes followed by centrifugation at 280 *g* for 1 minute. For fragmentation, 52.5 µl of the DNA samples were transferred to a Covaris microTUBE and centrifuged at 280 *g* for 5 seconds. The DNA was purified using sample purification beads, which were washed twice with 80% ethanol and dissolved in 50 µl of resuspension buffer. Purified DNA was end-repaired by adding 40 µl of end repair mixture and incubating at 30°C for 30 minutes. The library with an insert size of approximately 550 bp was purified using sample purification beads (SPB) and all the remaining large and small DNA fragments were removed according to manufacturer’s protocol. Briefly, to remove large DNA fragments, 92 µl of SPB were diluted in 92 µl of PCR-grade water, vortexed and 160 µl of this solution was added to the purified DNA, vortexed at 1,800 *g* for 2 minutes, incubated at 23ºC for 5 min and centrifuged again at 280 *g* for 1 minute. Two hundred fifty µl of supernatant were transferred to a clean-up end repair plate. To

remove small DNA fragments, 30 µl of SPB were added to the supernatant, vortexed at 1,800 *g* for 2 minutes, incubated at 23ºC for 5 minutes and centrifuged at 280 *g* for 1 minute. The supernatant was discarded, SPB were washed twice with 80% ethanol and DNA was dissolved in 15 µl of resuspension buffer. Purified DNA was adenylated at the 3' ends by adding 2.5 µl of A-Tailing control and 12.5 µl of A-Tailing mixture (New England Biolabs, Ipswich, MA, USA) to the sample with a final volume of 30 µl, vortexed at 1,800 *g* for 2 min followed by a first incubation at 37°C for 30 min, a second incubation at 70°C for 5 min and a final incubation on ice for 5 min.

A TruSeq PCR free library (Illumina) was prepared as follows: 2.5 µl of adapters were added to the DNA, followed by adding 2.5 µl of ligation mixture, 2.5 µl of ligation control, and incubation at 30°C for 10 min. The ligation step was stopped by adding 5 µl of ligation stop buffer and ligated fragments were purified using sample purification beads, which were washed twice with 80% ethanol. The library was quantified with the KAPA Library Quantification Kit (Roche, Basel, Switzerland) and the fragment size of the library was verified using an Agilent Technology 2100 bioanalyzer. Sequencing was performed on an Illumina HiSeq 2500 system, which yielded about 75 Gb of raw sequence data (Supplementary Data S1). Raw Illumina reads were processed to remove duplicated read pairs, which were defined as having identical bases in the first 100 bp of both left and right reads, and only one read pair from each duplicated sequence was kept. Illumina adapters and low-quality sequences were removed from the reads using Trimmomatic [36] , resulting in 28 Gb of usable sequencing reads. The k-mer depth distribution of the cleaned high-quality sequences displayed a single peak (Figure 1), indicating that the *R. maidis* genome has a low level of heterozygosity.

Transcriptome sequencing

Transcriptome sequencing (Illumina strand-specific RNA-Seq and PacBio Iso-Seq) was conducted to aid gene prediction. Mixed-instar aphids feeding on barley were collected for total RNA extraction using the SV Total RNA isolation kit (Promega, Madison, WI, USA). Briefly, cells were lysed by grinding 100-120 mg of insect tissue in liquid nitrogen using a mortar and pestle, followed by incubation at 70°C in RNA lysis buffer (4M guanidine thiocyanate (GTC), 0.01M Tris, pH 7.5, 0.97% β-mercaptoethanol) for 3 min. This solution was then centrifuged for 10 min at 14,000 *g* and the supernatant was passed through a spin column provided with the kit, followed by DNase treatment. RNA was washed with RNA wash solution (60 mM potassium acetate, 10 mM Tris-

HCl (pH 7.5), 60% ethanol) and dissolved in 50 μl of nuclease-free water. Strand-specific RNA-Seq libraries were constructed using a previously described protocol [37] and sequenced at Biotechnology Resource Center of Cornell University (Ithaca, NY, USA) on an Illumina HiSeq 2500 sequencing system. More than 188 million paired-end reads with lengths of 151 bp were obtained (Supplementary Data S1). Raw reads were processed by trimming adaptor and low-quality sequences using Trimmomatic [36]. The cleaned reads were aligned to the assembled *R. maidis* genome using HISAT2 [38], followed by reference-guided assembly using StringTie [39]. The assembled transcripts were used to improve protein-coding gene predictions in the *R. maidis* genome.

For Iso-Seq, 20 μg RNA, isolated from 100-120 mg of fresh *R. maidis* tissue using the SV Total RNA isolation kit (Promega) with the method described above, was shipped to Duke Center for Genomic and Computational Biology (Durham, NC, USA) for PacBio large-insert library construction and sequencing using standard SMRTbell template preparation kits. The library insert size ranged from 500 bp up to 4,500 bp. One SMRT cell was run on the PacBio Sequel platform, yielding approximately 10 Gb raw sequence data (Supplementary Data S1). The PacBio raw reads were processed using IsoSeq3 (https://github.com/PacificBiosciences/IsoSeq3). Briefly, one representative Circular Consensus Sequence (CCS) was generated for each zero-mode waveguide (ZMW). Only ZMWs with at least one full pass, meaning that each primer has been seen at least once, were used for the subsequent analysis. The CCSs were processed to remove the 5' and 3' primers, trim off polyA tails and remove artificial concatemers to create full-length, non-concatemer (FLNC) reads. The FLNC reads were then clustered together. The final polishing step created a consensus sequence for each clustered transcript. A total of 21,114 high quality transcripts were generated and used to support protein-coding gene predictions in the *R. maidis* genome.

Hi-C library construction and sequencing

For Hi-C sequencing, 200 mg of *R. maidis* tissue was used for chromatin isolation and library preparation using the animal Hi-C kit from Phase Genomics (Seattle, WA, USA). Hi-C libraries were sequenced at the Biotechnology Resource Center at Cornell University (Ithaca, NY, USA) using the NextSeq500 platform (Illumina) to obtain 76 bp paired-end reads. Raw reads were processed by trimming adaptor and low-quality sequences using Trimmomatic [36]. The cleaned

Hi-C reads were aligned to the assembled contigs using BWA-aln [25], and the optimal placement of each read pair was determined by BWA-sampe [25]. Reads that did not map within 500 bp of a restriction enzyme site were removed using the PreprocessSAMs.pl script in LACHESIS [26]. Finally, only reads with mapping quality greater than 30 were used for scaffolding by LACHESIS [26].

Genome assembly

The PacBio long reads were corrected and assembled with the Canu assembler [40] (version 1.6). The resulting contigs were polished by aligning the raw PacBio reads to the assembly, and correcting the sequencing errors using Arrow (https://github.com/PacificBiosciences/GenomicConsensus). To further improve the assembly, another round of polishing was performed by aligning the Illumina short reads to the assembly and correcting the sequencing errors using Pilon [41].

The assembled contigs were then compared against the NCBI non-redundant nucleotide (nt) database using BLASTN with the parameters “-dust yes -max_target_seqs 10 -evalue 1e-5 -outfmt ‘6 qseqid sseqid pident length mismatch gapopen qstart qend sstart send evalue bitscore qlen slen sstrand staxids sscinames sskingdoms stitle’" to identify contamination based on taxonomy. This analysis identified sequences with homology to known sequences from archaea (*Methanobacterium formicicum*), bacteria (*Cronobacter dublinensis, Escherichia coli, Methylobacterium zatmanii, Planktothrix agardhii, Salmonella enterica*, and *Weissella cibaria*), eukaryotes (*Plasmodium falciparum, Brugia pahangi, Emplectanthus cordatus, Gossypium arboretum, Zea mays, Theobroma cacao, Frankliniella intonsa,* and *Oryzias latipes*), and plasmids. If >90% of an individual contig had likely non-aphid DNA, it was considered to be contamination. Altogether, we excluded 57 contigs, totaling 930,609 bp, from the assembly. Contigs were clustered by Phase Genomics Hi-C using LACHESIS [26] with default parameters. The resulting scaffolds were manually polished using Juicebox [42]. As Hi-C data do not provide the exact number of bp between the oriented contigs, Phase Genomics LACHESIS arbitrarily adds 100 Ns between contigs.

The assembled *R. maidis* genome, with a total length of 326.0 Mb, consisted of 689 contigs with an N50 length of 9.0 Mb. Thus, this is a much-improved genome assembly compared to the six previously published aphid genomes (Table 1). A total of 602 contigs spanning 323.4 Mb (99.2%

of the assembly) were clustered into four groups, which was consistent with the commonly observed 2n = 8 karyotype of *R. maidis* [22]. Of the clustered contigs, 473 spanning 321 Mb (98.4% of the assembly) were successfully orientated (Figure 2; Supplementary Figure S1). To evaluate the completeness of the genome assembly, it was aligned to Illumina paired-end libraries, allowing up to three mismatches using BWA-MEM [25]. With this approach, 94.2% of the Illumina reads could be mapped back to the assembly, indicating that most of the reads were successfully assembled into the genome. RNA-Seq reads were also aligned to the genome assembly using HISAT2 [38], resulting a mapping ratio of 94.1% (Supplementary Data S1). Furthermore, evaluation by BUSCO (v 3.0.2 [43], showed that 95.8% of the core eukaryotic genes were at least partially captured by the genome assembly and 94.5% were completely captured. The heterozygosity rate of the genome calculated by bbmap (https://jgi.doe.gov/data-and-tools/bbtools/bb-tools-user-guide/bbmap-guide/) is less than 0.00005, which is consistent with the profile of the k-mer distribution. The GC content of the genome is 27.7%, with 6.7% of total genome consisting of coding regions, 42.0% introns, and 51.2% intergenic regions. The 18,060 bp mitochondrial genome sequence was assembled separately (GenBank accession MK368778). Taken together, our evaluation indicated an overall high quality of the assembled *R. maidis* genome.

Aphids have an X0 sex determination system, with males receiving only one copy of the sex chromosome. Segregation of sex chromosomes in *Myzus persicae* and *Acyrthosiphon pisum* has been studied extensively using simple sequence repeat (SSR) markers [44-46]. We were not able to unambiguously assign the *R. maidis* X chromosome. Four *M. persicae* X *chromosome* markers [44] were assigned to three contigs on *R. maidis* chromosome 3. However, several *Ac. pisum* X chromosome markers [45, 46] were distributed across all four *R. maidis* chromosomes.

Endosymbiont genomes

The genome sequence of the *Buchnera aphidicola* endosymbiont was separated from the *R. maidis* host genome sequences by aligning the initial assembly to the *B. aphidicola* reference genome from *Ac. pisum* (*Buchnera*APS; GeneBank ID: NC_002528.1, [47]). One single contig was extracted and polished using both PacBio long reads and Illumina short reads, as described above. Genome annotation was performed using prokka [48]. The assembled *Buchnera*Rm genome had a length of 642,929 bp (Supplementary Figure S2), with 602 predicted protein-coding genes. The

two *Buchnera* plasmids, pLeu and pTrp, were also sequenced and assembled, with lengths of 7,852 bp and 3,674 bp, respectively (Supplementary Figure S2).

To identify secondary bacterial symbionts in *R. maidis*, raw assembled contigs were compared against the reference sequences of previously identified secondary bacterial symbionts of aphids, including *Hamiltonella defensa*, *Regiella insecticola*, *Serratia symbiotica*, *Rickettsia*, *Spiroplasma*, X-type, *Sitobion miscanthi* L-type, *Arsenophonus*, and *Wolbachia* [49], using BLAST with the parameters "-dust yes -max_target_seqs 10 -evalue 1e-5 -outfmt '6 qseqid sseqid pident length mismatch gapopen qstart qend sstart send evalue bitscore qlen slen sstrand staxids sscinames sskingdoms stitle'". Although no hits were found, we cannot determine whether this absence of secondary endosymbionts is specific to our *R. maidis* isolate or whether it is a more general property of this species. Whereas some studies have found secondary symbionts, including *S. symbiotica*, *S. miscanthi*, and *H. defensa* in *R. padi* [50], a closely related aphid species, others have not [51].

Annotation of repetitive elements

We identified MITE (miniature inverted-repeat transposable elements) from the assembled *R. maidis* genome using MITE-Hunter [52], and then generated a *de novo* repeat library by scanning the assembled genome using RepeatModeler (http://www.repeatmasker.org/RepeatModeler), which integrates results from RECON [53], TRF [54], and RepeatScout [55] and classifies repeats with the RepBase library [56]. RepeatModeler identified 546 repeats, which against the NCBI non-redundant (nr) protein database using BLAST with an e-value cutoff of 1e-5. Those having hits to known protein sequences were excluded. Finally, we identified repeat sequences by scanning the assembled *R. maidis* genome using the *de novo* repeat library with RepeatMasker (http://www.repeatmasker.org/) and the RepeatRunner subroutine (http://www.yandell-lab.org/software/repeatrunner.html) in the MAKER annotation pipeline [57]. A total of 21.2% of the assembled *R. maidis* genome was annotated as repeat elements (Table 2). The most predominant repeat elements were unknown repeats and MITEs, which occupied 5.6% and 4.4% of the genome respectively.

Gene prediction

Protein-coding genes were predicted from the genome assembly of *R. maidis* using the automated pipeline MAKER [57]. MAKER integrates the results from *ab initio* gene predictions with experimental gene evidence to produce final consensus gene set. The evidence that was used included complete aphid coding sequences collected from NCBI (https://www.ncbi.nlm.nih.gov/), transcripts assembled from our strand-specific RNA-Seq data, high quality transcript sequences from Iso-Seq, completed proteomes of *Ac. pisum, Aphis glycines, Diuraphis noxia, Myzus cerasi, M. persicae,* and *R. padi*, and proteins from the UniProt database. All of these sequences were aligned to the *R. maidis* genome using Spaln [58]. MAKER was used to run a battery of trained gene predictors, including Augustus [59], BRAKER [60] and GeneMark-ET [61], and then integrated the experimental gene evidence to produce evidence-based predictions. Altogether, 17,629 protein-coding genes were predicted for the four *R. maidis* chromosomes and non-scaffolded contigs (Table 3). The gene count of *R. maidis* is similar to that of *Ap. glycines* [33] and *M. persicae* [29], but lower than other aphid genome annotations (Table 1). However, it should be noted that estimated gene counts can vary depending on both the quality of the DNA sequencing and the specific annotation pipeline that is used. A recent comparative analysis of five aphid genomes using the same annotation pipeline identified similar total numbers of genes, ranging from 25,726 to 27,676 (Table 1) [32].

To functionally annotate the predicted genes, their protein sequences were compared against different protein databases including UnitProt (TrEMBL and SwissProt) and two insect proteomes (*Ac. pisum* and *Diaphorina citri*) using BLAST with an e-value cutoff of 1e-4. The protein sequences were also compared against the InterPro domain database [62]. GO annotation was performed with Blast2GO [63]. Among the 17,629 predicted *R. maidis* genes, 75.6% had hits to proteins in the Swiss-Prot or TrEMBL database, 36.0% were annotated with GO terms, 75.2% contained InterPro domains, 76.3% shared detectable homology with *Ac. pisum* genes, and 47.9% shared detectable homology with *D. citri* genes. Among the 4,248 genes (23.7% of the total) having no significant homology with the *Ac. pisum* genome, 4,026 were annotated as “unknown protein”. Only 41 of these genes have GO annotations.

Comparative genomics

We compared the *R. maidis* genes with those of six other aphid species (*Ap. glycines, M. persicae, Ac. pisum, M. cerasi, R. padi,* and *D. noxia*), as well as the whitefly (*Bemisia tabaci*) [27-33, 64].

The proteome sequences of all eight species were used to construct orthologous groups using OrthoMCL [65]. A total of 5,696 orthologous groups were shared by all 16 species, including 3,605 single-copy orthologous genes. Protein sequences of these single-copy genes were aligned with MUSCLE [66] and positions in the alignment containing gaps in more than 20% of the sequences were removed by trimAl [67]. A phylogenetic tree was constructed using the Maximum-Likelihood method implemented in PhyML [68], with the JTT model for amino acid substitutions and the aLRT method for branch support. *Bemisia tabaci* was used as the outgroup in the phylogenetic tree, which showed that *R. maidis* is close to *R. padi*, and separated from *Ac. pisum* and *M. persicae* (Figure 3), consistent with a phylogeny that was derived using mtCOI [69].

Identification of horizontal gene transfers

All of the *R. maidis* predicted gene models were compared against six protein databases derived from complete proteomes in UniProt, including those from bacteria, archaea, fungi, plants, metazoa (excluding proteins from other species in the Arthropoda), and other eukaryotes, using BLASTP. The index of horizontal gene transfer (HGT), h, was calculated by subtracting the bitscore of the best metazoan match from that of the best non-metazoan match [70]. We required that these sequences were aligned better to the other five taxa than to the metazoan database, defining HGT candidates as those with h ≥30 and a best non-metazoan hit bitscore ≥100. The corresponding genome sequences of these candidates as well as flanking aphid genes sequences at both ends were manually checked using IGV [71] (Supplementary Figures S3-S22). If there is any region with substantially reduced coverage, between HGTs and their flanking aphid genes, then this HGTs could be the result of incorrect assembly. Only HGTs in genomic regions with continuous read coverage were considered to be confirmed

We phylogenetically validated all HGT candidates. Their protein sequences were compared against the protein databases of six taxa (archaea, bacteria, fungi, plants, metazoan, and other eukaryotes) using BLASTP. The top five hits from each taxon were extracted, and aligned with the candidate HGT protein using ClustalW2 [72]. Each alignment was trimmed to exclude regions where gaps were more than 20% of sequences. Phylogenetic trees were constructed using PhyML [68] using a JTT model with 100 bootstraps (Supplementary Data S2-S21). A horizontally transferred gene was considered valid if the gene was monophyletic within the bacteria, archaea, fungi, plants, or protozoa. This analysis identified 20 HGTs, including two of bacterial origin,

seven of fungal origin, two from protozoa, and nine from algae (Table 4). The two bacterial genes were previously identified as horizontally transferred into *Ac. pisum* [73], and expression silencing of one of these genes, a bacteriocyte-expressed LD-carboxypeptidase A, was shown to reduce aphid performance [74]. A cluster of genes encoding multiple enzymes for carotenoid biosynthesis, which were horizontally transferred into the *Ac. pisum* genome from fungi [75], is also present in the *R. maidis* genome. Two *R. maidis* genes that cluster together with genes from trypanosomes and other protozoa have not been previously reported as horizontally transferred in aphids.

It is perhaps surprising that nine genes encoding proteins with ankyrin repeat domains show the highest similarity to genes from unicellular algae in the genus *Ostreococcus* and cluster with genes from this species in phylogenetic trees (Figure 4; Supplementary Data S11-S21). However, this does not necessarily mean that these genes were transferred from *Ostreococcus*, a type of picoplankton, but only that these are the most similar sequences available in UniProt. Three lines of evidence confirm that these are actual genes encoded in aphids: (i) the sequences are contiguous with other aphid genes in contigs assembled from PacBio long reads (Supplementary Figures S13-S22), (ii) RNA-Seq shows that the genes are transcribed in *R. maidis* (Supplementary Figure S23), and (iii) there are homologs of these genes in the other six published aphid genomes (*e.g.* XP_008185506.1 from *Ac. pisum*; Figure 4).

Detoxification and insecticide resistance

Cytochrome P450s, glutathione *S*-transferases (GSTs), carboxylesterases, UDP-glucosyltransferases (UGTs), and ABC transporters function in the avoidance and/or detoxification of plant defensive metabolites [76, 77], and insecticide resistance [78, 79]. We identified such detoxification-related genes in *R. maidis* based on protein domains that were predicted through InterProScan [80]. Cytochrome P450 genes were identified if their protein sequences contained the cytochrome P450 domain (InterPro ID: IPR001128). Genes with protein sequences containing the GST N-terminal and/or C-terminal domains (InterPro ID: IPR004045, IPR004046) were identified as GSTs. Carboxylesterases were identified on the basis of protein sequences that contained the carboxylesterase domain (InterPro domain ID: IPR002018) [81]. UDP-glucuronosyltransferases were identified if their protein sequences contained a UDP-glucuronosyl/UDP-glucosyltransferase domain (InterPro domain ID: IPR002213). ABC transporters were identified from the genome if their protein sequences contained an ABC

transporter-like domain (InterPro ID: IPR003439). Using the same approach, genes from these families were also identified in the other six aphid genomes (*Ap. glycines*, *M. persicae*, *Ac. pisum*, *M. cerasi*, *R. padi*, and *D. noxia*). The number of predicted detoxification genes in *R. maidis* is the lowest among the seven species that were examined (Table 5; Supplementary Data S30), consistent with *R. maidis* being a specialist monocot herbivore that may require a smaller repertoire of detoxification enzymes. Although the detoxification gene count in *Ac. pisum* was high, the average lengths of the protein sequences were shorter than those in *R. maidis*, *Ap. glycines*, and *M. persicae* (Supplementary Figure S24), suggesting that these genes could be incomplete or pseudogenes in *Ac. pisum*, possibly due to a lower-quality genome assembly.

Conclusion

As the currently most complete aphid genome, our *R. maidis* assembly will provide a valuable resource for comparisons with other species and the investigation of aphid genome evolution. Research on the ecological interactions of *R. maidis*, including host plant choices, detoxification of secondary metabolites, and gene expression responses, will be facilitated by the *R. maidis* genome sequence. Practical applications in agriculture may include the identification of virus transmission mechanisms and new targets for chemical pest control.

Availability of supporting data

This *R. maidis* Whole Genome Shotgun project has been deposited at DDBJ/ENA/GenBank under the accession QORX00000000. The version described in this paper is version QORX02000000. The *R. maidis* mitochondrial genome has been deposited in GenBank under accession MK368778. The *Buchnera aphidicola* Rm genome has been deposited in GenBank under accession CP032759. Raw genome and RNA-Seq sequences have been deposited in the NCBI Short Sequence Archive (SRA) under accession SRP164762. Genome sequence and annotation data are also available via the *GigaScience* database (GigaDB) records for this manuscript.

Additional Files

Figure S1. Hi-C contact map of the *R. maidis* genome.

Figure S2. Circular view of the genome of the *Rhopalosiphum maidis* endosymbiont, *Buchnera aphidicola* (A) and its plasmids pLeu (B) and pTrp (C).

Figure S3-S22. PacBio read alignments around the HGT genes

Figure S23. RNA-Seq reads alignments around the Rma14036 HGT gene

Figure S24. Length distribution of protein sequences of detoxification gene families in seven aphid species.

Data S1. Summary of PacBio long reads and Illumina short reads

Data S2-S21. Phylogenetic tree files for testing candidate HGT

Data S22. Predicted detoxification genes in *R. maidis.*

Abbreviations

CCS: circular consensus sequence

ZMW: zero-mode waveguide

FLNC: full-length, non-concatemer reads

nt: nucleotide

HGT: horizontal gene transfer

GST: glutathione S-transferase

CCE: carboxylesterase

UGT: UDP-glucuronosyltransferases

Author contributions

GJ and ZF conceived of the research, SS, MB, and AR raised aphids and isolated nucleic acids, WC conducted data analysis, and WC and GJ wrote the manuscript.

Ethics approval and consent to participate

This is not required for experiments with *R. maidis.*

Competing interests

The authors have no competing financial and non-financial competing interests.

Funding

This research was sponsored by the Defense Advanced Research Projects Agency (DARPA) and was accomplished under cooperative agreement number HR0011-17-2-0053. The views and conclusions contained in this document are those of the authors and should not be interpreted as representing the official policies, either expressed or implied, of DARPA or the U.S. Government. The U.S. Government is authorized to reproduce and distribute reprints for Government purposes notwithstanding any copyright notation hereon.

References

1. Meihls LN, Kaur H and Jander G. Natural variation in maize defense against insect herbivores. Cold Spring Harbor Symp Quant Biol. 2012;77:269-83.
2. McMullen M, Frey M and Degenhardt J. Genetics and biochemistry of insect resistance in maize. In: Bennetzen JL and Hake S, editors. Handbook of Maize: its Biology. New York: Springer; 2009. p. 587.
3. Machado S, Bynum ED, Archer TL, Lascano RJ, Wilson LT, Bordovsky J, et al. Spatial and temporal variability of corn growth and grain yield: Implications for site-specific farming. Crop Science. 2002;42 5:1564-76.
4. Blackman RL and Eastop VF. Aphids on the World's Crops. Chichester: Wiley; 2000.
5. Caballero PP, Ramirez CC and Niemeyer HM. Specialisation pattern of the aphid *Rhopalosiphum maidis* is not modified by experience on a novel host. Ent Exp Appl. 2001;100 1:43-52.
6. Meihls LN, Handrick V, Glauser G, Barbier H, Kaur H, Haribal MM, et al. Natural variation in maize aphid resistance is associated with 2,4-dihydroxy-7-methoxy-1,4-benzoxazin-3-one glucoside methyltransferase activity. Plant Cell. 2013;25 6:2341-55.
7. Betsiashvili M, Ahern KR and Jander G. Additive effects of two quantitative trait loci that confer *Rhopalosiphum maidis* (corn leaf aphid) resistance in maize inbred line Mo17. J Exp Bot. 2015;66:571-8.
8. Bing JW, Guthrie WD, Dicke FF and Obrycki JJ. Seedling stage feeding by corn leaf aphid (Homoptera, Aphididae) - Influence on plant development in maize. J Econ Ent. 1991;84 2:625-32.
9. Carena MJ and Glogoza P. Resistance of maize to the corn leaf aphid: A review. Maydica. 2004;49:241-54.
10. Foott WH and Timmins PR. Effects of infestations by corn leaf aphid, *Rhopalosiphum maidis* (Homoptera-Aphididae), on field corn in southwestern Ontario. Can Ent. 1973;105 3:449-58.
11. El-Muadhidi MA, Makkouk KM, Kumari SG, Myasser J, Murad SS and Mustafa R. Survey for legume and cereal viruses in Iraq. Phytopathol Mediterr. 2001;40:224-3.

12. Hawkes JR and Jones RAC. Incidence and distribution of Barley yellow dwarf virus and Cereal yellow dwarf virus in over-summering grasses in a Mediterranean-type environment. Aust J Ag Res. 2005;56 3:257-70.

13. Jarošová J, Chrpová J, Šíp V and Kundu JK. A comparative study of the *Barley yellow dwarf virus* species PAV and PAS: distribution, accumulation and host resistance. Plant Pathol. 2013;62 2:436-43.

14. Power AG, Borer ET, Hosseini P, Mitchell CE and Seabloom EW. The community ecology of barley/cereal yellow dwarf viruses in Western US grasslands. Virus Res. 2011;159 2:95-100.

15. Krueger EN, Beckett RJ, Gray SM and Miller WA. The complete nucleotide sequence of the genome of *Barley yellow dwarf virus*-RMV reveals it to be a new Polerovirus distantly related to other yellow dwarf viruses. Front Microbiol. 2013;4

16. El-Ibrashy MT, El-Ziady S and Riad AA. Laboratory studies on the biology of the corn leaf aphid, *Rhopalosiphum maidis* (Homoptera: Aphididae). Ent Exp Appl. 1972;15:166-74.

17. Gill CC and Metcalfe DR. Resistance in barley to the corn leaf aphid *Rhopalosiphum maidis*. Can J Plant Sci. 1977;57:1063-70.

18. Lee S, Holman J and Havelka J. Illustrated Catalogue of Aphididae in the Korean Peninsula Part I, Subfamily Aphidinae. Deajon, Korea: Korea Research Institute of Bioscience and Biotechnology; 2002.

19. Remaudière G and Naumann-Etienne K. Découverte au Pakistan de l'hôte primaire de *Rhopalosiphum maidis*. C R Acad Agric Fr 1991;77:61-2.

20. Torikura H. Revisional notes on Japanese *Rhopalosiphum*, with keys to species based on the morphs on the primary host. Jap J Ent. 1991;59:257-73.

21. Eastop VF. The males of *Rhopalosiphum maidis* (Fitch) and the discussion of the use of males in aphid taxonomy. Proc Roy Ent Soc Lond (A). 1954;29:84-6.

22. Brown PA and Blackman RL. Karyotype variation in the corn leaf aphid, *Rhopalosiphum maidis* (Fitch), species complex (Hemiptera, Aphididae) in relation to host-plant and morphology. Bull Ent Res. 1988;78 2:351-63.

23. Blackman RA and Brown PA. Morphometric variation within and between populations of *Rhopalosiphum maidis* with a discussion of the taxonomic treatment of permanently parthenogenetic aphids (Homoptera: Aphididae). Ent Gen. 1991;16:97-113.

24. Quail MA, Smith M, Coupland P, Otto TD, Harris SR, Connor TR, et al. A tale of three next generation sequencing platforms: comparison of Ion Torrent, Pacific Biosciences and Illumina MiSeq sequencers. BMC Genomics. 2012;13:341.

25. Li H and Durbin R. Fast and accurate short read alignment with Burrows-Wheeler transform. Bioinformatics. 2009;25 14:1754-60.

26. Burton JN, Adey A, Patwardhan RP, Qiu R, Kitzman JO and Shendure J. Chromosome-scale scaffolding of *de novo* genome assemblies based on chromatin interactions. Nat Biotechnol. 2013;31 12:1119-25.

27. Richards S, Gibbs RA, Gerardo NM, Moran N, Nakabachi A, Stern D, et al. Genome sequence of the pea aphid *Acyrthosiphon pisum*. PLoS Biology. 2010;8 2:e1000313.

28. Thorpe P, Cock PJ and Bos J. Comparative transcriptomics and proteomics of three different aphid species identifies core and diverse effector sets. BMC Genomics. 2016;17:172.

29. Mathers TC, Chen Y, Kathakottil G, Legeai F, Mugford ST, Baa-Puolet P, et al. Rapid transcriptional plasticity of duplicated gene clusters enables a clonally reproducing aphid to colonize diverse plant species. Genome Biol. 2017;18:27.

30. Burger NFV and Botha AM. Genome of Russian wheat aphid an economically important cereal aphid. Stand Genomic Sci. 2017;12:90.

31. Nicholson SJ, Nickerson ML, Dean M, Song Y, Hoyt PR, Rhee H, et al. The genome of *Diuraphis noxia*, a global aphid pest of small grains. BMC Genomics. 2015;16:429.

32. Thorpe P, Escudero-Martinez CM, Cock PJA, Eves-van den Akker S and Bos JIB. Shared transcriptional control and disparate gain and loss of aphid parasitism genes. Genome Biol Evol. 2018;10 10:2716-33.

33. Wenger JA, Cassone BJ, Legeai F, Johnston JS, Bansal R, Yates AD, et al. Whole genome sequence of the soybean aphid, *Aphis glycines*. Insect Biochem Mol Biol. 2017;epub ahead of print, doi: S0965-1748(17)30005-X [pii] 10.1016/j.ibmb.2017.01.005

34. Fulton M, Chunwongse J and Tanksley SD. Microprep protocol for extraction of DNA from tomato and other herbaceous plants. Plant Mol Biol Rep. 1995;13:207-9.

35. Chen W, Hasegawa DK, Arumuganathan K, Simmons AM, Wintermantel WM, Fei Z, et al. Estimation of the whitefly *Bemisia tabaci* genome size based on k-mer and flow cytometric analyses. Insects. 2015;6 3:704-15.

36. Bolger AM, Lohse M and Usadel B. Trimmomatic: A flexible trimmer for Illumina sequence data. Bioinformatics. 2014;30 15:2114-20.

37. Zhong S, Joung JG, Zheng Y, Chen YR, Liu B, Shao Y, et al. High-throughput Illumina strand-specific RNA sequencing library preparation. Cold Spring Harb Protoc. 2011;2011 8:940-9.

38. Kim D, Langmead B and Salzberg SL. HISAT: A fast spliced aligner with low memory requirements. Nat Methods. 2015;12 4:357-60.

39. Pertea M, Pertea GM, Antonescu CM, Chang TC, Mendell JT and Salzberg SL. StringTie enables improved reconstruction of a transcriptome from RNA-seq reads. Nat Biotechnol. 2015;33 3:290-5.

40. Koren S, Walenz BP, Berlin K, Miller JR, Bergman NH and Phillippy AM. Canu: scalable and accurate long-read assembly via adaptive k-mer weighting and repeat separation. Genome Res. 2017;27 5:722-36.

41. Walker BJ, Abeel T, Shea T, Priest M, Abouelliel A, Sakthikumar S, et al. Pilon: An integrated tool for comprehensive microbial variant detection and genome assembly improvement. PloS One. 2014;9 11:e112963.

42. Durand NC, Robinson JT, Shamim MS, Machol I, Mesirov JP, Lander ES, et al. Juicebox provides a visualization system for Hi-C contact maps with unlimited zoom. Cell Syst. 2016;3 1:99-101.

43. Simao FA, Waterhouse RM, Ioannidis P, Kriventseva EV and Zdobnov EM. BUSCO: Assessing genome assembly and annotation completeness with single-copy orthologs. Bioinformatics. 2015;31 19:3210-2.

44. Wilson AC, Delgado RN and Vorburger C. Biased transmission of sex chromosomes in the aphid *Myzus persicae* is not associated with reproductive mode. PLoS One. 2014;9 12:e116348.

45. Jaquiery J, Rispe C, Roze D, Legeai F, Le Trionnaire G, Stoeckel S, et al. Masculinization of the x chromosome in the pea aphid. PLoS Genet. 2013;9 8:e1003690.

46. Jaquiery J, Peccoud J, Ouisse T, Legeai F, Prunier-Leterme N, Gouin A, et al. Disentangling the causes for faster-X evolution in aphids. Genome Biol Evol. 2018;10 2:507-20.
47. Shigenobu S, Watanabe H, Hattori M, Sakaki Y and Ishikawa H. Genome sequence of the endocellular bacterial symbiont of aphids *Buchnera* sp. APS. Nature. 2000;407 6800:81-6.
48. Seemann T. Prokka: Rapid prokaryotic genome annotation. Bioinformatics. 2014;30 14:2068-9.
49. Zytynska SE and Weisser WW. The natural occurrence of secondary bacterial symbionts in aphids. Ecol Ent. 2015;41:13-26.
50. Leybourne DJ, Bos JIB, Valentine TA and Karley AJ. The price of protection: a defensive endosymbiont impairs nymph growth in the bird cherry-oat aphid, *Rhopalosiphum padi*. Insect Sci. 2018;epub ahead of print, DOI: 10.1111/1744-7917.12606
51. Zepeda-Paulo F, Ortiz-Martinez S, Silva AX and Lavandero B. Low bacterial community diversity in two introduced aphid pests revealed with 16S rRNA amplicon sequencing. PeerJ. 2018;6:e4725.
52. Han Y and Wessler SR. MITE-Hunter: a program for discovering miniature inverted-repeat transposable elements from genomic sequences. Nucleic Acids Res. 2010;38 22:e199-e.
53. Bao Z and Eddy SR. Automated de novo identification of repeat sequence families in sequenced genomes. Genome Res. 2002;12 8:1269-76.
54. Benson G. Tandem repeats finder: A program to analyze DNA sequences. Nucleic Acids Res. 1999;27 2:573-80.
55. Price AL, Jones NC and Pevzner PA. De novo identification of repeat families in large genomes. Bioinformatics. 2005;21 Suppl 1:i351-8.
56. Bao W, Kojima KK and Kohany O. Repbase Update, a database of repetitive elements in eukaryotic genomes. Mobile DNA. 2015;6 1:11.
57. Cantarel BL, Korf I, Robb SM, Parra G, Ross E, Moore B, et al. MAKER: An easy-to-use annotation pipeline designed for emerging model organism genomes. Genome Res. 2008;18 1:188-96.
58. Gotoh O. Direct mapping and alignment of protein sequences onto genomic sequence. Bioinformatics. 2008;24 21:2438-44.

59. Stanke M and Waack S. Gene prediction with a hidden Markov model and a new intron submodel. Bioinformatics. 2003;19 Suppl 2:ii215-25.

60. Hoff KJ, Lange S, Lomsadze A, Borodovsky M and Stanke M. BRAKER1: Unsupervised RNA-Seq-based genome annotation with GeneMark-ET and AUGUSTUS. Bioinformatics. 2015;

61. Lomsadze A, Burns PD and Borodovsky M. Integration of mapped RNA-Seq reads into automatic training of eukaryotic gene finding algorithm. Nucleic Acids Res. 2014;42 15:e119.

62. Mitchell A, Chang HY, Daugherty L, Fraser M, Hunter S, Lopez R, et al. The InterPro protein families database: The classification resource after 15 years. Nucleic Acids Res. 2015;43 Database issue:D213-21.

63. Conesa A, Gotz S, Garcia-Gomez JM, Terol J, Talon M and Robles M. Blast2GO: A universal tool for annotation, visualization and analysis in functional genomics research. Bioinformatics. 2005;21 18:3674-6.

64. Chen W, Hasegawa DK, Kaur N, Kliot A, Pinheiro PV, Luan J, et al. The draft genome of whitefly *Bemisia tabaci* MEAM1, a global crop pest, provides novel insights into virus transmission, host adaptation, and insecticide resistance. BMC Biol. 2016;14 1:110.

65. Li L, Stoeckert CJ, Jr. and Roos DS. OrthoMCL: Identification of ortholog groups for eukaryotic genomes. Genome Res. 2003;13 9:2178-89.

66. Edgar RC. MUSCLE: Multiple sequence alignment with high accuracy and high throughput. Nucleic Acids Res. 2004;32 5:1792-7.

67. Capella-Gutierrez S, Silla-Martinez JM and Gabaldon T. TrimAl: A tool for automated alignment trimming in large-scale phylogenetic analyses. Bioinformatics. 2009;25 15:1972-3.

68. Guindon S, Delsuc F, Dufayard JF and Gascuel O. Estimating maximum likelihood phylogenies with PhyML. Methods Mol Biol. 2009;537:113-37.

69. Papasotiropoulos V, Tsiamis G, Papaioannou C, Ioannidis P, Klossa-Kilia E, Papapanagiotou AP, et al. A molecular phylogenetic study of aphids (Hemiptera: Aphididae) based on mitochondrial DNA sequence analysis. J Biol Res. 2013;20:195.

70. Crisp A, Boschetti C, Perry M, Tunnacliffe A and Micklem G. Expression of multiple horizontally acquired genes is a hallmark of both vertebrate and invertebrate genomes. Genome Biol. 2015;16:50.

71. Robinson JT, Thorvaldsdottir H, Winckler W, Guttman M, Lander ES, Getz G, et al. Integrative genomics viewer. Nat Biotechnol. 2011;29 1:24-6.

72. Larkin MA, Blackshields G, Brown NP, Chenna R, McGettigan PA, McWilliam H, et al. Clustal W and Clustal X version 2.0. Bioinformatics. 2007;23 21:2947-8.

73. Nikoh N, McCutcheon JP, Kudo T, Miyagishima SY, Moran NA and Nakabachi A. Bacterial genes in the aphid genome: absence of functional gene transfer from *Buchnera* to its host. PLoS Genet. 2010;6 2:e1000827.

74. Chung SH, Jing X, Luo Y and Douglas AE. Targeting symbiosis-related insect genes by RNAi in the pea aphid-*Buchnera* symbiosis. Insect Biochem Mol Biol. 2018;95:55-63.

75. Moran NA and Jarvik T. Lateral transfer of genes from fungi underlies carotenoid production in aphids. Science. 2010;328 5978:624-7.

76. Woldman Y and Appling DR. A general method for determining the contribution of split pathways in metabolite production in the yeast *Saccharomyces cerevisiae*. Metab Eng. 2002;4 2:170-81.

77. Field LM. Methylation and expression of amplified esterase genes in the aphid *Myzus persicae* (Sulzer). Biochem J. 2000;349:863-8.

78. Huang FF, Chai CL, Zhang Z, Liu ZH, Dai FY, Lu C, et al. The UDP-glucosyltransferase multigene family in *Bombyx mori*. BMC genomics. 2008;9:563.

79. Dermauw W and Van Leeuwen T. The ABC gene family in arthropods: comparative genomics and role in insecticide transport and resistance. Insect Biochem Mol Biol. 2014;45:89-110.

80. Jones P, Binns D, Chang HY, Fraser M, Li W, McAnulla C, et al. InterProScan 5: Genome-scale protein function classification. Bioinformatics. 2014;30 9:1236-40.

81. Oakeshott JG, Claudianos C, Campbell PM, Newcomb RD and Russell RJ. Biochemical genetics and genomics of insect esterases. In: Gilbert LI and Gill SS, editors. Insect Pharmacology: Channels, Receptors, Toxins and Enzyme. Amsterdam: Academic Press; 2010. p. 392.

Table 1. Assembly statistics of seven aphid genomes.

Species	*R. maidis*	*Ap. glycines*	*M. persicae**	*Ac. pisum*	*M. cerasi*	*R. padi*	*D. noxia**
Sequencing reference #	[this study]	[33]	[29]	[27]	[28]	[28]	[31]
Genome assembly							
Assembly size (Mb)	326.0	302.9	347.3	541.6	405.7	319.4	393.0
Contig Count	689	66,000	8,249	60,623	56,508	16,689	49,357
Contig N50 (bp)	9,046,396	15,844	144,275	28,192	17,908	96,831	12,578
Scaffold Count	220	8,397	4,022	23,924	49,286	15,587	5,641
Scaffold N50 (bp)	93,298,903	174,505	435,781	518,546	23,273	116,185	397,774
Max. scaffold length (Mb)	94.2	1.4	2.2	3	0.2	0.6	2.1
Min. scaffold length (kb)	1.1	2	0.9	0.2	1	1	0.9
Genomic features							
Transcript length (bp)	1,834.6	1,520.1	1,838.7	1,964.1	NA	NA	NA
CDS length (bp)	1,242.04	1,240.3	1,328.3	1,157.6	952.7	1155.09	970.2
exon length (bp)	210.02	245.5	299.2	394.7	NA	NA	NA
exon count/gene	6.31	6.19	6.14	4.97	NA	NA	NA
Gene counts [reference #]	17,629 [this study]	19,182 [33]	18,529 [29]<br>25,726 [32]<br>18,433 [29]<br>23,822 [28]<br>24,742 [28]<br>21,441 [28]	36,195 [27]<br>27,676 [32]	28,408 [28]<br>28,688 [32]	28,542 [28]<br>26,286 [32]	19,097 [31]<br>25,987 [32]<br>31,885 [30]

NA: This information could not be retrieved from the annotation files.
*More than one sequenced lineage

Table 2. Repeats in the *R. maidis* genome assembly

Class	No. of Copies	Length (bp)	Coverage of genome (%)
SINE	27,308	7,085,803	2.17
LINE	6,688	1,596,259	0.49
LTR	3,445	896,470	0.28
DNA transposon	53,797	9,499,710	2.91
MITE	64,663	14,240,430	4.37
Unclassified	49,627	18,375,079	5.64
Other*	375,149	17,360,944	5.33
Total	580,677	69,054,695	21.18

*Other: microsatellites/simple repeats/low complexity sequences

Table 3. Gene distribution on the *R. maidis* chromosomes

Chromosome	Length (bp)	Gene count
**Chr1**	94,224,415	4,968
**Chr2**	93,298,903	5,124
**Chr3**	76,887,858	4,478
**Chr4**	56,292,413	3,034
**Not assigned**	5,319,566	25
**Total**	326,023,155	17,629

Table 4. Horizontally transferred genes in *R. maidis*

Gene ID	Function Description	Possible Origin
Rma07998	Peptidase U61; LD-carboxypeptidase A	Bacteria
Rma09603	Carbamoylphosphate synthase large subunit	Bacteria
Rma01752	Lycopene cyclase phytoene synthase	Fungi
Rma01753	Carotenoid desaturase	Fungi
Rma01754	Lycopene cyclase phytoene synthase	Fungi
Rma01756	Lycopene cyclase phytoene synthase	Fungi
Rma01758	Lycopene cyclase phytoene synthase	Fungi
Rma01759	Lycopene cyclase phytoene synthase	Fungi
Rma01760	Carotenoid desaturase	Fungi
Rma08772	Leucine Rich Repeat family protein	Protozoa
Rma11572	Antigenic protein, putative	Protozoa
Rma10344	Ankyrin repeat protein	Algae
Rma11418	Ankyrin repeat protein	Algae
Rma12243	Ankyrin repeat protein	Algae
Rma13322	Ankyrin repeat protein	Algae
Rma13584	Ankyrin repeat protein	Algae
Rma14036	Ankyrin repeat protein	Algae
Rma15269	Ankyrin repeat protein	Algae
Rma16213	Ankyrin repeat protein	Algae
Rma16838	Ankyrin repeat protein	Algae

Table 5. Numbers of predicted detoxification genes in seven aphid species

	R. maidis	*Ap. glycines*	*M. persicae*	*Ac. pisum*	*M. cerasi*	*R. padi*	*D. noxia*
Cytochrome P450s	59	61	67	82	74	67	60
Glutathione S-transferases	10	12	13	36	12	11	11
Carboxylesterases	23	31	37	48	36	34	32
UDP-glucuronosyltransferases	43	47	57	72	48	55	43
ABC transporters	68	74	67	126	68	71	63
Total	203	225	241	364	238	238	209

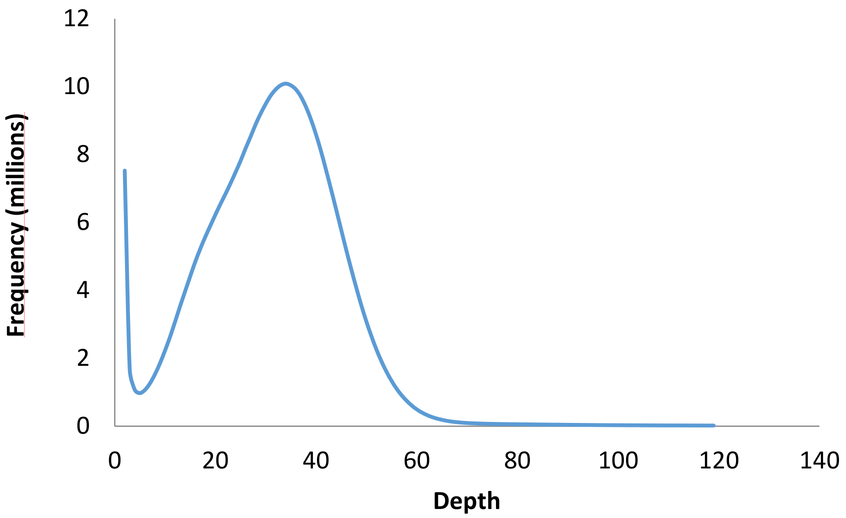

Figure 1. K-mer (K=31) distribution of Illumina genome sequencing reads of *R. maidis*. The total count of k-mers was 11,495,021,417, and the peak of k-mer depth was 34. The genome size of *R. maidis* was calculated by dividing the total k-mer count by the peak depth, which was approximately 338 Mb. The single peak of k-mer distribution profile indicates that the *R. maidis* genome has a low level of heterozygosity.

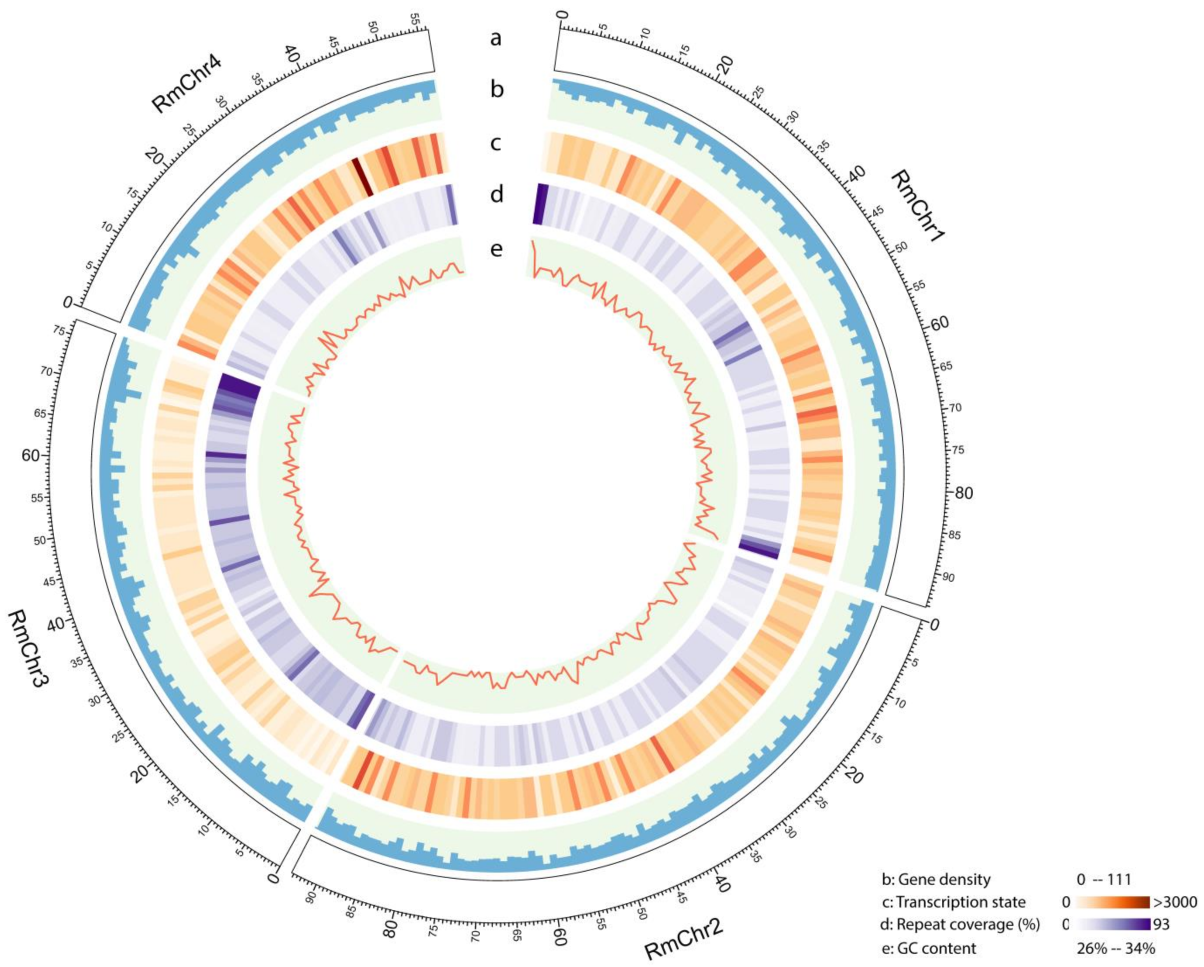

Figure 2. *Rhopalosiphum maidis* genome landscape. (a) Ideogram of the four *R. maidis* pseudochromosomes at the Mb scale. (b) Gene density represented as number of genes per Mb. (c) Transcription state. The transcription level was estimated by read counts per million mapped reads in 1-Mb windows. (d) Percentage of coverage of repeat sequences per Mb. (e) GC content in 1-Mb windows. The four *R. maidis* pseudo-chromosomes represented 99.3% of the genome assembly. This figure was generated using Circos (http://circos.ca/).

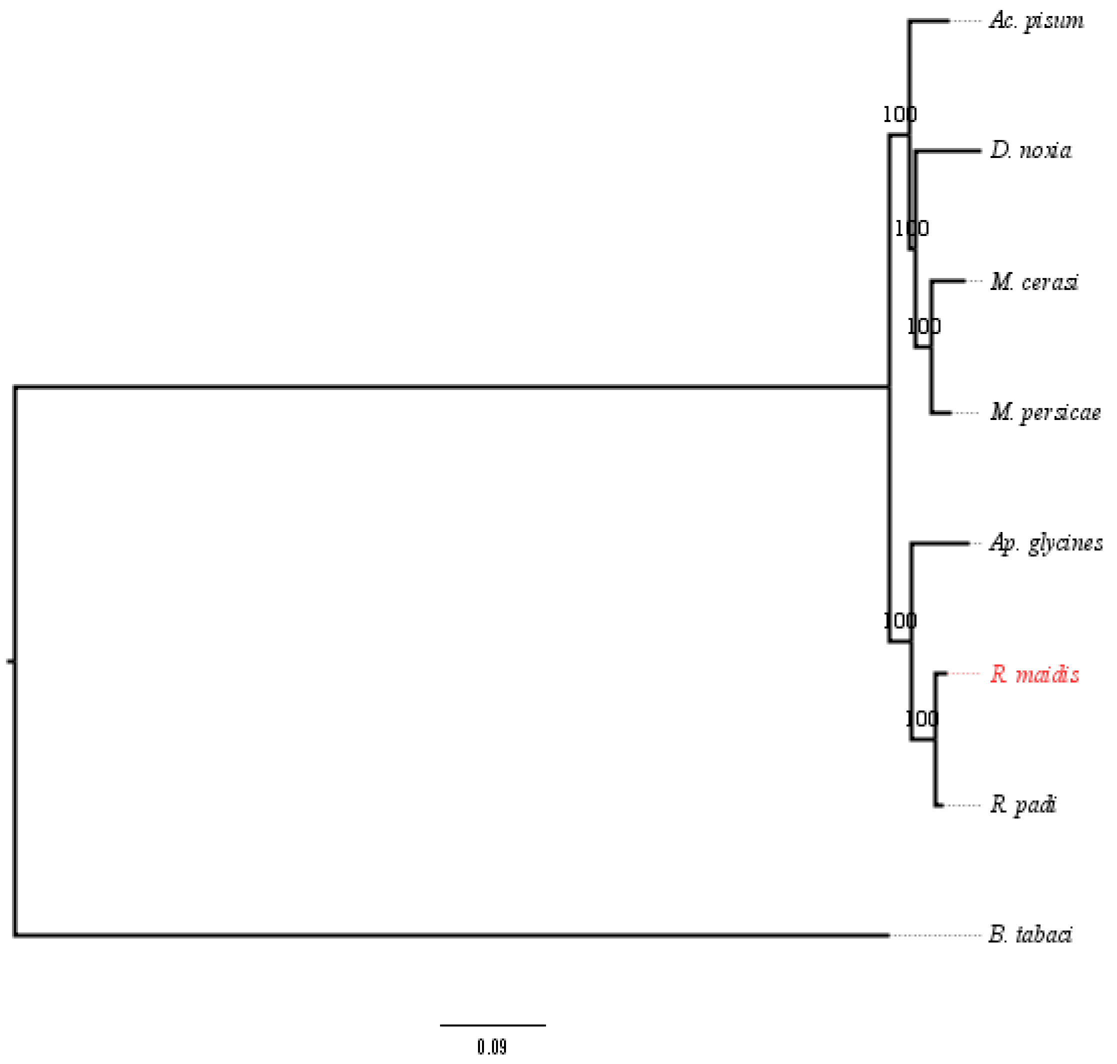

Figure 3. Phylogenetic relationships of *R. maidis* and 7 other arthropod species. *B. tabaci* was used as the outgroup taxon.

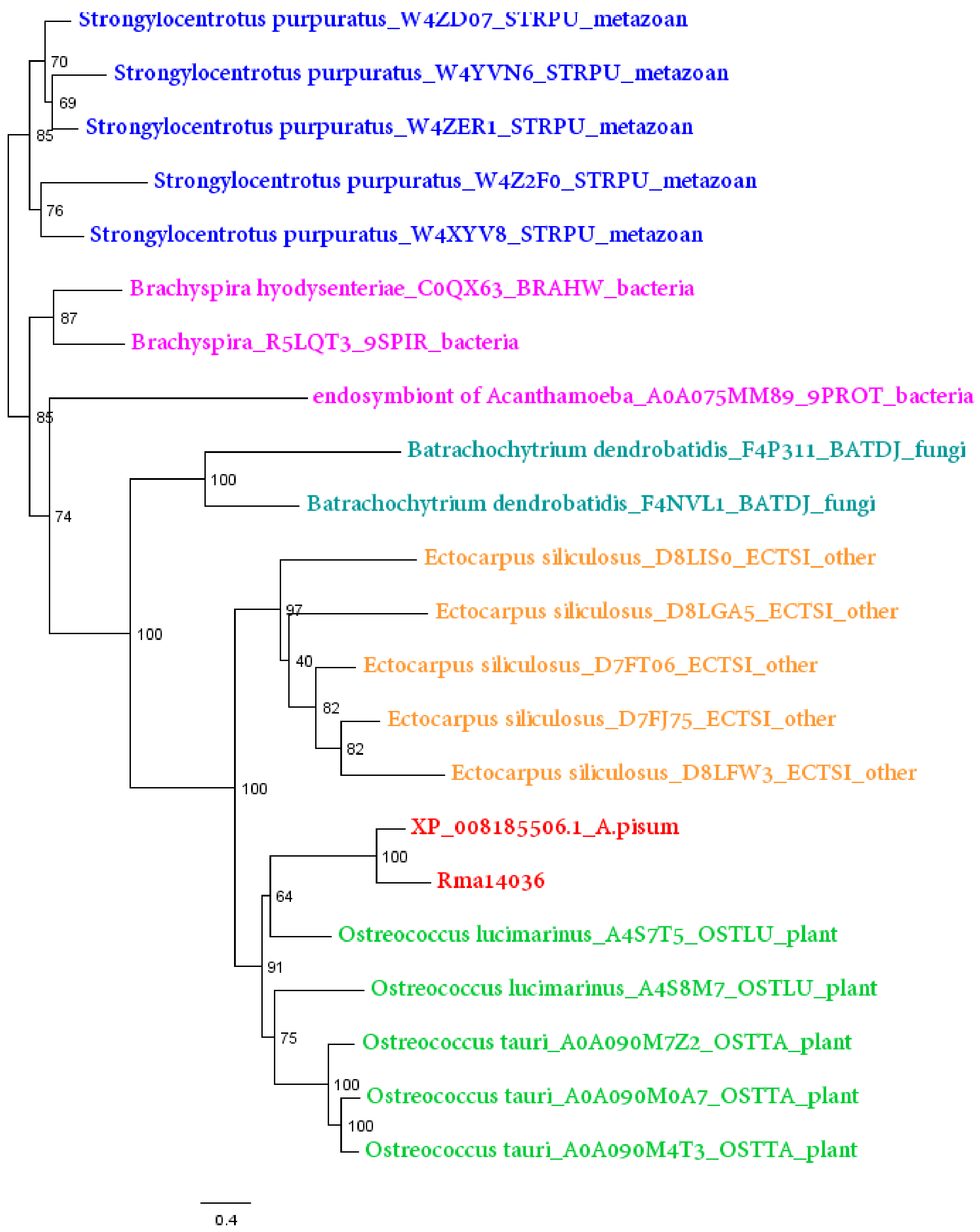

Figure 4. A family of aphid proteins (examples of Rma14036 from *R. maidis* and XP_008185506.1 from *Ac. pisum* are shown in red) cluster most closely with proteins from *Ostreococcus* algae.

Click here to access/download
Supplementary Material
Supplementary Figures S1-S24.docx

Click here to access/download
Supplementary Material
Supplementary Data S1 - PacBio and Illumina summary.xlsx

Supplementary Data S2, phylogenetic tree file

Click here to access/download

Supplementary Material

Supplementary Data S2 - Rma07998.trim_phyml_tree

Supplementary Data S3, phylogenetic tree file

Click here to access/download

Supplementary Material

Supplementary Data S3 - Rma09603.trim_phyml_tree

Supplementary Data S4, phylogenetic tree file

Click here to access/download
Supplementary Material
Supplementary Data S4 - Rma01752.trim_phyml_tree

Supplementary Data S5, phylogenetic tree file

Click here to access/download
Supplementary Material
Supplementary Data S5 - Rma01754.trim_phyml_tree

Supplementary Data S6, phylogenetic tree file

Click here to access/download

Supplementary Material

Supplementary Data S6 - Rma01753.trim_phyml_tree

Supplementary Data S7, phylogenetic tree file

Click here to access/download

Supplementary Material

Supplementary Data S7 - Rma01756.trim_phyml_tree

Supplementary Data S8, phylogenetic tree file

Click here to access/download

Supplementary Material

Supplementary Data S8 - Rma01758.trim_phyml_tree

Supplementary Data S9, phylogenetic tree file

Click here to access/download
Supplementary Material
Supplementary Data S9 - Rma01759.trim_phyml_tree

Supplementary Data S10, phylogenetic tree file

Click here to access/download
Supplementary Material
Supplementary Data S10 - Rma01760.trim_phyml_tree

Supplementary Data S11, phylogenetic tree file

Click here to access/download
Supplementary Material
Supplementary Data S11 - Rma08772.trim_phyml_tree

Supplementary Data S12, phylogenetic tree file

Click here to access/download
Supplementary Material
Supplementary Data S12 - Rma11572.trim_phyml_tree

Supplementary Data S13, phylogenetic tree file

Click here to access/download

Supplementary Material

Supplementary Data S13 - Rma10344.trim_phyml_tree

Supplementary Data S14, phylogenetic tree file

Click here to access/download

Supplementary Material

Supplementary Data S14 - Rma11418.trim_phyml_tree

Supplementary Data S15, phylogenetic tree file

Click here to access/download

Supplementary Material

Supplementary Data S15 - Rma12243.trim_phyml_tree

Supplementary Data S16, phylogenetic tree file

Click here to access/download
Supplementary Material
Supplementary Data S16 - Rma13322.trim_phyml_tree

Supplementary Data S18, phylogenetic tree file

Click here to access/download
Supplementary Material
Supplementary Data S18 - Rma14036.trim_phyml_tree

Supplementary Data S19, phylogenetic tree file

Click here to access/download
Supplementary Material
Supplementary Data S19 - Rma15269.trim_phyml_tree

Supplementary Data S20, phylogenetic tree file

Click here to access/download

Supplementary Material

Supplementary Data S20 - Rma16213.trim_phyml_tree

Supplementary Data S21, phylogenetic tree file

Click here to access/download

Supplementary Material

Supplementary Data S21 - Rma16838.trim_phyml_tree

Click here to access/download
Supplementary Material
Supplementary Data S22 - predicted detoxification genes.xlsx

Personal Cover

Click here to access/download;Personal Cover;R maidis genome re-revision cover letter 2-7-18.pdf

Georg Jander
Boyce Thompson Institute
533 Tower Road
Ithaca, NY 14853
USA

email: gj32@cornell.edu
phone: (607) 254-1365

2/7/2019

Dear *GigaScience* Editors,

We are hereby re-submitting our manuscript, ”Genome sequence of the corn leaf aphid (*Rhopalosiphum maidis* Fitch)”, to your consideration for publication in *GigaScience*.

As detailed in the responses to reviewers, we have made corrections to the manuscript. The change that we have made in response to the reviewer comment is highlighted in red in the manuscript text.

The authors do not have any competing interests. All authors have approved the manuscript for submission. The manuscript has not been submitted for publication elsewhere, but a preprint has been submitted to *biorXiv*. We hope that this revised manuscript will be suitable for publication in *GigaScience.*

Sincerely,

Georg Jander
