# Supplementary material for: Genome sequence of the corn leaf aphid (Rhopalosiphum maidis Fitch)
Source: Gigascience. 2019 Apr 6;8(4):giz033. doi: 10.1093/gigascience/giz033 (PMC6451198; doi:10.1093/gigascience/giz033)
Supplement: Supplemental Files [file giz033_supplemental_files.zip › Supplementary Figures S1-S24.docx]

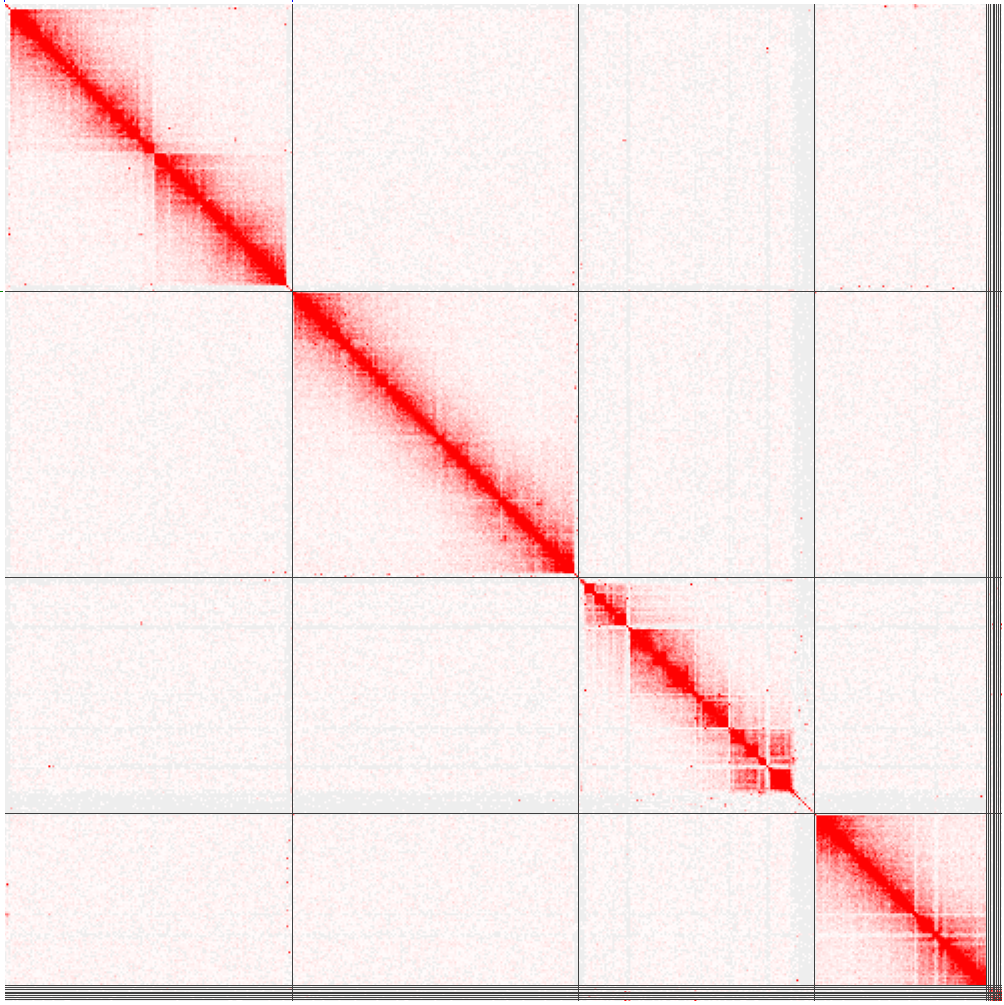


**Figure S1**. Hi-C contact map of the *R. maidis* genome

**A.**


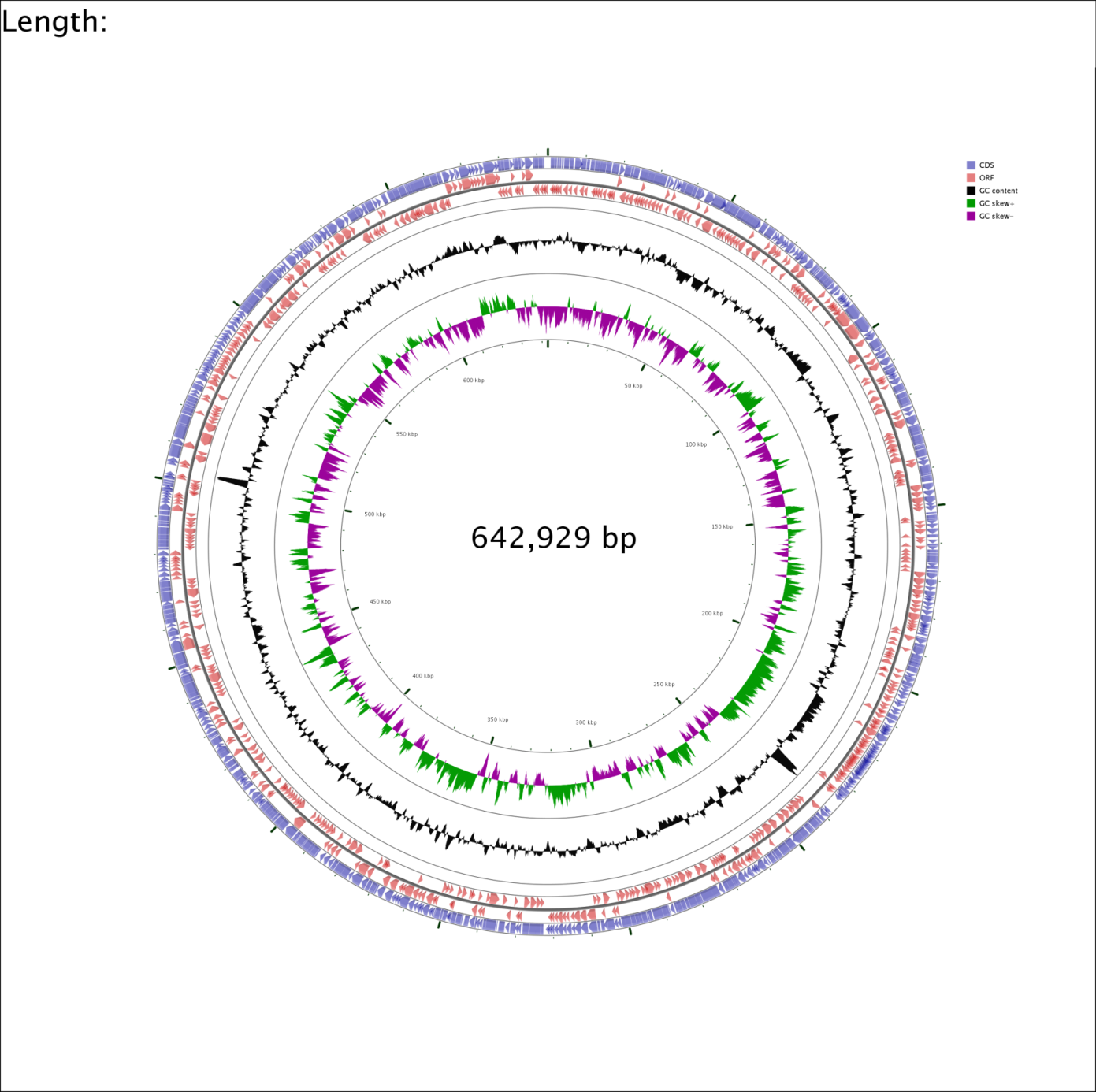


**B. C.**


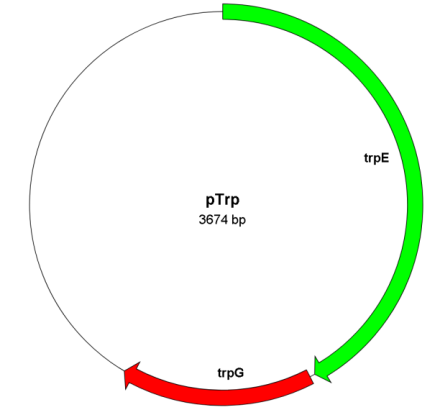

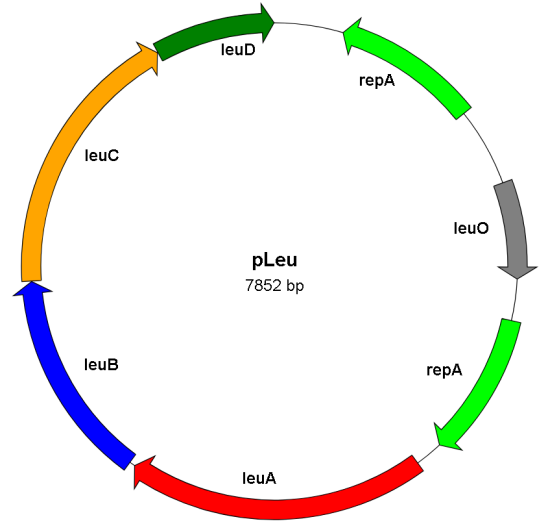


**Figure S2**. Circular view of the genome of the *Rhopalosiphum maidis* endosymbiont, *Buchnera aphidicola* (A) and its plasmids pLeu (B) and pTrp (C).


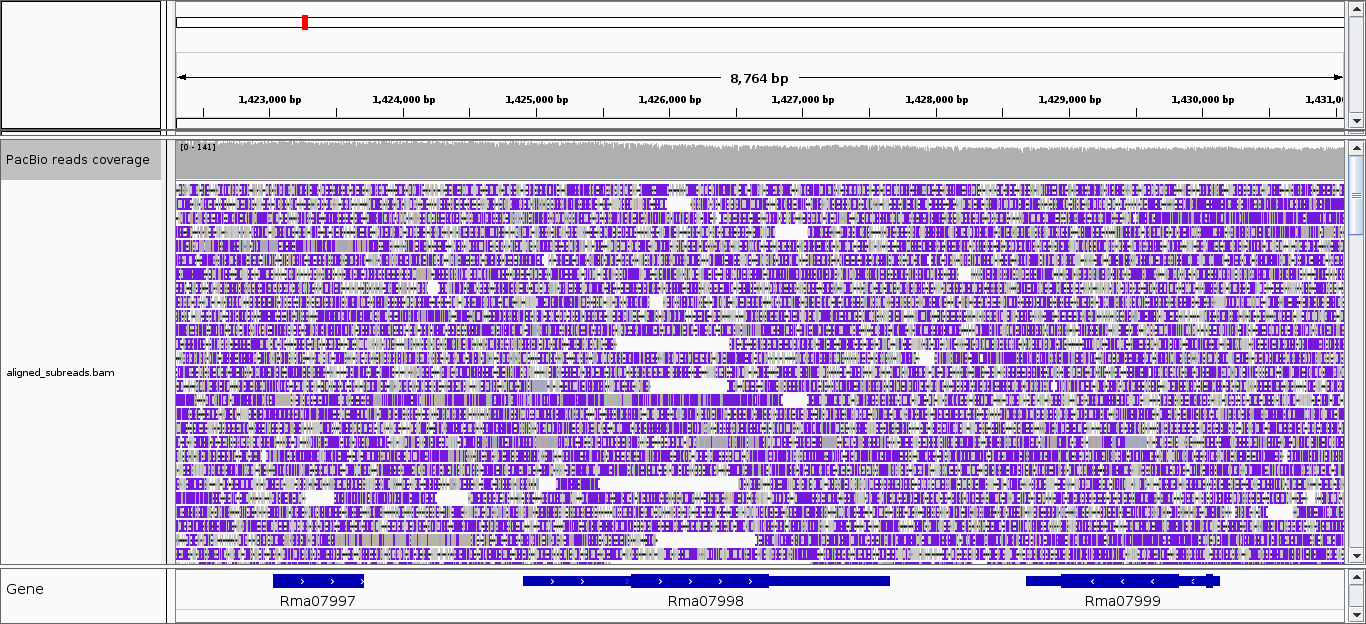


**Figure S3.** The PacBio read alignments around the Rma07998 HGT gene


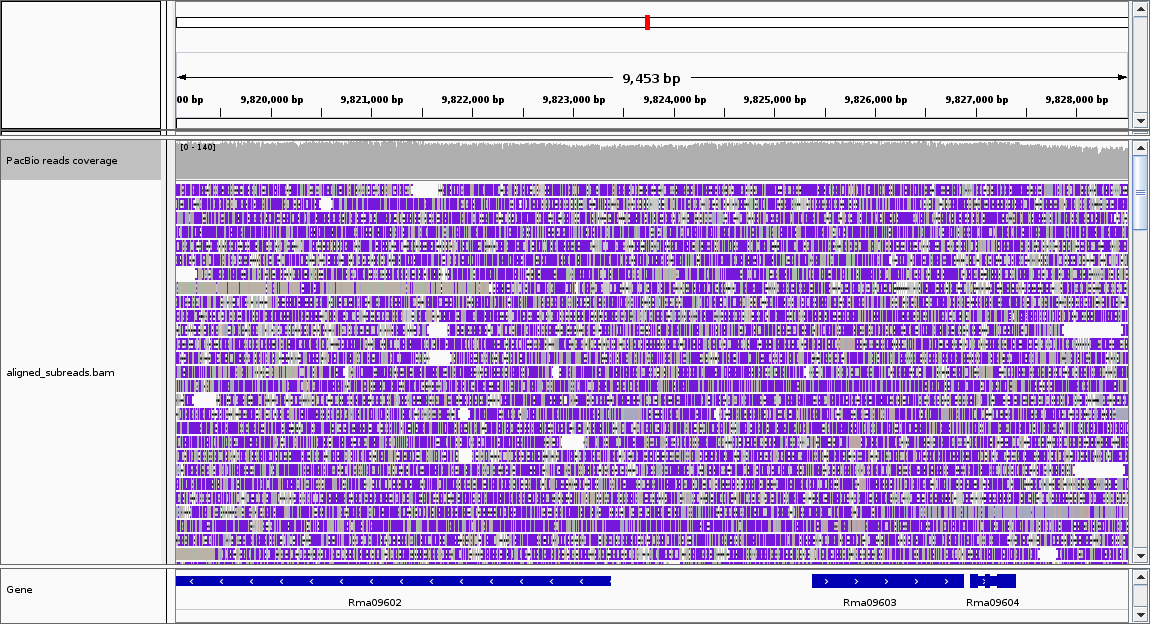


**Figure S4.** The PacBio read alignments around the Rma09603 HGT gene


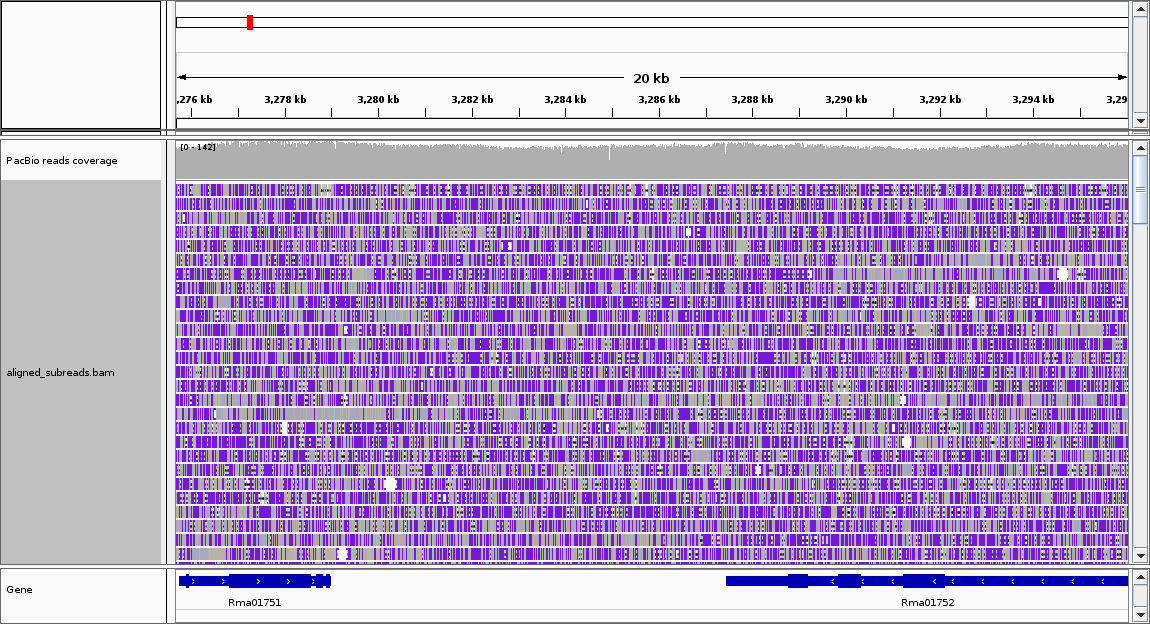


**Figure S5.** The PacBio read alignments around the Rma01752 HGT gene


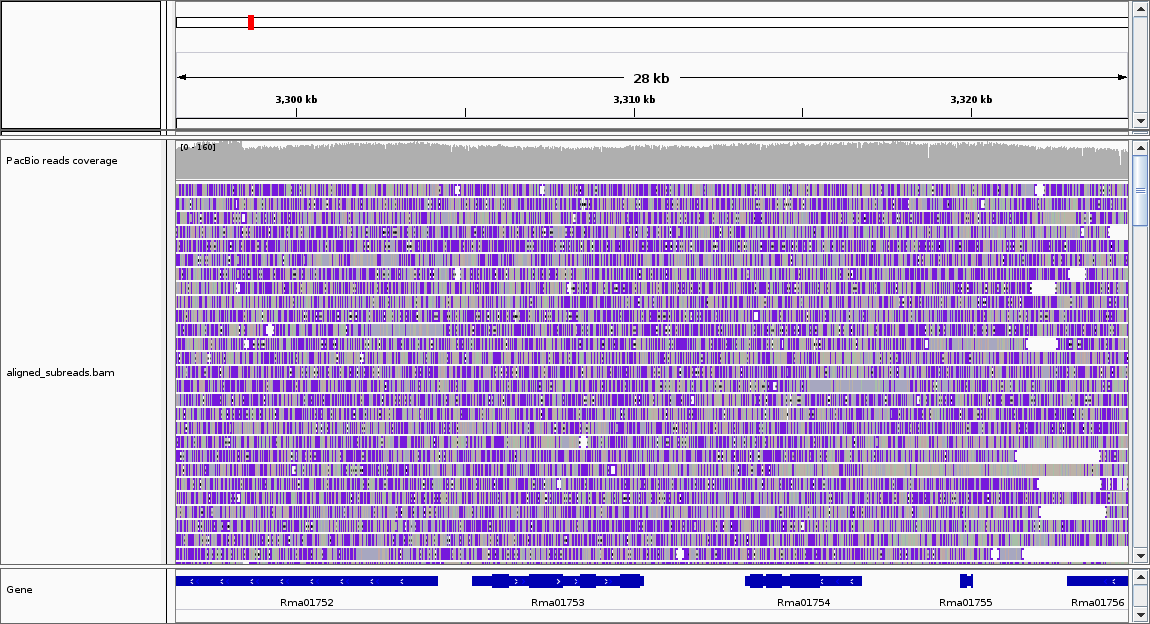


**Figure S6.** The PacBio read alignments around the Rma01753 HGT gene


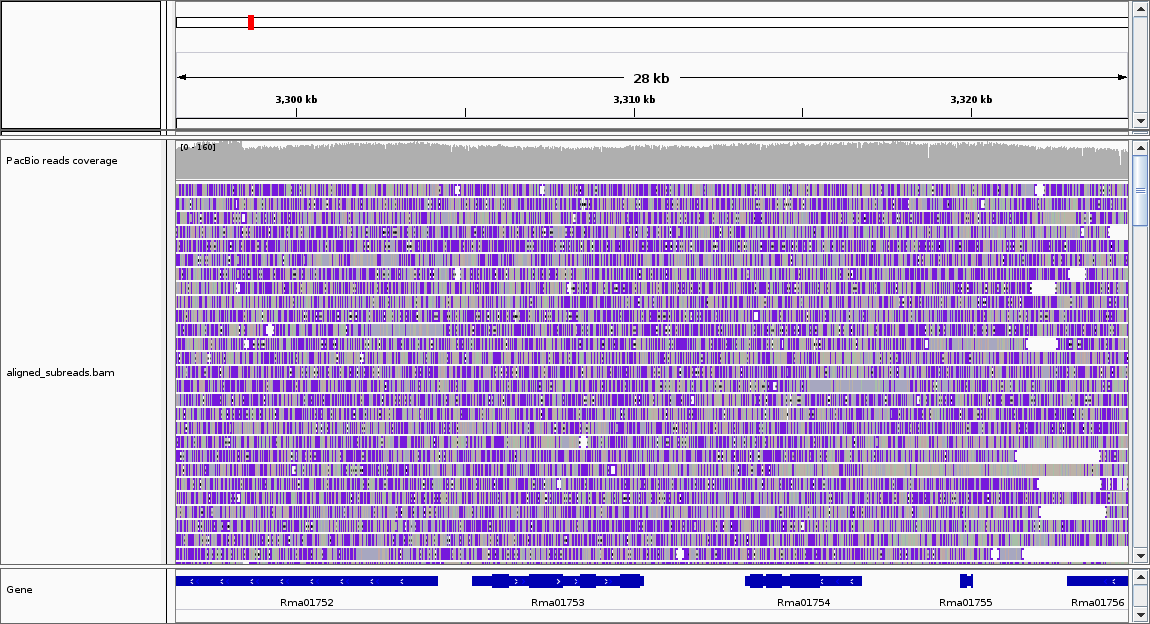


**Figure S7.** The PacBio read alignments around the Rma01754 HGT gene


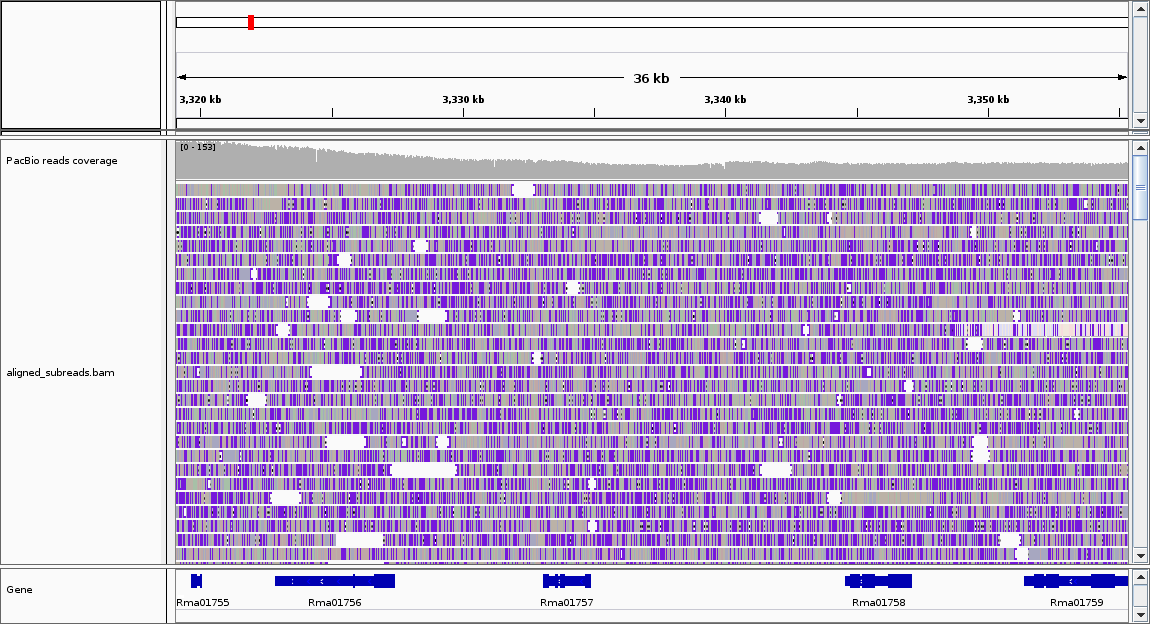


**Figure S8.** The PacBio read alignments around the Rma01756 HGT gene


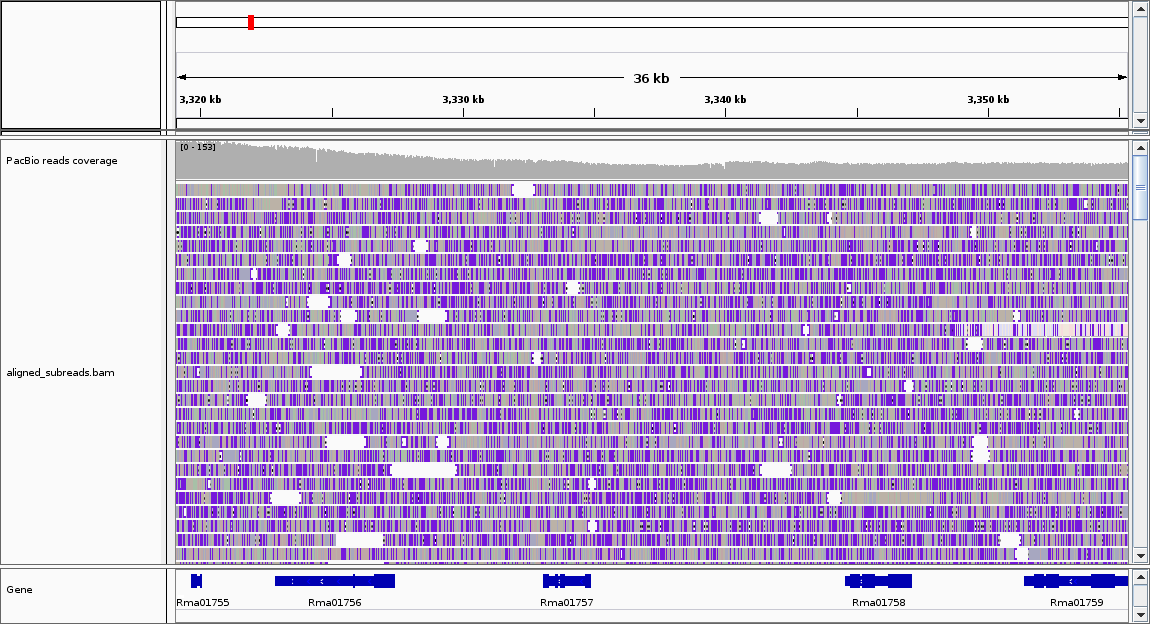


**Figure S9.** The PacBio read alignments around the Rma01758 HGT gene


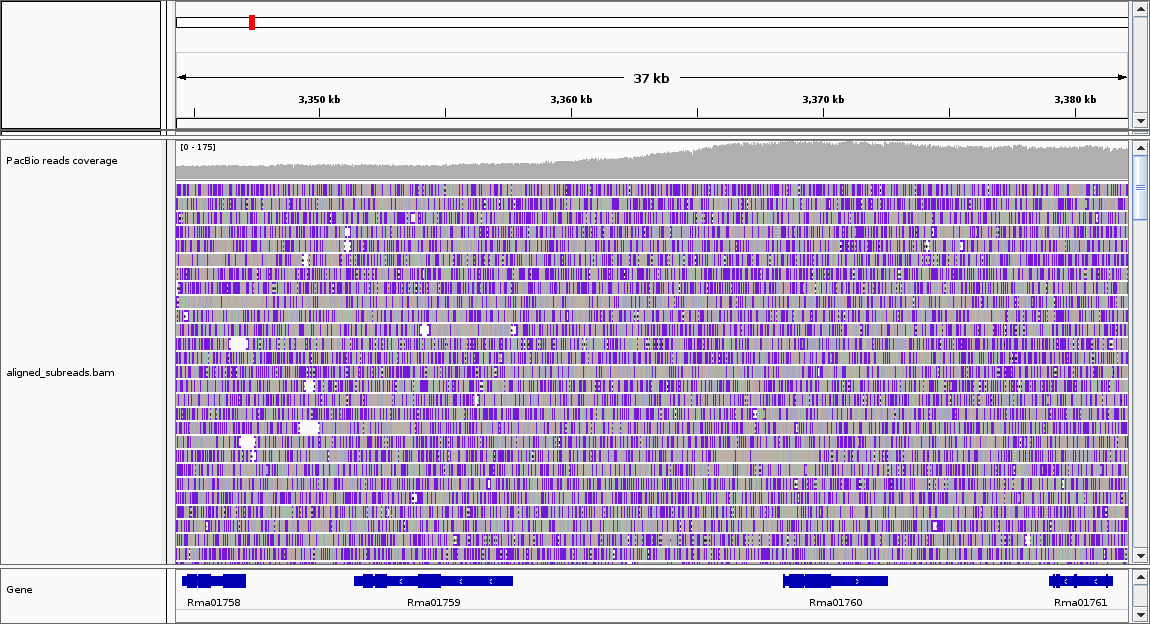


**Figure S10.** The PacBio read alignments around the Rma01759 HGT gene


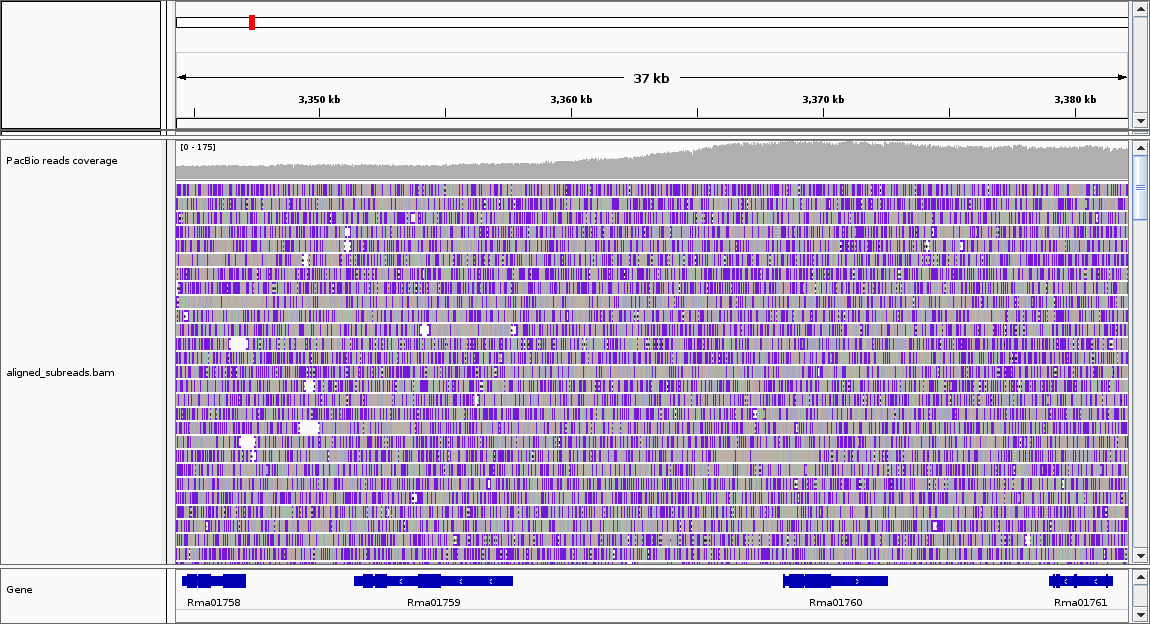


**Figure S11.** The PacBio read alignments around the Rma01760 HGT gene


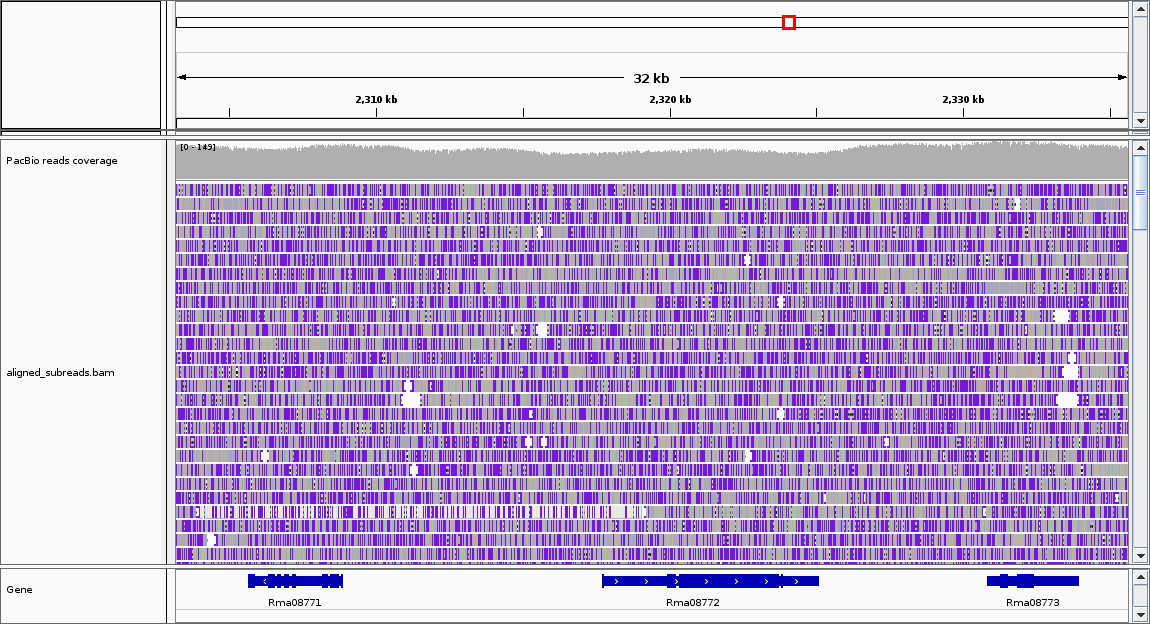


**Figure S12.** The PacBio read alignments around the Rma08772 HGT gene


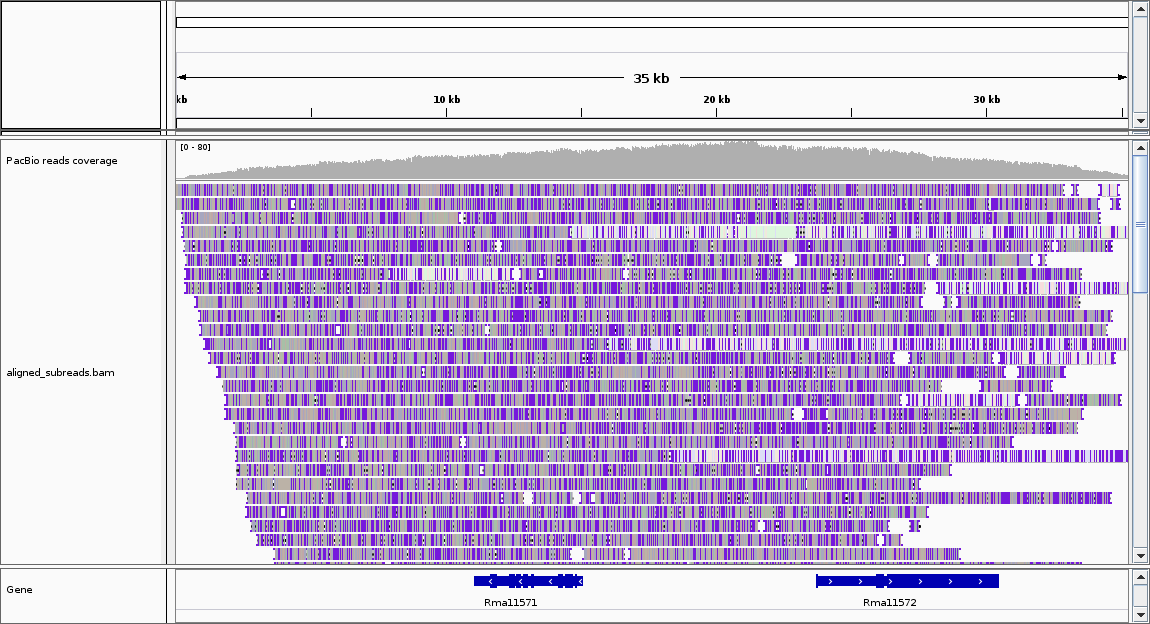


**Figure S13.** The PacBio read alignments around the Rma11572 HGT gene


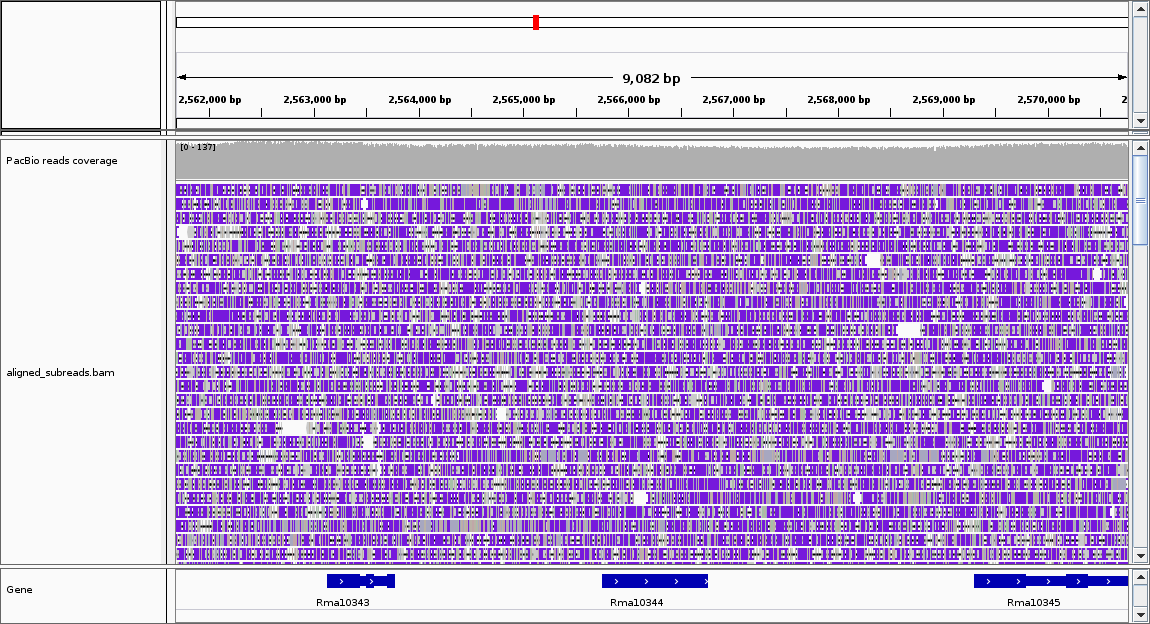


**Figure S14.** The PacBio read alignments around the Rma10344 HGT gene


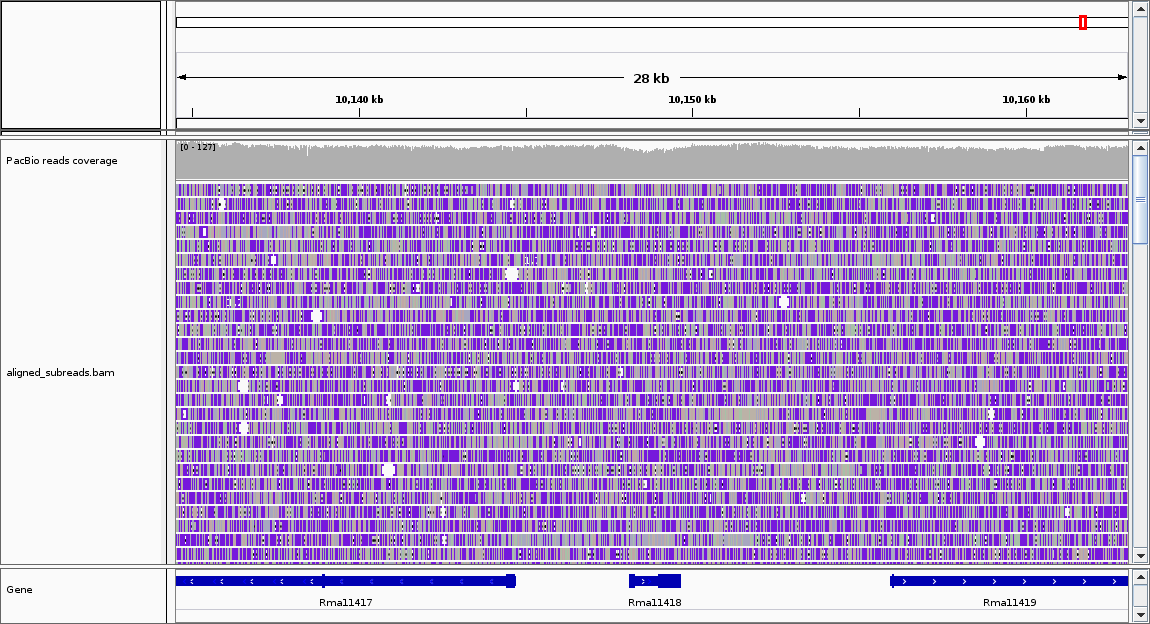


**Figure S15.** The PacBio read alignments around the Rma11418 HGT gene


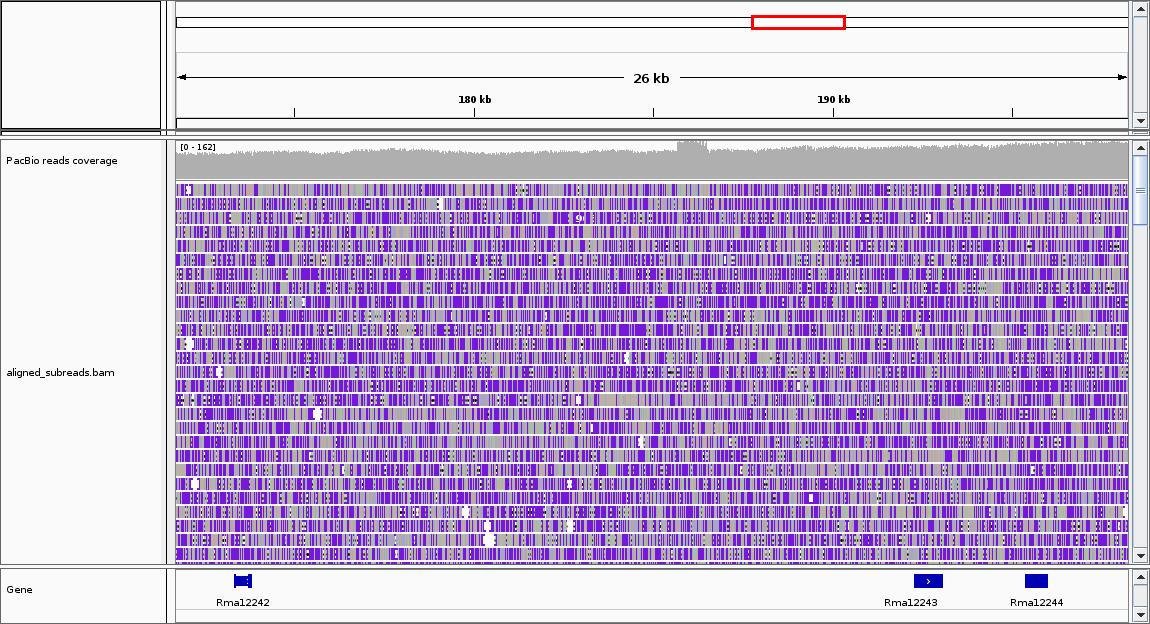


**Figure S16.** The PacBio read alignments around the Rma12243 HGT gene


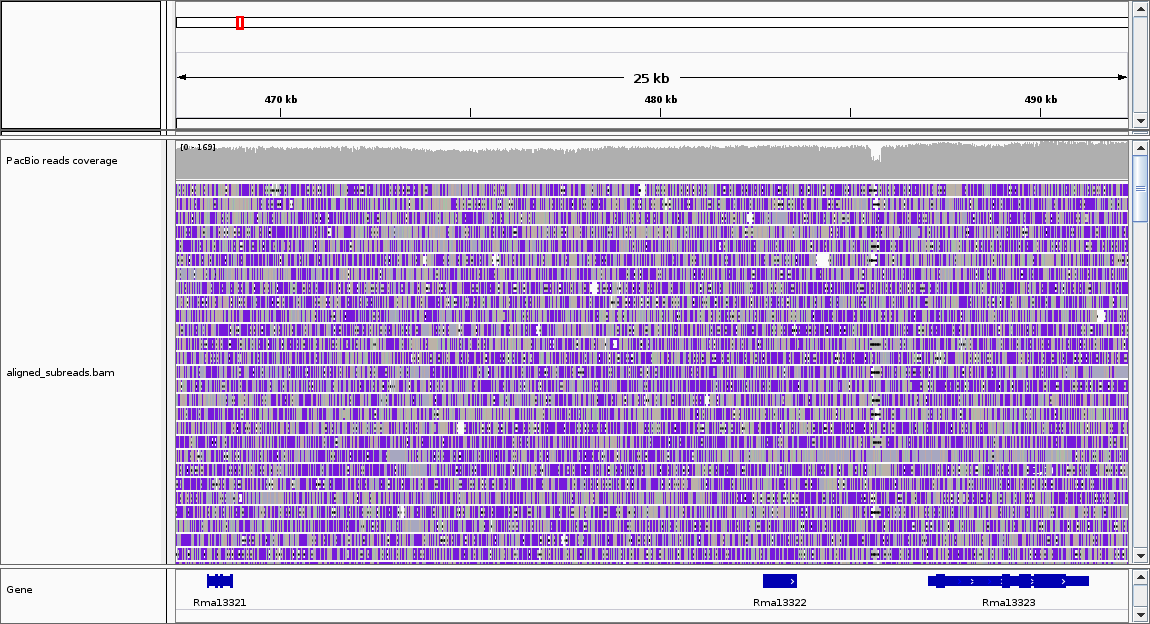


**Figure S17.** The PacBio read alignments around the Rma13322 HGT gene


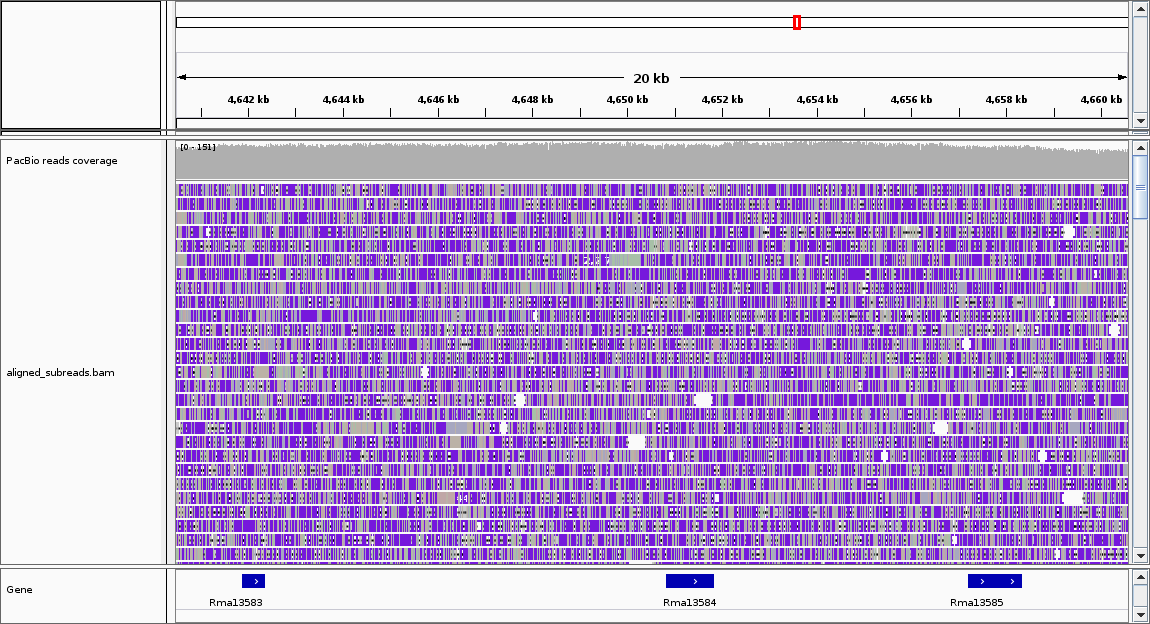


**Figure S18.** The PacBio read alignments around the Rma13584 HGT gene


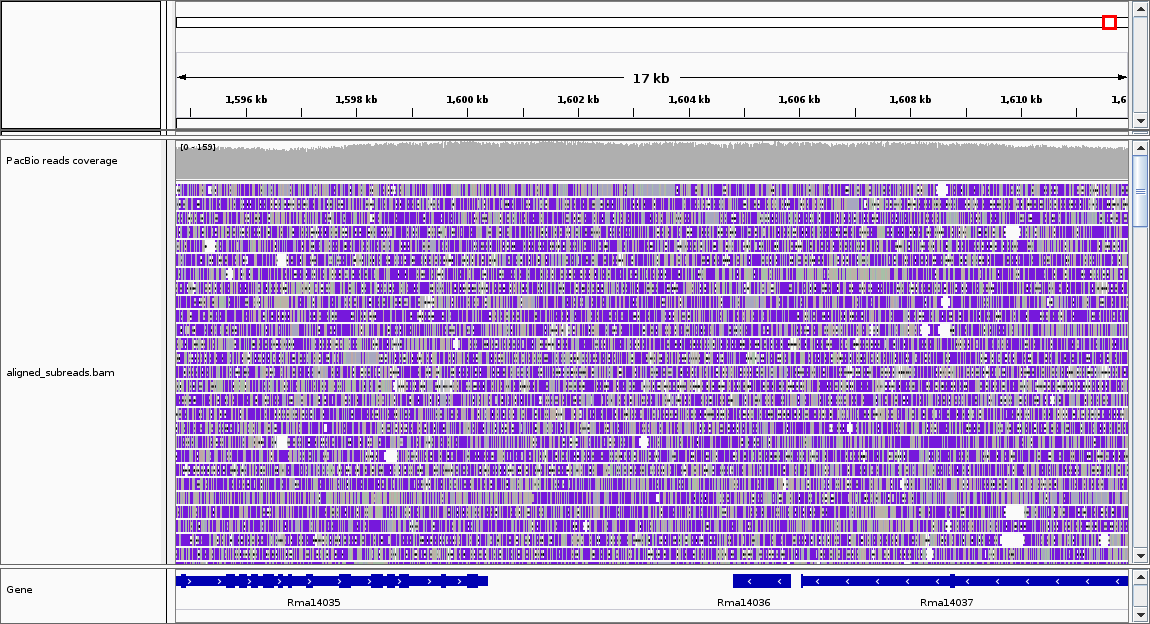


**Figure S19.** The PacBio read alignments around the Rma14036 HGT gene


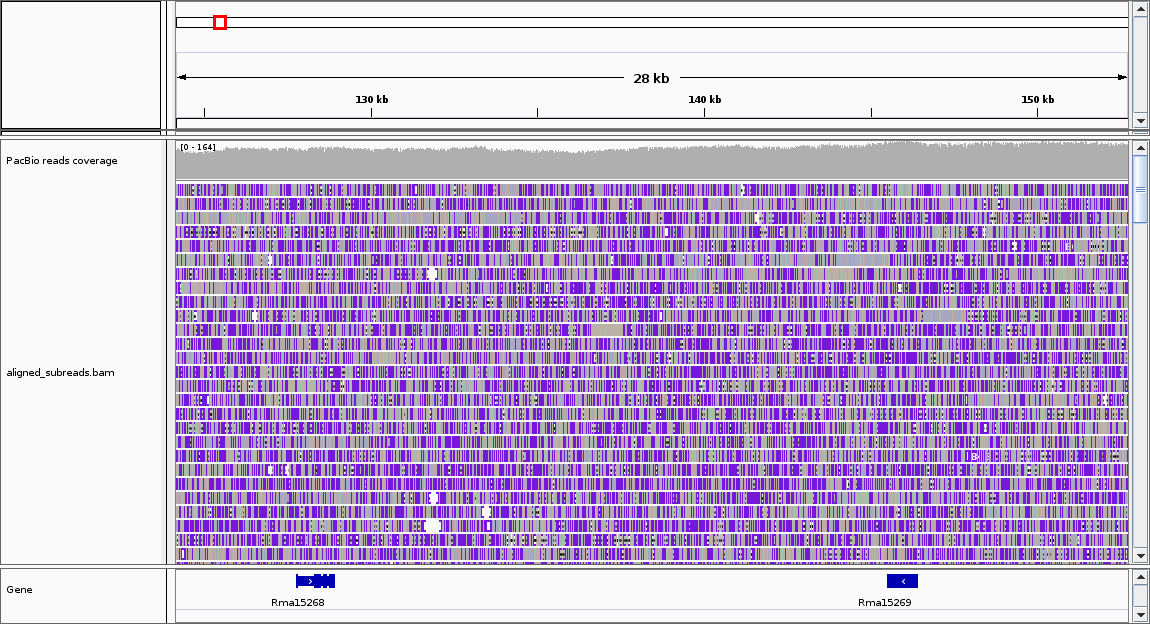


**Figure S20.** The PacBio read alignments around the Rma15269 HGT gene


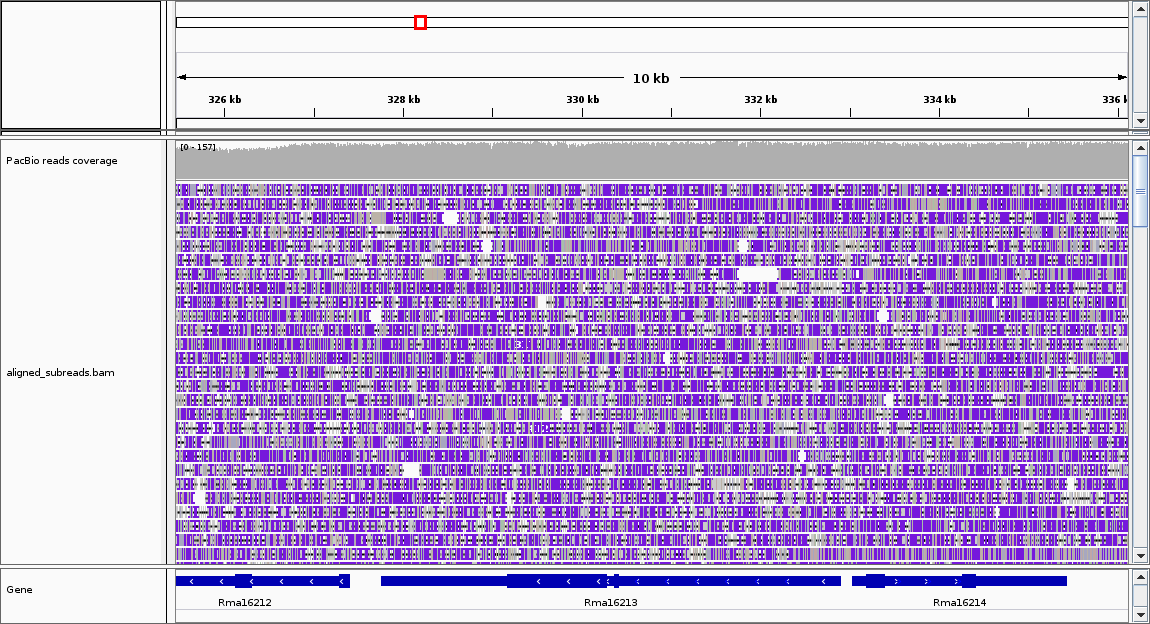


**Figure S21.** The PacBio read alignments around the Rma16213 HGT gene


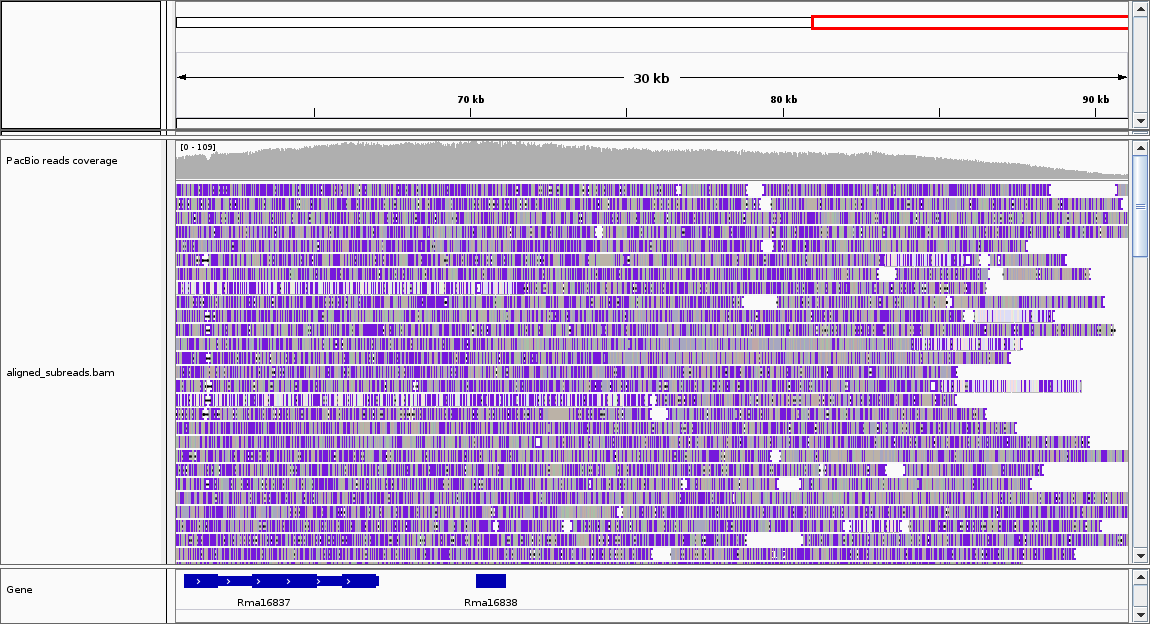


**Figure S22.** The PacBio reads alignments around the Rma16838 HGT gene


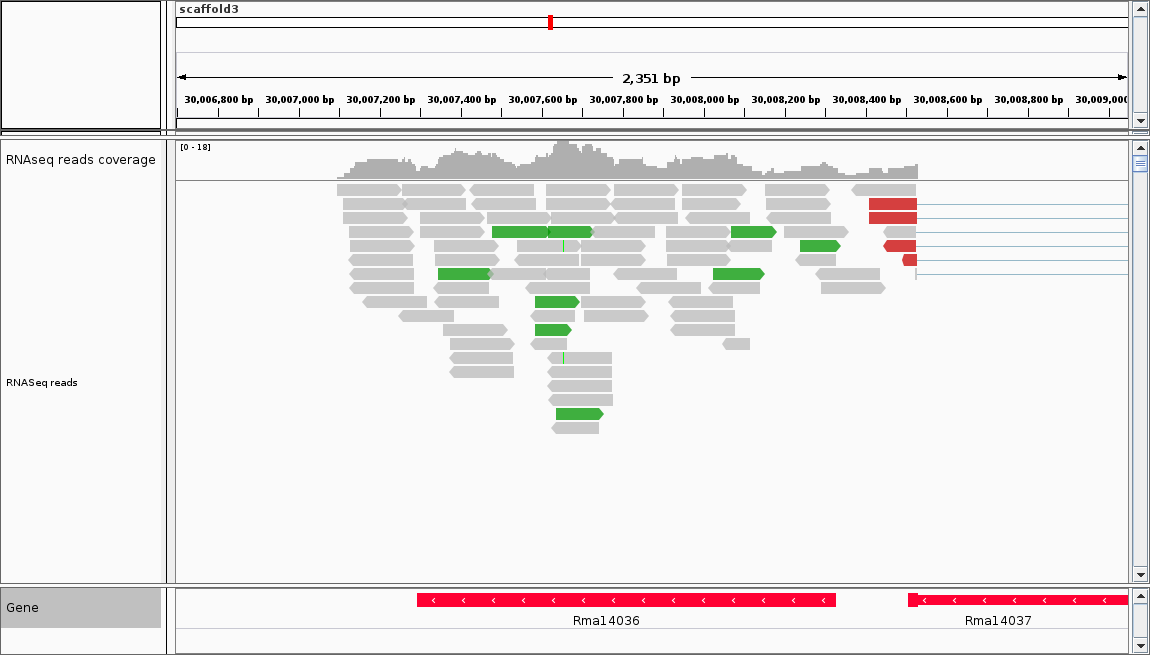


**Figure S23.** The RNA-Seq reads alignments around the Rma14036 HGT gene


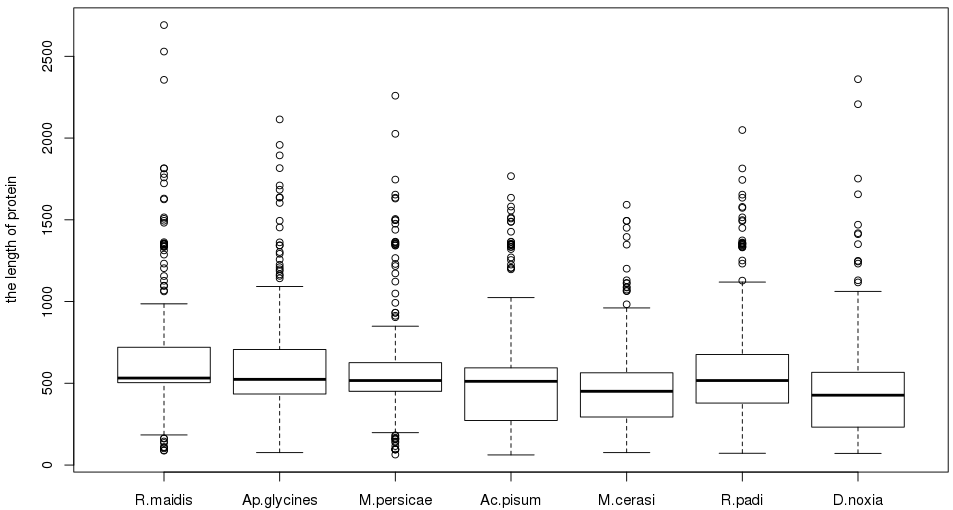


**Figure S24**. Length distribution of protein sequences of detoxification gene families in seven aphid species.
