# Supplementary material for: Genome sequence of the corn leaf aphid (Rhopalosiphum maidis Fitch)
Source: Gigascience. 2019 Apr 6;8(4):giz033. doi: 10.1093/gigascience/giz033 (PMC6451198; doi:10.1093/gigascience/giz033)
Supplement: Response_to_Reviewer_Comments_Original_Submission.pdf [file giz033_response_to_reviewer_comments_original_submission.pdf]

Author's Response To Reviewer Comments

Close

In response to suggestions from the reviewers, we have made the changes detailed below. Additions to the text of the manuscript itself are highlighted in red.

Reviewer #1:
The authors provide a nice summary of a very complete and robust genome for R. maidis. I was impressed at the level of completeness and the comparative analyses that the authors used for this Data Note manuscript. I have a couple of considerations:

It might be good in the introduction to discuss why the various types of sequencing methods were used. This will help the reader (some who may be unfamiliar) understand the various technologies and the context of their use. e.g Why the need for both pacbio and illumina? what is hi-c sequencing and Iso-Seq? Including this information would help expand the potential audience of this paper as well.
>>We have added text to the introduction to describe need for different sequencing methods (Lines 80-88).

I know this is a data note, but I think the manuscript could be improved by including a sentence or two on why no facultative symbionts were found? Is this rare for the Rhopalosiphum genus?
>>We added additional information about endosymbionts, in particular mentioning the prevalence of endosymbionts in R. padi (Lines 297-303). Since we have a sample size of one, it is not possible to say whether it is a general property of R. maidis not to have secondary symbionts.

Similarly, I think there is a lack of context for the evidence of algal horizontal gene transfer. The way the manuscript is written, it leaves the readers out to dry, especially since there has been no previous evidence from the aphids already sequenced. I would like to see a couple of sentences of interpretation here to help provide context. Is it because this study had more complete sequencing and it was just missed in other aphids? Or is it unique to Rhopalosiphum? It's further complicated since Ostreococcus is part of the picoplankton. What would be the possible reason why any genes would be transferred into R. maidis?
>>We provide additional discussion about the possible algal genes, as well as a figure of one of the phylogenetic trees (Lines 393-402; Figure 4). Tree files for the other comparisons have been added as Supplementary Data S2 to S22. Homologs of these genes are present in the other six aphid genomes. We point out that this sequence homology does not necessarily mean transfer from Ostreococcus, only that the most similar sequences in Genbank come from Ostreococcus. Different detection algorithms may account for the fact that this possible horizontal gene transfer has not been reported previously.

Gigascience guidelines include that, "Experiments supporting and demonstrating the technical quality of the dataset should be included in the manuscript." I would strongly suggest that the authors perform some quick PCR validation using genomic DNA for the presence of at least the Ostreococcus sequences, and perhaps the other odd evidence of HGT to confirm that their presence is not due to contamination.
>>In the revised manuscript we discuss three lines of evidence that the identification of these Ostreococcus-like genes is not a sequencing artefact: 1) The assembly are contiguous with aphid genes on both sides, and covered by PacBio reads, 2) the genes are present in our RNA-Seq data, indicating that they are transcribed, and 3) the genes are present the genomes of other aphid species (Line 393-402).

Other Minor Points:
Pg 3 l48 define anholocyclic for non aphidologists
pg4ln45. The DNA pellet
pg4ln55: please include the state of Mt. Sinai (if in US)
What were the aphids doing when collected for the transcriptome? Were they feeding? Starved?
pg 5 L56: shipped to the Duke Center... also include city and state
pg6ln30: include city and state for Biotechnology resource center
Pg 7 ln 24 library was not were, or change to libraries were

pg7ln30: reads were also aligned...
Pg 9 ln 9 species names should not be caps. There are a few other errors like this in the manuscript, please proofread & correct
pg11ln53. change to R. not R,
>>The requested text changes have been made.

Reviewer #2:
The manuscript describes the genome of the corn leaf aphid (Rhopalosiphum maidis Fitch) after a 326-Mb genome assembly was generated using a combination of PacBio (208-fold coverage) and Illumina sequencing (80-fold coverage). The authors obtained a total of 689 contigs with an N50 size of 9.0 Mb. The contigs were further clustered into four scaffolds using the Hi- C interaction maps, consistent with the commonly observed 2n = 8 karyotype of R. maidis. Most of the assembled contigs (473 spanning 321 Mb) were successfully orientated in the four scaffolds. The R. maidis genome assembly captured the full length of 95.8% of the core eukaryotic genes, suggesting that it is highly complete. Repetitive sequences accounted for 21.2% of the assembly, and a total of 17,647 protein-coding genes were predicted. An analysis of likely horizontally transferred genes identified two from bacteria, seven from fungi, two from protozoa, and nine from algae. This is a good contribution to current/existing genomic data on aphids. However, more information could have been supplied on the genes, gene function and differences between the corn leaf aphid and other aphid genomes. The paper is very descriptive, instead of being insightful on what is unique to this aphis species. Particularly the manuscript is lacking in information regarding:

* Genomic constituency, i.e. heterozygosity of genome, GC content, % coding vs. % non-coding
>> The heterozygosity rate of the genome calculated by bbmap (https://jgi.doe.gov/data-and-tools/bbtools/bb-tools-user-guide/bbmap-guide/) is less than 0.00001, which is consistent with the k-mer distribution. The GC content is 27.7%. Coding region: 6.7% of total genome. Intron region: 42.0% of total genome. Intergenic: 51.2% of total genome. This information has been added to the manuscript text (Lines 269-274).

* Genome assembled into 4 chromosomes. What are the gene contents of each? Which is the X-chr? etc.
>>The gene content information has been added as Table 3 in the revised manuscript. We were not able to unambiguously determine the X-chromosome based markers known to be associated with aphid X chromosomes. Four M. persicae X-chromosome markers (Wilson et al, 2014, DOI:10.1371/journal.pone.0116348) were located in three separate contigs on chromosome 3 of R. maidis. However, markers associated with the X-chromosome in pea aphids (Jaquiéry et al, 2018, doi: 10.1093/gbe/evy015; and Jaquiéry et al, 2013, https://doi.org/10.1371/journal.pgen.1003690) were located on multiple chromosomes in R. maidis. This information has been added in lines 277-282.

* What are the ±24 % of unique genes? What functions do they carry (GO)?
>>4248 genes have no significant homology with the pea aphid genome. Of these, 4026 (94.7%) were annotated as "Unknown protein", and only 41 genes have GO annotations (Lines 345-347). The GO annotation of these 41 genes have been uploaded to the GigaDB FTP.

* Contigs that were excluded from analysis due to BLAST matches to bacteria/viruses → what were they? Isn't additional endosymbionts captured there? Although screening for endosymbionts was performed, whole contigs were used which may indicate contain new endosymbionts? At the least, what were the lengths and counts of these excluded contigs?
>> We BLASTed the assembled contigs against the NCBI nt database, and identified contamination based on taxonomy. This analysis identified Archaea (Methanobacterium formicicum), Bacteria (Cronobacter dublinensis, Escherichia coli, Methylobacterium zatmanii, Planktothrix agardhii, Salmonella enterica Weissella cibaria), Eukaryota (Plasmodium falciparum, Brugia pahangi, Emplectanthus cordatus, Gossypium arboretum, Zea mays, Theobroma cacao, Frankliniella intonsa, Oryzias latipes), and plasmids. If >90% of an individual contig had non-aphid DNA, it was considered to be contamination. We did not identify genomes of previously known endosymbiont bacteria, or any sequences that are likely to represent the full genomes of other bacterial species. This information is presented in lines 243-253) in the revised manuscript. The contigs are uploaded to the GigaDB FTP.

Reviewer #3:
The authors reported a high-quality genome assembly of the corn leaf aphid, Rhopalosiphum maidis Fitch. This is the first chromosome-level reference genome for aphids. I congratulate the authors on the excellent genome assembly generated by the combination of long-read sequencing and Hi-C technology.

This genomic information is undoubtedly a valuable resource for a wide range of research fields, including aphid biology, pest control research, and insect genomics. However, as outlined below, there are several issues that need to be addressed before the paper can be suitable for publication.

Major issues

Information on sampling and genome sequencing are not provided enough (pp.3-5). Since this study is the most successful genome sequencing in aphids thus far, detailed procedures would be useful for readers. I list specific points as follows:

* p.4: Around 50 ug of high molecular weight DNA was prepared … : High molecular weight (HMW) genome preparation is key to a successful genome project. How long was it? How did the authors evaluate the HMW genome? (e.g., pulse field electrophoresis)
>> The size of the DNA was not confirmed, other than by the length of the PacBio sequencing reads. More details regarding the DNA and RNA extraction have been added (lines 110-148, 152-180, 191-192, 210-211).

* p.4: … using SMRT Cell template preparation kits … : Detailed procedures should be described. For example, what is the size selection method (e.g., BluePippin)? How much is the insert size of the resultant library?
>>This information has been added (lines 136-137).

* p.4: … run on the PacBio Sequel platform …: Detailed sequencing condition should be described. Which version of PacBio chemistry did they use? (e.g., M1 v2). How long was the movie time?
>>This information has been added (lines 144-146).

* p.5.: … using the Illumina TruSeq DNA sample preparation kit following the manufacturer's instructions: Detailed prep information should be provided. Was it a PCR-free library? How long was the insert size of the library?
>>This information has been added (lines 152-180).

* p.5: Transcriptome sequencing: Sample information should be provided. Which tissue? (Whole body?) Adults or nymphs?
>>This information has been added. We used mixed-instar whole aphids from barley (lines 191-192).

* p5: Iso-seq: … for PacBio large-insert (15-20kb) library construction ... : It sounds weird. Iso-seq library should not be long-insert one. It is typically below several kb inserts. A standard PacBio Iso-seq preparation is composed of SMARTer PCR cDNA Synthesis and SMRTbell library construction. Did the author use a special technique for Iso-Seq? If it is the case, the detailed procedures should be described in the manuscript.
>>>>This was a mistake in the writing of the manuscript. The actual library insert size was 500 bp to 4500 bp. We have corrected the methods section accordingly (lines 210-211).

* They reported the genomes of R. maidis and Buchnera symbiont in this manuscript. I am wondering if the authors found a mitochondrial genome in the assembly as well?
>>The 18,060 bp mitochondrial genome sequence was assembled and this information has been added to the manuscript. The full sequence is in GenBank MK368778. Text was added to the manuscript at lines 273-274.

* Although the authors did not emphasize in the current manuscript, one of the great things of this assembly is that it contains only a small fraction of ambiguous sequences (Ns) in the assembly thank to the long-read technology. I downloaded the sequence data and calculated as 469 kbp (0.14%) as Ns, which is much less than precedent aphid genome assemblies reported. I think the authors can mention this point in the manuscript.
>> We used PacBio long reads and Illumina reads to assemble the genome into contigs without Ns. Then we ordered and orientated contigs into scaffolds using Hi-C data. As HiC data do not provide the exact number of bp between the oriented contigs, Phase Genomics LACHESIS arbitrarily adds 100 Ns between contigs (lines 255-256. All of the Ns in the assembly come from this.

* Given the chromosome-level assembly, it is natural to ask which is the sex chromosome (X) among the four long scaffolds? (Aphid sex determination is XO system). Several labs identified X-linked genes in other aphids such as Acyrthosiphon pisum, which might be used to predict X chromosome of R.

maidis. It would be great if the authors will include this analysis in the revised manuscript if possible. However, the identification of X chromosome might not be as straightforward as I expect; it's ok as is. I do not think lack of the X chromosome analysis should prevent publication and some researchers will address this issue in the future using the genome sequence described here.
>> We were not able to unambiguously determine the X-chromosome based markers known to be associated with aphid X chromosomes. Four M. persicae X-chromosome markers (Wilson et al, 2014, DOI:10.1371/journal.pone.0116348) where located in three separate contigs on chromosome 3 of R. maidis. However, markers associated with the X-chromosome in pea aphids (Jaquiéry et al, 2018, doi: 10.1093/gbe/evy015; and Jaquiéry et al, 2013, https://doi.org/10.1371/journal.pgen.1003690) were located on multiple chromosomes in R. maidis. This information has been added in lines 277-282.

* p7: Contaminated contig detection: They just searched against NCBI nt database using BLASTN. It sounds a naive strategy. I recommend read coverage should be considered as well. Alternatively, sophisticated software such as BlobTools can be used for contamination detection.
>>We BLASTed the contigs against the NCBI nt database with the parameter -evalue 1e-5 -outfmt "6 qseqid sseqid pident length mismatch gapopen qstart qend sstart send evalue bitscore qlen slen sstrand staxids sscinames sskingdoms stitle", which have already used when considering taxonomy in our detection of contaminating contigs (similar to what would be done by BlobTools). Based on this, we excluded 57 contigs, totaling 930,609 bp from the assembly (Lines 242-253).
We don't think read coverage would give much meaningful information here for filtering as the contaminated sequences are from different sources, so they are expected to have a variety of different read coverages. In addition, read coverages of the actual aphid sequences vary due to repetitive regions and regions that are recalcitrant to amplification and/or cloning. Furthermore, only ~0.8% of the assembled contigs were not assigned to chromosomes based on the Hi-C data, suggesting that remaining contaminant sequences in our assembly would be minor, if there are any.

* p.9: Discussion on the gene numbers: The authors imply that the gene number difference among aphid species is associated with the genome size, but there are other possibilities. One of the likely causes is just the difference of gene prediction method between the genome projects. Actually, Thorpe and his colleagues reanalyzed 5 aphid genomes and annotated genes using the same pipeline and concluded that aphid genomes consistently encode similar gene numbers (P. Thorpe et al. 2018 GBE). I do not intend to request the authors to reanalyze R. maidis gene models from scratch, which is a big deal, but I request careful editing of the text in this part of the manuscript.
>>We have added a discussion of the gene numbers that are obtained and explain that this is likely due to different analysis methods (Lines 330-337).

* p.10: Horizontal gene transfer detection: h score alone is not convincing. They mentioned " … manually checked for potential genome assembly errors …", but they did not mention the procedure and the criteria in detail. I still concern the possibility of the contamination.
>>We provide additional explanation of this analysis in the revised manuscript. The corresponding genome sequences of these candidates as well as flanking aphid genes at both ends were manually checked using IGV (Supplementary Figures S3-S22). If there is any region with substantially reduced coverage between HGTs and their flanking aphid genes, then this HGTs could be misassembled. Only HGTs in genomic regions with continuous read coverage were considered to be confirmed (lines 369-374, 390-399)

Regarding Data Set hosted in GigaScience FTP, I have some suggestions to add more data discussed in the manuscript. I list the specific suggestions as follows:

* OrthoMCL ortholog table should be provided.
* It would be great if CDS fasta could be provided.
* It would be great if annotation files (InterPro search results and GO terms) were provided. They would be extremely useful for readers.
>>These files have been uploaded to the GigaDB FTP and will be made available to reviewers by the journal.

Minor issues

* p3 anholocyclic: general audience (non-aphid researchers) may not be familiar with this word. It needs some explanation.
>>This information has been added (lines 72-73)

* Table 1: assembly size of R. maidis is described as 326.0 Mb — but I calculated the total length of the assembly from the downloaded data and found it 326,445,255 bp.
>> Version 2 of the scaffolds is 326,023,155 bp. This difference is largely due to the number of Ns that were added between contigs during the scaffolding. Whereas 1000 Ns were added in the first assembly, only 100 Ns were added during our re-analysis. We have uploaded the corrected sequence.

* p7: … aligning the initial assembly to the Buchnera reference genome...: The authors should specify which Buchnera is. NC_002528.1 is Buchnera aphidicola of the pea aphid (Acyrthosiphon pisim). Also, they should cite the corresponding paper (Shigenobu et al. 2000 Nature).
>>This information has been added (line 287).

* p8: Secondary symbiont detection: They simply mentioned that they used BLAST. The details such as search parameters should be described.
>> The parameters used were: -dust yes -max_target_seqs 10 -evalue 1e-5 -outfmt "6 qseqid sseqid pident length mismatch gapopen qstart qend sstart send evalue bitscore qlen slen sstrand staxids sscinames sskingdoms stitle". We classify the hits by taxonomy. This information has been added to the manuscript (lines 243-244).

* p8: In Gene prediction section: … completed proteomes of A.pisum, A. glycine … : what are the sources of the proteome sequences? AphidBase or NCBI RefSeq? Sometimes the gene set of AphidBase and NCBI is different each other.
>> We downloaded the proteome sequences from UniProt. This information has been added in line 327.

Some typos:

* p6: Hi-C Libraries => Hi-C libraries
* p9: Ac. Pisum => Ac. pisum
* p11: Ac. Pisum => Ac. pisum
* p16 ref 32 extractino => extraction
>>Corrections have been made.

Reviewer #4 (annotated manuscript)

PacBio sequencing = 70Gb (208x coverage)
Illumina sequencing = 75Gb (80 x coverage)
How???
>> The raw Illumina reads were 75 Gb. However, the sequences were relatively low quality. After remove adaptors and low quality sequences, only 28 Gb data remained. This is 78X coverage of the genome. The manuscript has been corrected accordingly (lines 28-29).

Please add citation for: "The origin of R. maidis is likely in Asia, and it has been subsequently introduced in most grain-growing areas of the world."
>>Citation has been added (line 72).

"There is also evidence…"
>>"There also is evidence of host specificity among the karyotypes." has been changed to "There is also evidence of host specificity among the karyotypes."

Although seemingly used to prevent oxidation of phenolic compounds, Sodium bisulfite can
>>We agree that sodium bisulfite can be used for other things. However, due to the abrupt termination of this comment, we do not know what information the reviewer would like us to add to the manuscript.

Reported 80x coverage for 75Gb Illumina and 208x coverage for 70Gb Pac-Bio???
>> The raw Illumina reads were 75 Gb. However, the sequences were relatively low quality. After removing adaptors and low quality sequences, only 28 Gb data remained. This is 78X coverage of the genome. The manuscript has been corrected accordingly.

We phylogenetically validated all HGT candidates. How exactly?
>>We have provided additional information regarding the methods that were used (lines 28-29).

Close
