# Supplementary material for: Genome sequence of the corn leaf aphid (Rhopalosiphum maidis Fitch)
Source: Gigascience. 2019 Apr 6;8(4):giz033. doi: 10.1093/gigascience/giz033 (PMC6451198; doi:10.1093/gigascience/giz033)
Supplement: Response_to_Reviewer_Comments_Revision_1.pdf [file giz033_response_to_reviewer_comments_revision_1.pdf]

Author's Response To Reviewer Comments

Close

>>In response to reviewer comments, we have made the described change to our manuscript. The change is marked in red in the resubmitted manuscript.

Reviewer #3:
The authors have done a good job in addressing the previous comments and improved the manuscript significantly. In particular, they provided sufficient information on the materials and methods in the revised manuscript. I am almost satisfied with the revision. I have only a single issue to ask the authors to confirm as below:

* L157 : DNA samples were transferred to a Covaris G-tube … : G-tube is usually used to shear DNA into fragments ranging from 6kb to 20kb (see manufacture's website); it is typically used in long-read NGS library prep. The target fragmentation size of the PCR-free Illumina library was 550 bp as described in the manuscript (L161). I concern the discrepancy.

>>This was a mistake. We have changed "Covaris G-tube" to "Covaris microTUBE" in this sentence.

Close
