# Supplementary material for: Genome sequence of the corn leaf aphid (Rhopalosiphum maidis Fitch)
Source: Gigascience. 2019 Apr 6;8(4):giz033. doi: 10.1093/gigascience/giz033 (PMC6451198; doi:10.1093/gigascience/giz033)
Supplement: Response_to_Reviewer_Comments_Revision_2.pdf [file giz033_response_to_reviewer_comments_revision_2.pdf]

Author's Response To Reviewer Comments

Close

We have made the requested changes to the manuscript, adding ORCIDs and the taxon ID.

Close
