# Supplementary material for: Genome sequence of the corn leaf aphid (Rhopalosiphum maidis Fitch)
Source: Gigascience. 2019 Apr 6;8(4):giz033. doi: 10.1093/gigascience/giz033 (PMC6451198; doi:10.1093/gigascience/giz033)
Supplement: Reviewer_1_Report_Original_Submission -- Andrew Michel — 11/15/2018 Reviewed [file giz033_reviewer_1_report_original_submission.pdf]

Reviewer Report

Title: Genome sequence of the corn leaf aphid (Rhopalosiphum maidis Fitch)

Version: Original Submission **Date:** 11/15/2018

Reviewer name: Andrew Michel

Reviewer Comments to Author:

The authors provide a nice summary of a very complete and robust genome for R. maidis. I was impressed at the level of completeness and the comparative analyses that the authors used for this Data Note manuscript. I have a couple of considerations:
It might be good in the introduction to discuss why the various types of sequencing methods were used. This will help the reader (some who may be unfamiliar) understand the various technologies and the context of their use. e.g Why the need for both pacbio and illumina? what is hi-c sequencing and Iso-Seq? Including this information would help expand the potential audience of this paper as well.
I know this is a data note, but I think the manuscript could be improved by including a sentence or two on why no facultative symbionts were found? Is this rare for the Rhopalosiphum genus?
Similarly, I think there is a lack of context for the evidence of algal horizontal gene transfer. The way the manuscript is written, it leaves the readers out to dry, especially since there has been no previous evidence from the aphids already sequenced. I would like to see a couple of sentences of interpretation here to help provide context. Is it because this study had more complete sequencing and it was just missed in other aphids? Or is it unique to Rhopalosiphum? It's further complicated since Ostreococcus is part of the picoplankton. What would be the possible reason why any genes would be transferred into R. maidis? Gigascience guidelines include that, "Experiments supporting and demonstrating the technical quality of the dataset should be included in the manuscript." I would strongly suggest that the authors perform some quick PCR validation using genomic DNA for the presence of at least the Ostreococcus sequences, and perhaps the other odd evidence of HGT to confirm that their presence is not due to contamination.
Other Minor Points:
Pg 3 l48 define anholocyclic for non aphidologists
pg4ln45. The DNA pellet
pg4ln55: please include the state of Mt. Sinai (if in US)
What were the aphids doing when collected for the transcriptome? Were they feeding? Starved?
pg 5 L56: shipped to the Duke Center... also include city and state
pg6ln30: include city and state for Biotechnology resource center
Pg 7 ln 24 library was not were, or change to libraries were
pg7ln30: reads were also aligned...
Pg 9 ln 9 species names should not be caps. There are a few other errors like this in the manuscript, please proofread & correct
pg11ln53. change to R. not R,

Level of Interest

Please indicate how interesting you found the manuscript: Choose an item.

Quality of Written English

Please indicate the quality of language in the manuscript: Choose an item.

Declaration of Competing Interests

Please complete a declaration of competing interests, considering the following questions:

- Have you in the past five years received reimbursements, fees, funding, or salary from an organisation that may in any way gain or lose financially from the publication of this manuscript, either now or in the future?
- Do you hold any stocks or shares in an organisation that may in any way gain or lose financially from the publication of this manuscript, either now or in the future?
- Do you hold or are you currently applying for any patents relating to the content of the manuscript?
- Have you received reimbursements, fees, funding, or salary from an organization that holds or has applied for patents relating to the content of the manuscript?
- Do you have any other financial competing interests?
- Do you have any non-financial competing interests in relation to this paper?

If you can answer no to all of the above, write 'I declare that I have no competing interests' below. If your reply is yes to any, please give details below.

I declare that I have no competing interests

I agree to the open peer review policy of the journal. I understand that my name will be included on my report to the authors and, if the manuscript is accepted for publication, my named report including any attachments I upload will be posted on the website along with the authors' responses. I agree for my report to be made available under an Open Access Creative Commons CC-BY license (http://creativecommons.org/licenses/by/4.0/). I understand that any comments which I do not wish to be included in my named report can be included as confidential comments to the editors, which will not be published.

I agree to the open peer review policy of the journal

To further support our reviewers, we have joined with Publons, where you can gain additional credit to further highlight your hard work (see: https://publons.com/journal/530/gigascience). On publication of this paper, your review will be automatically added to Publons, you can then choose whether or not to claim your Publons credit. I understand this statement.

Yes Choose an item.
