# Supplementary material for: Genome sequence of the corn leaf aphid (Rhopalosiphum maidis Fitch)
Source: Gigascience. 2019 Apr 6;8(4):giz033. doi: 10.1093/gigascience/giz033 (PMC6451198; doi:10.1093/gigascience/giz033)
Supplement: Reviewer_2_Report_Original_Submission -- Anna-Maria Botha — 11/21/2018 Reviewed [file giz033_reviewer_2_report_original_submission.pdf]

Reviewer Report

Title: Genome sequence of the corn leaf aphid (Rhopalosiphum maidis Fitch)

Version: Original Submission **Date:** 11/21/2018

Reviewer name: Anna-Maria Botha

Reviewer Comments to Author:

The manuscript describes the genome of the corn leaf aphid (Rhopalosiphum maidis Fitch) after a 326-Mb genome assembly was generated using a combination of PacBio (208-fold coverage) and Illumina sequencing (80-fold coverage). The authors obtained a total of 689 contigs with an N50 size of 9.0 Mb. The contigs were further clustered into four scaffolds using the Hi- C interaction maps, consistent with the commonly observed 2n = 8 karyotype of R. maidis. Most of the assembled contigs (473 spanning 321 Mb) were successfully orientated in the four scaffolds. The R. maidis genome assembly captured the full length of 95.8% of the core eukaryotic genes, suggesting that it is highly complete. Repetitive sequences accounted for 21.2% of the assembly, and a total of 17,647 protein-coding genes were predicted. An analysis of likely horizontally transferred genes identified two from bacteria, seven from fungi, two from protozoa, and nine from algae. This is a good contribution to current/existing genomic data on aphids. However, more information could have been supplied on the genes, gene function and differences between the corn leaf aphid and other aphid genomes. The paper is very descriptive, instead of being insightful on what is unique to this aphis species. Particularly the manuscript is lacking in information regarding:

* Genomic constituency, i.e. heterozygosity of genome, GC content, % coding vs. % non-coding
* Genome assembled into 4 chromosomes. What are the gene contents of each? Which is the X-chr? etc.
* What are the Â±24 % of unique genes? What functions do they carry (GO)?
* Contigs that were excluded from analysis due to BLAST matches to bacteria/viruses â†’ what were they? Isn't additional endosymbionts captured there? Although screening for endosymbionts was performed, whole contigs were used which may indicate contain new endosymbionts? At the least, what were the lengths and counts of these excluded contigs?

Level of Interest

Please indicate how interesting you found the manuscript: Choose an item.

Quality of Written English

Please indicate the quality of language in the manuscript: Choose an item.

Declaration of Competing Interests

Please complete a declaration of competing interests, considering the following questions:

- Have you in the past five years received reimbursements, fees, funding, or salary from an organisation that may in any way gain or lose financially from the publication of this manuscript, either now or in the future?
- Do you hold any stocks or shares in an organisation that may in any way gain or lose financially from the publication of this manuscript, either now or in the future?
- Do you hold or are you currently applying for any patents relating to the content of the manuscript?
- Have you received reimbursements, fees, funding, or salary from an organization that holds or has applied for patents relating to the content of the manuscript?
- Do you have any other financial competing interests?
- Do you have any non-financial competing interests in relation to this paper?

If you can answer no to all of the above, write 'I declare that I have no competing interests' below. If your reply is yes to any, please give details below.

I declare that I have no competing interests

I agree to the open peer review policy of the journal. I understand that my name will be included on my report to the authors and, if the manuscript is accepted for publication, my named report including any attachments I upload will be posted on the website along with the authors' responses. I agree for my report to be made available under an Open Access Creative Commons CC-BY license (http://creativecommons.org/licenses/by/4.0/). I understand that any comments which I do not wish to be included in my named report can be included as confidential comments to the editors, which will not be published.

I agree to the open peer review policy of the journal

To further support our reviewers, we have joined with Publons, where you can gain additional credit to further highlight your hard work (see: https://publons.com/journal/530/gigascience). On publication of this paper, your review will be automatically added to Publons, you can then choose whether or not to claim your Publons credit. I understand this statement.

Yes Choose an item.
