# Supplementary material for: Genome sequence of the corn leaf aphid (Rhopalosiphum maidis Fitch)
Source: Gigascience. 2019 Apr 6;8(4):giz033. doi: 10.1093/gigascience/giz033 (PMC6451198; doi:10.1093/gigascience/giz033)
Supplement: Reviewer_3_Report_Original_Submission -- Shuji Shigenobu — 12/6/2018 Reviewed [file giz033_reviewer_3_report_original_submission.pdf]

Reviewer Report

Title: Genome sequence of the corn leaf aphid (Rhopalosiphum maidis Fitch)

Version: Original Submission **Date:** 12/6/2018

Reviewer name: Shuji Shigenobu

Reviewer Comments to Author:

The authors reported a high-quality genome assembly of the corn leaf aphid, Rhopalosiphum maidis Fitch. This is the first chromosome-level reference genome for aphids. I congratulate the authors on the excellent genome assembly generated by the combination of long-read sequencing and Hi-C technology. This genomic information is undoubtedly a valuable resource for a wide range of research fields, including aphid biology, pest control research, and insect genomics. However, as outlined below, there are several issues that need to be addressed before the paper can be suitable for publication.

Major issues

Information on sampling and genome sequencing are not provided enough (pp.3-5). Since this study is the most successful genome sequencing in aphids thus far, detailed procedures would be useful for readers. I list specific points as follows:

* p.4: Around 50 ug of high molecular weight DNA was prepared … : High molecular weight (HMW) genome preparation is key to a successful genome project. How long was it? How did the authors evaluate the HMW genome? (e.g., pulse field electrophoresis)
* p.4: … using SMRT Cell template preparation kits … : Detailed procedures should be described. For example, what is the size selection method (e.g., BluePippin)? How much is the insert size of the resultant library?
* p.4: … run on the PacBio Sequel platform …: Detailed sequencing condition should be described. Which version of PacBio chemistry did they use? (e.g., M1 v2). How long was the movie time?
* p.5.: … using the Illumina TruSeq DNA sample preparation kit following the manufacturer's instructions: Detailed prep information should be provided. Was it a PCR-free library? How long was the insert size of the library?
* p.5: Transcriptome sequencing: Sample information should be provided. Which tissue? (Whole body?) Adults or nymphs?
* p5: Iso-seq: … for PacBio large-insert (15-20kb) library construction ... : It sounds weird. Iso-seq library should not be long-insert one. It is typically below several kb inserts. A standard PacBio Iso-seq preparation is composed of SMARTer PCR cDNA Synthesis and SMRTbell library construction. Did the author use a special technique for Iso-Seq? If it is the case, the detailed procedures should be described in the manuscript.
* They reported the genomes of R. maidis and Buchnera symbiont in this manuscript. I am wondering if the authors found a mitochondrial genome in the assembly as well?
* Although the authors did not emphasize in the current manuscript, one of the great things of this assembly is that it contains only a small fraction of ambiguous sequences (Ns) in the assembly thank to the long-read technology. I downloaded the sequence data and calculated as 469 kbp (0.14%) as Ns,

which is much less than precedent aphid genome assemblies reported. I think the authors can mention this point in the manuscript.
* Given the chromosome-level assembly, it is natural to ask which is the sex chromosome (X) among the four long scaffolds? (Aphid sex determination is XO system). Several labs identified X-linked genes in other aphids such as Acyrthosiphon pisum, which might be used to predict X chromosome of R. maidis. It would be great if the authors will include this analysis in the revised manuscript if possible. However, the identification of X chromosome might not be as straightforward as I expect; it's ok as is. I do not think lack of the X chromosome analysis should prevent publication and some researchers will address this issue in the future using the genome sequence described here.
* p7: Contaminated contig detection: They just searched against NCBI nt database using BLASTN. It sounds a naive strategy. I recommend read coverage should be considered as well. Alternatively, sophisticated software such as BlobTools can be used for contamination detection.
* p.9: Discussion on the gene numbers: The authors imply that the gene number difference among aphid species is associated with the genome size, but there are other possibilities. One of the likely causes is just the difference of gene prediction method between the genome projects. Actually, Thorpe and his colleagues reanalyzed 5 aphid genomes and annotated genes using the same pipeline and concluded that aphid genomes consistently encode similar gene numbers (P. Thorpe et al. 2018 GBE). I do not intend to request the authors to reanalyze R. maidis gene models from scratch, which is a big deal, but I request careful editing of the text in this part of the manuscript.
* p.10: Horizontal gene transfer detection: h score alone is not convincing. They mentioned " … manually checked for potential genome assembly errors …", but they did not mention the procedure and the criteria in detail. I still concern the possibility of the contamination.
Regarding Data Set hosted in GigaScience FTP, I have some suggestions to add more data discussed in the manuscript. I list the specific suggestions as follows:
* OrthoMCL ortholog table should be provided.
* It would be great if CDS fasta could be provided.
* It would be great if annotation files (InterPro search results and GO terms) were provided. They would be extremely useful for readers.
# Minor issues
* p3 anholocyclic: general audience (non-aphid researchers) may not be familiar with this word. It needs some explanation.
* Table 1: assembly size of R. maidis is described as 326.0 Mb â€” but I calculated the total length of the assembly from the downloaded data and found it 326,445,255 bp.
* p7: … aligning the initial assembly to the Buchnera reference genome...: The authors should specify which Buchnera is. NC_002528.1 is Buchnera aphidicola of the pea aphid (Acyrthosiphon pisim). Also, they should cite the corresponding paper (Shigenobu et al. 2000 Nature).
* p8: Secondary symbiont detection: They simply mentioned that they used BLAST. The details such as search parameters should be described.
* p8: In Gene prediction section: … completed proteomes of A.pisum, A. glycine … : what are the sources of the proteome sequences? AphidBase or NCBI RefSeq? Sometimes the gene set of AphidBase and NCBI is different each other.
Some typos:

* p6: Hi-C Libraries => Hi-C libraries
* p9: Ac. Pisum => Ac. pisum
* p11: Ac. Pisum => Ac. pisum
* p16 ref 32 extractino => extraction

Level of Interest

Please indicate how interesting you found the manuscript: Choose an item.

Quality of Written English

Please indicate the quality of language in the manuscript: Choose an item.

Declaration of Competing Interests

Please complete a declaration of competing interests, considering the following questions:

- Have you in the past five years received reimbursements, fees, funding, or salary from an organisation that may in any way gain or lose financially from the publication of this manuscript, either now or in the future?
- Do you hold any stocks or shares in an organisation that may in any way gain or lose financially from the publication of this manuscript, either now or in the future?
- Do you hold or are you currently applying for any patents relating to the content of the manuscript?
- Have you received reimbursements, fees, funding, or salary from an organization that holds or has applied for patents relating to the content of the manuscript?
- Do you have any other financial competing interests?
- Do you have any non-financial competing interests in relation to this paper?

If you can answer no to all of the above, write 'I declare that I have no competing interests' below. If your reply is yes to any, please give details below.

I declare that I have no competing interests.

I agree to the open peer review policy of the journal. I understand that my name will be included on my report to the authors and, if the manuscript is accepted for publication, my named report including any attachments I upload will be posted on the website along with the authors' responses. I agree for my report to be made available under an Open Access Creative Commons CC-BY license (http://creativecommons.org/licenses/by/4.0/). I understand that any comments which I do not wish to be included in my named report can be included as confidential comments to the editors, which will not be published.

I agree to the open peer review policy of the journal

To further support our reviewers, we have joined with Publons, where you can gain additional credit to further highlight your hard work (see: https://publons.com/journal/530/gigascience). On publication of this paper, your review will be automatically added to Publons, you can then choose whether or not to claim your Publons credit. I understand this statement.

Yes Choose an item.
